# The malaria testing and treatment landscape in the southern Lao People’s Democratic Republic (PDR)

**DOI:** 10.1186/s12936-017-1769-0

**Published:** 2017-04-25

**Authors:** ACTwatch Group, Angela Alum, Andrew Andrada, Julie Archer, Erick Auko, Katie Bates, Paul Bouanchaud, Meghan Bruce, Angela Camilleri, Emily Carter, Steven Chapman, Nikki Charman, Desmond Chavasse, Kevin Duff, Keith Esch, Anna Fulton, Kevin Duff, Keith Esch, Illah Evance, Anna Fulton, Hellen Gataaka, Tarryn Haslam, Emily Harris, Catharine Hurley, Whitney Isenhower, Beth Kangwana, Esther Kabui, Gloria Kigo, Irene Kyomuhangi, Aliza Lailari, Megan Littrell, Greta Martin, Erik Monroe, Julius Ngigi, Kathryn A. O’Connell, Ricki Orford, Carolyne Ochieng, Linda Ongwenyi, Stephen Poyer, Justin Rahariniaina, Lanto Razafindralambo, Christina Riley, John Rodgers, Tanya Shewchuk, Julianna Smith, Tsione Solomon, Raymond Sudoi, Martine Esther Tassiba, Katherine Thanel, Andria Rusk, Julianna Smith, Rachel Thompson, Mitsuru Toda, Marie-Alix Valensi, Vamsi Vasireddy, Cynthia Whitman, Saysana Phanalasy

**Affiliations:** 1 Population Services International, 1120 19th St NW Suite 600, Washington, DC 20036 USA; 2 Population Services International, PSI Lao PDR, T4 Road, Unit 61, Donkoi Village, Sisattanak District Vientiane, Lao People’s Democratic Republic

**Keywords:** Lao PDR, Case management, Private sector, Public private mix, Chloroquine, ACT, Anti-malarial

## Abstract

**Background:**

In the context of national and regional goals to eliminate malaria by 2030, the Center for Malaria Parasitology and Entomology in the Lao PDR is implementing strategies to ensure all malaria cases are detected and appropriately treated with first-line artemisinin combination therapy, artemether–lumefantrine (AL). Timely and relevant evidence to inform policies and strategies is needed to ensure the most effective and efficient use of resources, and to accelerate progress towards elimination goals. A 2015 outlet survey conducted in five provinces of the southern Lao PDR was the first of its kind to study the total market for malaria treatments and diagnostics. The sub-national outlet survey was designed to describe the market and to assess public and private sector readiness and performance for malaria case management. Additionally, key indicators were estimated among private outlets within districts with and without a Public Private Mix (PPM) programme.

**Results:**

Over half of anti-malarial stockists were public sector (65.1%). In the private sector, pharmacies most commonly stocked anti-malarials, although anti-malarials were also found in private health facilities, drug stores, general retailers, and itinerant drug vendors. Nearly all anti-malarial stocking public health facilities had AL (99.5%) and 90.8% had confirmatory testing. Fewer than half of anti-malarial stocking private outlets stocked AL (40.8%) and malaria testing (43.5%). Chloroquine has not been a first-line treatment for *Plasmodium falciparum* malaria since 2005 and *Plasmodium vivax* since 2011 yet private sector availability was 77.6% and chloroquine accounted for 62.2% of the total anti-malarial market share. AL and confirmatory testing availability were higher in private outlets in PPM (68.1, 72.6%) versus non-PPM districts (2.5, 12.1%). Chloroquine was available in 63.6% of PPM and 96.7% of non-PPM-district outlets, and was the most commonly distributed anti-malarial among private outlets in both PPM (61.7%) and non-PPM districts (99.1%).

**Conclusions:**

Public sector outlets in the southern Lao PDR are typically equipped to test and appropriately treat malaria. There is need to address widespread private sector availability and distribution of chloroquine. The PPM programme has improved private provider readiness to manage malaria according to national guidelines. However, supporting interventions to address provider and consumer behaviours are needed to further drive uptake.

**Electronic supplementary material:**

The online version of this article (doi:10.1186/s12936-017-1769-0) contains supplementary material, which is available to authorized users.

## Background

Important gains in malaria control have been achieved in recent years in the Lao, People’s Democratic Republic (PDR). Malaria admissions and deaths have declined substantially since 2000. However, a 2011 outbreak in the southern provinces has been associated with a spike in cases and deaths. Case numbers have not returned to the seasonal low levels observed prior to 2011, suggesting an ongoing outbreak [1]. In 2015, there were over 48,000 reported confirmed positive malaria cases, up from 38,131 cases in the previous year [1, 2]. Approximately 31% of the Lao PDR’s population of 6.6 million live in areas of high transmission and another 61% live in areas of low transmission. The vast majority (95%) of the malaria burden is concentrated in the southern five provinces. *Plasmodium falciparum* makes up 62% of the parasite species while *Plasmodium vivax* comprises the other 38% [2]. Artemether–lumefantrine (AL) was introduced as the first-line treatment for uncomplicated *P. falciparum* malaria in 2005 and *P. vivax* malaria in 2011.

The Lao PDR has set the goal of eliminating *P. falciparum* malaria by 2025 and all forms of malaria by 2030. In line with the World Health Organization’s (WHO) Strategy for Malaria Elimination in the Greater Mekong Subregion, the dual goal has been set to both interrupt transmission of *P. falciparum* in areas of multidrug resistance as well as reduce malaria transmission in high transmission areas to less than one case per 1000 population at risk by 2020 [1, 3].

Detecting and appropriately treating all malaria cases is critical to achieving elimination goals in the Lao PDR. In order to bolster proper testing and treatment practices, the Center for Malaria Parasitology and Entomology (CMPE) has devoted significant resources in recent years to ensuring that public health facilities are stocked with appropriate first-line artemisinin combination therapy (ACT) and malaria rapid diagnostic tests (RDTs). Malaria diagnosis has been free of charge in the public sector since the beginning of 2005 [1]. In 2010, CMPE began procuring RDTs capable of detecting both *P. falciparum* and *P. vivax* parasites, which increased the number of reported cases.

CMPE expanded access to appropriate test and treat services to the community-level through the training and equipping of Village Malaria Workers as well as some existing Village Health Volunteers for proper testing and treating of malaria in endemic areas. The Community Health Worker (CHW) programme for malaria case management using RDT and ACT was introduced in 2005. In 2016, a total of 5825 CHWs with malaria case management training and equipment were counted [1]. Activities aimed at strengthening the CHW network are highly prioritized and are scheduled to receive USD $4.2 million over the next 5 years [1].

CMPE has also extended access to appropriate malaria case management through leveraging the private sector. The Public Private Mix (PPM) programme commenced in 2008 as a way of introducing first-line ACT and RDT into the highly utilized private sector. Participating pharmacies and private for-profit facilities were supplied with AL and RDTs through the existing government supply chain. Participating outlets were permitted to sell the products at a small profit, though many providers reportedly chose to dispense AL free of charge [4]. The PPM pilot initially included 10 private clinics and 85 pharmacies from 8 districts across 4 provinces. By 2012, the programme expanded to 16 clinics and 245 pharmacies from 22 districts across 8 provinces [4]. According to government policy, only PPM pharmacies are authorized to dispense anti-malarials.

In line with the National Strategic Plan, CMPE and other implementing partners, will continue to address malaria case management gaps that are critical to achieving progress towards malaria elimination over the next 5 years. Timely and relevant evidence to inform case management policies and strategies is needed to ensure the most effective and efficient use of resources, and to accelerate progress towards elimination goals. However, substantial evidence gaps exist with respect to the total market for malaria testing and treatment in the southern Lao PDR, including case management readiness and performance of providers across the public and private sectors. Understanding the private sector in the Lao PDR will be particularly important given this is an important treatment channel [5].

The ACTwatch project is a multi-country research project launched in 2008 by Population Services International (PSI) and London School of Hygiene and Tropical Medicine (LSHTM) with support from the Bill and Melinda Gates Foundation. The goal of ACTwatch is to fill contemporary evidence gaps by collecting malaria case management market data on anti-malarial treatments and malaria diagnostics in both the private and public sector. ACTwatch provides timely, high quality and relevant anti-malarial market data so as to inform and monitor national, regional and global malaria case management policy, strategy and funding decisions [6, 7].

The 2015 ACTwatch outlet survey was the first of its kind to be conducted in the Lao PDR. The objective of this paper is to provide practical evidence to inform the malaria elimination strategy and policy in the Lao PDR. The paper describes the total market for malaria treatments and diagnostics in the five southern provinces of the Lao PDR with highest malaria burden. Key indicators are presented including a description of the market, readiness to test and treat in public and private sectors, anti-malarial market share, and provider knowledge. Evidence on the total market, as well as on outcomes associated with the PPM programme, will point to recommendations for rapidly improving coverage of appropriate malaria case management in the southern Lao PDR.

## Methods

### Design and sampling

A representative cross-sectional outlet survey was conducted amongst a sample of outlets stocking malaria testing and/or treatment in five southern provinces in the Lao PDR (Savannakhet, Champasack, Salavanh, Sekong and Attapeu). According to the ACTwatch methodology, outlets are included in the survey if they have the ‘potential’ to sell or distribute anti-malarials or diagnostic testing. This includes outlets that may not typically be expected to stock anti-malarial treatments, such as general retailers, village shops, or itinerant drug vendors. However, it is recognized that in many countries these outlets can operate as vendors for anti-malarial commodities, either illegally or/and outside of the formal health system. As such, in many instances outlets are included in the sample as a means to confirm their role or presence in a given country’s anti-malarial and diagnostic market. These outlets may differ on a country by country basis, but overall broad categories are used to define public and private sector outlets.

In the Lao PDR, outlets with the potential to sell or distribute included public health facilities (provincial hospitals, district hospitals and health centers) and CHWs (village malaria workers and village health volunteers). Private sector outlets included private for-profit health facilities and pharmacies. The private for-profit facility category consisted of private hospitals, clinics and diagnostic laboratories. The pharmacy category consisted of clinical pharmacies and level 1, 2 and 3 pharmacies. Clinical pharmacies are those that offer clinical and pharmaceutical services despite only being licensed to offer pharmaceutical services. Level 1 pharmacies are large, can act as wholesalers and have pharmacists on staff to advise patients on treatment. Level 2 pharmacies, while smaller, can still act as wholesalers but only sometimes have pharmacists on staff to advise patients. Level 3 pharmacies are small and the owner, who is not a pharmacist, is renting a pharmacy license from a pharmacist. Informal private sector outlets were also included in the outlet survey, including drug stores, general retailers and itinerant drug vendors. Drug stores were defined as unregistered rural market or home stalls that primarily sold treatments and were not necessarily staffed by a trained pharmacist. General retailers consisted of grocery stores and village shops selling fast-moving consumer goods. Itinerant drug vendors were unregistered mobile drug vendors generally catering to mobile migrant communities. Outlets that did not serve the general public were excluded from the outlet survey, however military and police facilities that served the general public were included.

The primary sampling approach taken for ACTwatch outlet surveys entails sampling a set of administrative units (geographic clusters) with a population of approximately 10,000–15,000 inhabitants. Clusters are selected with cluster probability of selection proportionate to size (PPS). A census of all outlets with the potential to sell  or distribute anti-malarials is then conducted in sampled clusters, given a sampling frame for all potentially eligible outlet types is not available. The most appropriate administrative unit in the Lao PDR matching the desired population size was a village group. The village group was an administrative unit with populations smaller than districts but larger than individual villages. Most village groups include between five and ten villages. Village groups were selected with PPS using population estimates obtained from the Lao National Statistics Centre.

Given this was the first ACTwatch outlet survey implemented in the Lao PDR, and previous information on the number of outlets or first-line treatment was not available, a series of calculations and assumptions were made to identify minimum sample size requirements. The sample size was developed to estimate with precision (±7.5 percentage points) the proportion of outlets with first-line anti-malarial treatments available, among outlets with anti-malarial(s) in stock on the day of the survey for all public health facilities and private for-profit facilities and pharmacies. The required sample size was calculated in three steps: (1) determine the required number of anti-malarial-stockists; (2) determine the number of clusters (village groups) for the census to arrive at this number of outlets; (3) determine the required number of village groups. Available information on numbers of public and private sector outlets per village group were used to determine the optimal number of clusters for the outlet survey. National outlet lists provided by the Food and Drug Department and Health Care Department were used to determine the number of public health facilities and private registered outlet types per village group. On average, in the Southern Lao PDR there were around 3.3 public health facilities and regulated private outlets per village group. Based on these assumption, a sample size of 77 village groups was selected in order to estimate key indicators on availability of first-line treatment and malaria testing with 95% confidence and a maximum tolerable error of 5%. A design effect of 2 was used to account for cluster sampling in the context of what was anticipated to be a high degree of homogeneity in the anti-malarial market within and across clusters.

To estimate indicators within the private sector with precision, the boundary for the census of pharmacies and private for-profit health facilities was extended to the district level. This ‘booster sample’ of formal private sector outlets covered all pharmacies and private for-profit health facilities within 41 of the 42 distributed in the five study provinces allowing for a sufficient sample size to allow for precise comparisons between these important but less common facility types Within each selected cluster, a census of all the aforementioned outlets was conducted. To identify outlets, interviewers would walk systematically through each of the selected clusters looking for relevant outlets. Lists of registered outlets, such as public health facilities or pharmacies, were obtained prior to the data collection and used to help identify outlets. To identify itinerant drug vendors, congregation points or locations were identified using key informant interviews. These providers were approached by interviewers and asked if they had already participated in the survey to avoid duplication. Outlets were screened to assess eligibility for the outlet survey. Outlets were eligible for a provider interview and malaria product audit if they met at least one of three study criteria: (1) one or more anti-malarials reportedly in stock on the day of the survey; (2) one or more anti-malarials reportedly in stock within the 3 months preceding the survey; and/or (3) had malaria blood testing (microscopy or RDT).

### Measures

The outlet survey was conducted using a paper questionnaire. The questionnaire was translated from English to Lao and then back to English to confirm valid translations in Lao. Outlets meeting eligibility criteria noted above were invited to participate in the survey. Following informed consent procedures, an audit of all available anti-malarial treatments and RDTs was conducted. Anti-malarial audit information included formulation, package size, brand name, active ingredients and strengths, manufacturer, country of manufacture, reported sale/distribution in the week preceding the survey, retail price, and wholesale price. The RDT product audit collected similar information, but excluded questions on pack size, formulation, strength and active ingredients. In addition to the product audit, a series of questions was administered to the senior-most provider regarding malaria case management knowledge and practices, as well as provider training and qualifications and reporting on malaria case load data (Additional file 1: Survey questionnaire in English, Additional file 2: Survey questionnaire in Lao language). Geo-coordinates were recorded for each outlet using a handheld Global Positioning System (GPS) unit. Up to three visits were made to all outlets to complete the screening process, audit, and provider interview, as needed (e.g. where outlets were closed or providers were not available).

### Training and data collection

All standardized training materials were adapted to fit within the context of the southern Lao PDR. A 1-week training of trainers was held in October 2015, followed by a 2-day pilot to test the ACTwatch outlet survey instruments in the Lao PDR context. A 6-day data collector training was then held, followed by a 2-day data collector field exercise in order to give newly trained data collectors practice with the study methodology and tools. High performers identified during data collector training were selected for a further 3 days of supervisor and quality controller training.

Five data collection teams were created after the conclusion of training. Each team was comprised of one supervisor, one quality controller and three to four data collectors. Two PSI/Lao platform and two research agency staff offered higher-level logistical and data quality supervision. Field operations were supervised and managed by an ACTwatch team-member.

A 4-day double data entry and data coding training was also conducted. A supervisor, two coders and ten data entry clerks were trained in appropriate coding, translation and data entry techniques. The supervisor oversaw all data coding and entry processes and notified ACTwatch staff if any issues arose.

Peak malaria transmission season in the Lao PDR is July–October. Due to delays in study approval, data collection took place between November 18th and December 29th, 2015. Upon a data collection team’s arrival to a district within a selected cluster, data collector team supervisors met with district officials to crosscheck their list of public and formal private sector outlets with that of the government list. Data collection teams then travelled to the selected cluster and met with the village group head. These meetings generally yielded sketch maps of the villages, which were useful for data collection teams during the census process.

A Microsoft Access (Microsoft Corporation, Redmond, Washington, USA) database with built-in range checks was used to conduct double data entry from physical questionnaires shipped from the study area to Vientiane. Daily supervisor and data collector monitoring sheets were collated in a Microsoft Excel (Microsoft Corporation, Redmond, Washington, USA) spreadsheet, which along with the physical questionnaires, were used to triangulate entered data.

### Protection of human subjects

The 2015 outlet survey protocol received ethical approval from the National Ethics Committee for Health Research in the Lao PDR (approval number 059 NIOPH/NECHR). Provider interviews and product audits were completed only after administering a standard informed consent form and provider consent to participate in the outlet survey. Providers had the option to end the interview, which was conducted in a private place, at any point during the outlet survey. Standard measures were employed to maintain provider confidentiality and anonymity. Fieldworker training instructed and assessed understanding of these standard precautions for protecting human subjects among all people working on the study. All information provided by respondents was strictly confidential and used only for study purposes. All data collectors were instructed and monitored to ensure that they did not share information about individual outlets or providers with community members or local leaders. Information about individual outlets was not shared with any national authorities. Results were not linked to individual providers or outlets. Respondent names and outlet names were not stored with the final clean data.

### Data analysis

Stata 13 (StataCorp, College Station, Texas, USA) was used to analyse data imported from the Access database. Survey settings were used to account for the study design and included sampling weights, calculated as the inverse of probability of village group selection.

Standard ACTwatch indicators were calculated [6, 7]. Briefly, anti-malarials were classified as ACT, non-artemisinin therapy, and oral or non-oral artemisinin monotherapy.

Availability was defined in this study as the proportion of outlets stocking at least one anti-malarial, among censused outlets. Other anti-malarial and RDT availability categories were calculated but restricted to those outlets where at least one anti-malarial was audited. For example, ACT availability (the proportion of ACT-stockists) was measured as the number of ACT-stockists in the numerator and the number of anti-malarial stockists in the denominator.

Market share was defined as the relative distribution of the anti-malarials to individual consumers in the week preceding the survey. In order to allow for meaningful market share comparisons between products, information about anti-malarial distribution was standardized to the adult equivalent treatment dose (AETD). AETD is the amount of active ingredient necessary to treat a 60 kg adult according to WHO treatment guidelines [8]. Volumes distributed were calculated by converting provider reports on the number of anti-malarials sold in the week prior to the survey into AETDs. Volumes were, therefore, the number of AETDs sold or distributed by a provider in the 7 days prior to the survey. All dosage forms were considered in measuring volumes so as to provide a complete assessment of anti-malarial market share. Private sector booster sample outlets were excluded from market share calculations to avoid over-estimating the role of the private sector.

Median private sector price per AETD was calculated for the first-line ACT and for chloroquine, and for RDT testing including consultation and service fees. Interquartile range (IQR) was calculated to demonstrate price dispersion. Anti-malarial and RDT price was collected in Lao Kip and converted to the US dollars based on official exchange rates for the 6-week data collection period.

Provider knowledge was assessed by administering knowledge questions to the senior most provider at all anti-malarial-stockists. The senior-most provider was questioned because he or she generally holds the most knowledge regarding diagnosis and prescription practices at the outlet. In all but one case, data collectors were able to interview the senior-most provider at eligible outlets. The one outlet in which the senior-most provider was not available was dropped from the data set. Provider knowledge was assessed in two ways—knowledge of national first-line treatment and dosing regimen for uncomplicated *P. falciparum*/*P. vivax* malaria for a 60 kg adult as well as dosing regimen for uncomplicated *P. falciparum*/*P. vivax* malaria for a 60 kg adult. Dosing regime knowledge assessment components included questions on the number of tablets per dose, number of times per day and number of days in the regimen.

Among the 41 study districts, there were 25 districts with and 16 districts without the PPM programme. PPM programme designation for each district was obtained from CMPE. Private for-profit facilities or pharmacies were defined as being part of the PPM programme according to their designated location. Outlets that were located in PPM districts were designated as ‘PPM outlets’, and those outlets that were located in non-PPM districts were defined as ‘non-PPM’ outlets. Only private for-profit facilities and pharmacies were classified as PPM or non-PPM outlets, given these were the outlet types targeted by the programme. Other outlet types, such as public health facilities and general retailers were excluded from the PPM definition. Key private sector indicators including availability, anti-malarial market share, and price were calculated among the private sector PPM and non-PPM outlets.

## Results

A total of 7586 outlets were screened for availability of anti-malarials and/or malaria blood testing services. Of screened outlets, 725 were stocking anti-malarials or malaria blood testing on the day of the survey or within the past 3 months, and 724 were subsequently interviewed, as one eligible respondent was not available for interview. A total of 1666 anti-malarial and 483 RDT products were audited (Additional file 3: detailed sample description).

### Availability

**Fig. 1:**
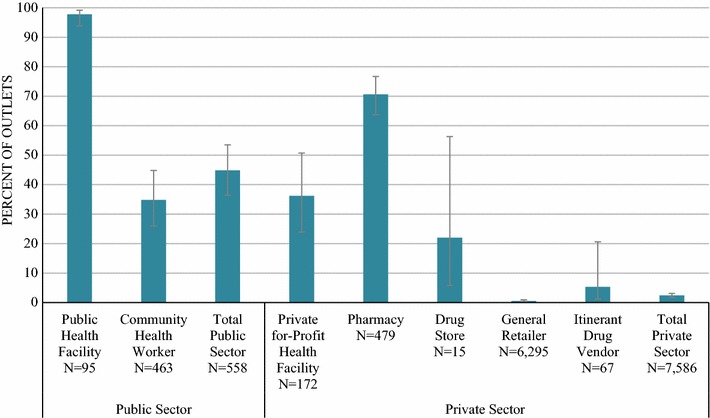
Percentage of all screened outlets with at least one anti-malarial in stock on the day of the survey

Across all screened outlets in the public sector (N = 558), 97.8% of public health facilities and 34.8% of CHWs stocked at least one anti-malarial on the day of survey. Across all screened outlets in the private sector (N = 7028), availability of any anti-malarial was 6.5%. Private sector availability was highest among pharmacies (70.6%; N = 479) followed by private for-profit facilities (36.2%, N = 172), drug stores (22.0%, N = 15) and itinerant drug vendors (5.3%, N = 67). Of the 6295 general retailers screened, 0.5% stocked at least one anti-malarial (Fig. 1).

### Market composition

**Fig. 2:**
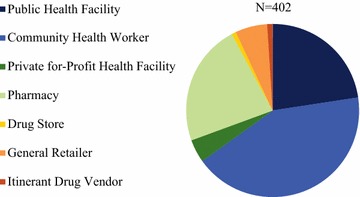
Anti-malarial market composition

Figure 2 illustrates the relative distribution of outlets with at least one anti-malarial in stock on the day of survey (N = 402). Among anti-malarial stockists, 67.2% were public sector outlets, made up of CHWs (42.5%) and public health facilities (22.6%). 22.8% of anti-malarial service delivery points were pharmacies. General retailers and private for-profit facilities each accounted for 6.0 and 4.3% of the market composition respectively. Itinerant drug vendors accounted for just 1% of the anti-malarial market composition.

### Availability of anti-malarials and blood testing

Table 1 illustrates availability of different types of anti-malarials and malaria blood testing, among outlets that were stocking at least one anti-malarial. Among anti-malarial stockists in the public sector (N = 236), availability of the national first-line ACT (AL) was 88.8%, with almost universal availability among public health facilities (99.5%). Availability among CHWs was 83.1%. In the anti-malarial stocking private sector (N = 394), 63.3% of private for-profit facilities and 51.7% of pharmacies stocking at least one anti-malarial had AL in stock. All AL available in the public and private sectors was considered quality-assured given that all audited AL products were listed on the World Health Organization’s pre-qualification list and/or the Global Fund list of approved anti-malarials.

**Table 1:** Availability of anti-malarials and malaria blood testing, among anti-malarial stockists

	Public health facility	Community health worker	Public sector total	Private for-profit facility	Pharmacy	General retailer	Private sector total
	% (95% CI)	% (95% CI)	% (95% CI)	% (95% CI)	% (95% CI)	% (95% CI)	% (95% CI)
Proportion of outlets stocking:	N = 91	N = 145	N = 236	N = 56	N = 309	N = 23	N = 394
Any national first-line ACT (AL)	99.5 (97.1, 99.9)	83.1 (72.3, 90.3)	88.8 (82.0, 93.2)	63.3 (45.8, 77.9)	51.7 (40.0, 63.2)	3.1 (0.5, 17.0)	40.8 (31.1, 51.2)
Chloroquine	4.6 (1.9, 10.6)	19.2 (11.2, 31.0)	14.1 (8.9, 21.7)	49.3 (33.7, 65.0)	74.6 (67.0, 80.9)	96.9 (83.0, 99.5)	77.6 (71.2, 83.0)
Primaquine	6.7 (1.57, 24.17)	0.0 –	2.8 (0.6, 12.13)	0.0 –	0.0 –	0.0 –	0.0 –
Oral artemisinin monotherapy	0.0 –	0.0 –	0.0 –	2.7 (0.5, 14.5)	0.0 –	0.0 –	0.3 (0.0, 1.6)
	**N = 91**	**N = 154**	**N = 255**	**N = 58**	**N = 327**	**N = 30**	**N = 424**
Any malaria blood testing	90.8 (77.4, 96.6)	78.4 (66.7, 86.9)	82.4 (74.2, 88.3)	77.0 (64.3, 86.2)	55.7 (44.2, 66.7)	6.1 (1.6, 20.6)	43.5 (34.2, 53.3)
Malaria microscopy	23.1 (14.9, 34.0)	0.0 –	7.3 (4.6, 11.4)	16.0 (9.4, 25.8)	0.3 (0.0, 1.7)	0.0 –	1.7 (1.0, 2.9)
Rapid diagnostic tests	85.4 (73.5, 92.5)	78.4 (66.7, 86.9)	80.6 (72.3, 86.9)	77.0 (64.3, 86.2)	55.7 (44.2, 66.7)	6.1 (1.6, 20.6)	43.5 (34.2, 53.3)

*AL* artemether–lumefantrine

Availability of chloroquine among anti-malarial stocking public health facilities was 4.6 and 19.2% among CHW. In the private sector, 77.6% of all anti-malarial stockists had chloroquine available. Chloroquine availability was highest among anti-malarial stocking general retailers (96.9%), followed by pharmacies (74.6%) and private for-profit facilities (49.3%).

Primaquine was generally not available across the public or private sector, with the exception of anti-malarial stocking public health facilities (6.7%). Of the 7586 outlets screened, only one box of oral artemisinin monotherapy was found.

Among public health facilities, 90.8% of anti-malarial stockists had malaria blood testing; 85.4% had RDTs in stock on the day of survey and 23.1% had malaria microscopy. Among CHWs, 78.4% had RDTs in stock on the day of survey. Within the private sector, malaria blood testing was available in 77.0% of private for-profit facilities and 55.7% of pharmacies, and typically stocked RDTs.

### Anti-malarial market share

**Fig. 3:**
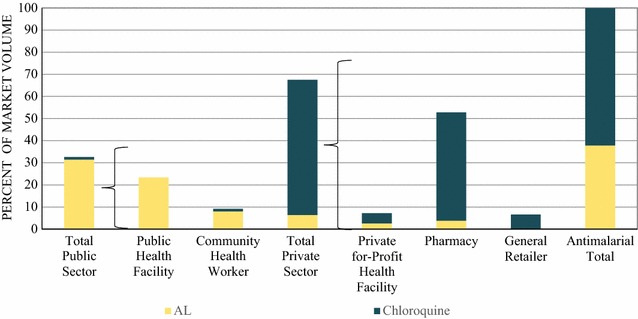
Anti-malarial market share

Figure 3 shows the relative anti-malarial market share in the public and private sector. All anti-malarials reportedly distributed in the southern Lao PDR were either AL or chloroquine, and most anti-malarials distributed were chloroquine treatments (62.2%). Almost all the chloroquine distributed was through the private sector. In contrast, AL was almost exclusively distributed by the public sector.

The public sector accounted 32.3% of the total anti-malarial market share, including public health facilities (23.4%) and CHWs (8.9%). Private sector market share was 64.5 and 49.9% of all anti-malarials distributed were distributed by pharmacies. Private for-profit facilities and general retailers each accounted 7.2 and 6.6% of the market share, respectively.

### Provider anti-malarial treatment knowledge

Table 2 illustrates provider knowledge to correctly state the national first-line treatment for uncomplicated *P. falciparum* or *P. vivax* malaria. Provider knowledge was 77.9% in the public sector and 40.4% in the private sector. Correct knowledge of the dosing regimen was 58.2% in the public sector (N = 255) and 30.2% in the private sector (N = 424). Provider knowledge was highest among public health facilities regarding the first-line treatment (89.5%) and first-line dosing regimen (73.0%). Among pharmacies, 49.5% could correctly state the first-line treatment for *P. falciparum* or *P. vivax* malaria.

**Table 2:** Provider anti-malarial treatment knowledge by outlet type

	Public health facility	Community health worker	Public sector total	Private for-profit facility	Pharmacy	General retailer	Private sector total
	% (95% CI)	% (95% CI)	% (95% CI)	% (95% CI)	% (95% CI)	% (95% CI)	% (95% CI)
Proportion of providers who:	N = 91	N = 164	N = 255	N = 58	N = 327	N = 30	N = 424
Correctly state the national first-line treatment for uncomplicated *P. falciparum/vivax* malaria, AL	89.5 (81.7, 94.2)	72.6 (64.7, 79.3)	77.9 (72.0, 82.9)	78.7 (63.8, 88.5)	49.5 (39.0, 60.0)	8.1 (2.7, 21.9)	40.4 (31.6, 49.8)
Correctly state the first-line (AL) dosing regimen for uncomplicated *P. falciparum/vivax* malaria for an adult	73.0 (60.7, 82.5)	51.3 (41.0, 61.5)	58.2 (49.6, 66.3)	66.4 (51.1, 78.8)	36.5 (28.0, 46.0)	6.1 (1.6, 20.6)	30.2 (23.1, 38.2)

*AL* artemether–lumefantrine

### Chloroquine insights

Most of the chloroquine distributed was in tablet formulation (94.8%), and other formulations included injections (5.2%) and syrups (<1%). The most commonly available chloroquine product was Maraquine^®^, a tablet produced in the Lao PDR by CBF pharmaceuticals. Maraquine^®^ accounted for 74.9% of all audited chloroquine products and for 50.5% of all anti-malarials distributed. Amongst all anti-malarial stocking private sector outlets, 28.9% of providers reported chloroquine was the most effective treatment for uncomplicated malaria. 32.7% of private sector providers reportedly recommended chloroquine most often for treatment of uncomplicated malaria in adults (Additional file 4).

### Key indicators among private sector outlets in PPM and non-PPM districts

This sub-section presents results among anti-malarial stockists located in designated PPM districts and in non-PPM districts (Table 3). Among the 264 private pharmacies and for-profit health facilities in PPM districts, 68.1% were stocking the AL and 72.6% were stocking malaria blood testing. Availability of AL in 101 pharmacies and private clinics in outlets located in non-PPM districts was 2.5%. Only 12.1% of pharmacies and private clinics in non-PPM districts had any malaria blood testing. 96.7% of anti-malarial stockists in non-PPM districts were stocking chloroquine compared to 63.6% in PPM districts.

**Table 3:** Key Indicators among pharmacies and private for-profit health facilities in PPM versus non-PPM districts

	PPM district outlets	Non-PPM district outlets
	% (95% CI)	% (95% CI)
Proportion of anti-malarial stockists with:	N = 264	N = 101
Any national first-line ACT (AL)	68.1	2.5
	(59.7, 75.4)	(0.9, 6.8)
Chloroquine	63.6	96.7
	(56.5, 70.2)	(92.3, 98.6)
	**N = 275**	**N = 110**
Any confirmatory testing	72.6	12.1
	(66.5, 78.0)	(6.6, 21.0)
**Proportion of providers frwho:**	**N = 275**	**N = 110**
Correctly state the national first-line treatment for uncomplicated *P. falciparum/vivax* malaria	65.0 (57.3, 72.0)	15.0 (7.6, 27.6)
Correctly state the first-line dosing regimen for uncomplicated *P. falciparum/vivax* malaria for an adult	51.0 (43.9, 58.0)	6.1 (2.7, 13.4)
	**N = 155**	**N = 36**
Report receiving a supervisory or regulatory visit within the past year	74.5 (61.6, 84.3)	17.0 (4.1, 49.4)
**Median price**	**Median** **[IQR]** ^**(N of Anti-malarials)**^	**Median** **[IQR]** ^**(N of Anti-malarials)**^
National first-line ACT (AL) AETD #	$0.00	$0.00
	[0.00–0.00] ^(516)^	[0.00–0.00] ^(3)^
Chloroquine AETD #	$0.62	$0.62
	[0.62–0.62] ^(173)^	[0.47–0.62] ^(103)^
Rapid diagnostic test	$0.00	$3.12
	[0.00–0.25] ^(216)^	[2.50–3.75] ^(38)^

*AL* artemether–lumefantrine, *AETD* adult equivalent treatment dose,* IQR* interquartile range

Provider knowledge of the first-line treatment for uncomplicated *P. falciparum* or *P. vivax* malaria was 65.0% in private sector outlets in PPM districts and 15.0% in the non-PPM districts. In PPM districts, 51.0% of providers correctly stated the first-line dosing regimen for uncomplicated *P. falciparum* or *P. vivax* compared with only 6.1% of providers in private sector non-PPM district outlets. Providers that reportedly received a supervisory or regulatory visit within the past year was 74.5% in private sector outlets in PPM districts and 17.0% in non-PPM districts.

AL was reportedly provided free-of-charge in private sector PPM district outlets. The retail price of chloroquine was the same ($0.62) in private sector outlets in PPM and non-PPM districts. In PPM districts, RDTs were provided free-of-charge in the private sector. The median price of an RDT in non-PPM district private sector outlets was $3.12.

**Fig. 4:**
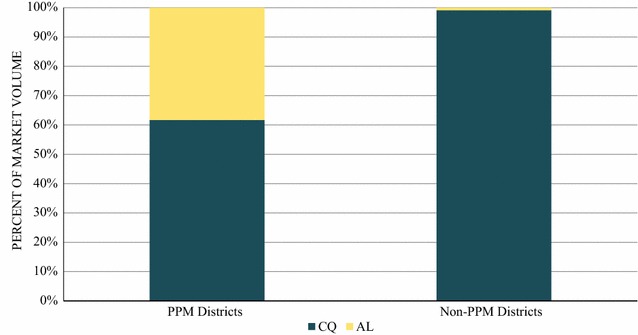
Anti-malarial market share within pharmacies and private for-profit health facilities in PPM versus non-PPM districts

Figure 4 illustrates the total anti-malarial distribution among private for-profit facilities and pharmacies located within the PPM and non-PPM districts. Chloroquine distribution was 99.1% among private sector outlets located in the non-PPM districts and 61.7% in outlets located in the PPM district. Distribution of AL in the week prior to the survey was only observed among the private sector outlets located in PPM districts (38.3%).

## Discussion

The 2015 outlet survey was the first anti-malarial market survey of its kind implemented in the southern Lao PDR. The outlet survey provided a complete picture of the malaria testing and treatment landscape across the public and private sectors with information on availability, price and market share as well as provider knowledge. The data show strong public sector readiness for proper malaria case management. Findings point to recommendations for rapidly improving coverage of appropriate malaria case management by reducing availability and market share of chloroquine in the private sector and to expand the PPM programme.

### Public sector readiness for appropriate malaria case management

Findings from the 2015 ACTwatch outlet survey show high public sector readiness for appropriate malaria case management in the southern Lao PDR. Nearly all public health facilities stocked the national first-line ACT, and confirmatory testing was available in over 90% of anti-malarial stocking public facilities.

The reach of the public sector has been extended to the community-level through the training and equipping of village malaria workers and village health volunteers with malaria case management skills and supplies. CHWs are playing an important part in provision of malaria case management services. They accounted for over 40% of all anti-malarial stockists, and distributed nearly 10% of all anti-malarials. Maintaining a network of trained and equipped CHWs is part of the strategy in the Lao PDR for achieving high malaria case management coverage and ultimately malaria elimination. Key challenges to be addressed as the Lao PDR attempts to achieve its goal of eliminating malaria by 2030 include gaps in CHW motivation and retention, training and maintaining supervision [1]. Results from this outlet survey suggest that in addition to these challenges, the availability of the non-first-line drug, chloroquine, must be addressed as this was available among one in five CHWs.

Primaquine is included in the national treatment guidelines as part of the first-line treatment for *P. falciparum*/*P. vivax* along with AL [9]. The results showed how availability of primaquine was negligible across all the public sector. At the time of the survey, primaquine had not been widely procured or distributed due primarily to concerns of adverse health reactions in patients with glucose-6 phosphate dehydrogenase (G6PD) deficiency. Two primaquine products were audited in the 2015 outlet survey and these were found in public district hospitals. The availability of primaquine in these outlets was likely due to a 2015 WHO-supported pilot study, which was conducted to assess G6PD testing and primaquine dispensing capabilities at selected district hospitals in three provinces. The presence of these products likely reflect leftover stock from the pilot study. Wider procurement of G6PD tests and primaquine is planned for 2016 as the national strategy expands to introduce primaquine more widely to treat *P. falciparum* and radically cure *P. vivax* infections in patients without G6PD deficiency [1, 10].

### Role of the private sector in appropriate malaria case management

The private sector plays an important role in malaria case management in the southern Lao PDR, as results from this 2015 outlet survey show that the private sector was responsible for approximately 60% of all anti-malarial distribution, a finding corroborated by other population based research [5]. Indeed, the private sector has played a predominant role in malaria case management in other countries in the Greater Mekong Subregion (GMS) region, including neighboring Cambodia [11, 12]. In the Lao PDR, the private sector for malaria case management includes both formal, regulated outlet types like private for-profit facilities and pharmacies as well as informal, unregulated outlet types such as drug stores, general retailers and itinerant drug vendors. Pharmacies were the most common type of private outlet stocking anti-malarials during the 2015 outlet survey, and accounted for nearly one in four anti-malarial stockists.

The private sector was generally less well-equipped to test and appropriately treat malaria infections as compared with the public sector. Fewer than half of anti-malarial-stocking private sector outlets were stocking the national first-line ACT, and fewer than half had confirmatory testing available. The majority of private sector outlets had the non-first-line drug, chloroquine, in stock.

### Widespread availability and use of chloroquine

Replaced by AL as the first-line treatment for *P. falciparum* in 2005 and *P. vivax* in 2011, chloroquine is now part of the national treatment guidelines as a second-line treatment for uncomplicated *P. vivax, Plasmodium ovale* and *Plasmodium malariae* infections. However, the availability of the second-line drug, chloroquine, should be limited, and with the drug found primarily in public health facilities equipped to detect and manage AL treatment failure. The results from this outlet survey illustrate how 10 years after the change in first-line treatment for *P. falciparum* and 5 years after the change in first-line treatment of *P. vivax*, chloroquine remained widely available and distributed, particularly in the private sector. The widespread popularity of chloroquine has been documented elsewhere [13], and its common presence on the market suggests it is being distributed as a first-line for treatment of uncomplicated malaria.

One driver of chloroquine popularity in the Lao PDR could be the accessibility of Maraquine^®^, an inexpensive, chloroquine tablet pre-packaged for individual treatment and manufactured locally by CBF pharmaceuticals (see Additional file 5). The Lao script makes the packaging understandable and recognizable to providers and potential customers alike. Maraquine^®^ accounted for three-quarters of all chloroquine audited during the outlet survey, and accounted for half of all anti-malarials distributed in the southern Lao PDR. As a widely available product, Maraquine^®^ represents a significant roadblock to AL uptake in the Lao PDR’s private sector. Further research is required to understand Lao consumer and provider preferences for this product, and new strategies are necessary to curtail the consumption of chloroquine and promote the use of the recommended first-line treatments, especially in the private sector.

### Public private mix

Significant efforts have been made in the southern Lao PDR to engage the private sector towards improving provider practices. The PPM was launched in 2008 with the aim of supporting, rather than discouraging pharmacies and for-profit health facilities to manage malaria cases appropriately. As such, the PPM programme has expanded access to proper testing and treatment in the highly utilized private sector [4]. On a promising note, the 2015 outlet survey demonstrated that the PPM programme had higher availability of first-line treatment and confirmatory blood testing as compared with private outlets that were not part of the PPM programme. In 2015, nearly all of the AL distributed by the private sector was distributed by outlets located in the designated PPM districts, and private sector availability of confirmatory testing was largely restricted to PPM districts. This suggests the PPM programme has potential for wider reach and impact with the addition of supporting interventions to address provider and consumer behaviour.

Despite high coverage with training and supervision, as well as moderate levels of provider knowledge regarding first-line treatment, chloroquine was still commonly stocked and distributed by these PPM providers. This suggests a delay in the uptake of subsidized anti-malarials, a finding that has been widely documented by other countries in the region [14–16]. Cambodia provides an example of a programme with a long history of subsidized first-line treatment in the private sector, and through an increasingly regulated private sector channel. Repeated outlet surveys have shown that while ACT availability has increased, market share has been slower to follow suit [17]. Evidence has pointed to the importance of necessity of considering provider and consumer factors that may influence first-line treatment uptake, as well as the national regulatory environment.

The market share findings point to the need for new strategies, or an intensification of existing ones, to completely remove chloroquine from the market. One important barrier to consider is the recommended price of the subsidised AL treatment. Providers participating in the PPM project may lack adequate financial incentives as compared with other subsidy models [18–21]. As part of the PPM programme, providers are permitted to charge 1000 Lao Kip ($0.12) for a treatment dose of AL and 2000 Lao Kip ($0.25) for an RDT [4]. By comparison, the median price of a treatment dose of chloroquine was $0.62. Although private sector providers may be stocking AL, they may be financially incentivized to dispense chloroquine given the profit margins they make, especially in light of the evidence that they typically distribute AL free-of-charge. Providers have reported that they can offset free distribution of AL by making profits on accompanying goods including vitamins and paracetamol, suggesting that profit is indeed important [4]. Future strategies may want to consider addressing provider financial incentives as well as consumer willingness to pay.

There is also likely a need for supporting interventions to drive consumer awareness and demand for AL [21]. Indeed, studies have suggested that customer demand influences retailer ACT dispensing behaviour [22], such that the odds of a patient receiving first-line treatment was found to be significantly associated with patient demand across both public and private sector facilities [23]. However, in general very little is known about malaria treatment-seeking behaviour and drivers of consumer behaviour in the Lao PDR. Though some key reviews were published in the 1990s [24, 25], evidence gaps exist. Effective strategies to drive demand for ACT will require additional evidence about consumer preferences and behaviours.

### Oral artemisinin monotherapy

Oral artemisinin monotherapy poses a serious threat to the continued efficacy of artemisinins in the Lao PDR and across the region, and as such this anti-malarial was banned in the Lao PDR in 2008. This ban has been sporadically enforced by the Food and Drug Department. In addition to ban enforcement, the promotion of free first-line ACT, initially in the public sector and now through the PPM programme, has been the main tool used to reduce the availability of oral AMT in the Lao PDR.

Previous studies have documented availability of oral artemisinin monotherapy in the private sector of GMS countries, including the Lao PDR [26]. Over 7500 outlets were screened during the 2015 outlet survey and just one box of oral artemisinin monotherapy (artesunate tablets) was found. Outlet survey results were consistent with recent research that has demonstrated a marked decrease in oral artemisinin monotherapy availability over time [13]. The removal of oral artemisinin monotherapy from the market in the Lao PDR is a success shared by neighboring Cambodia [12], but this remains a serious problem in another GMS country, Myanmar [27].

## Limitations

The ACTwatch outlet survey design has limitations that have been documented elsewhere [6, 28]. Specific to the outlet survey in the Lao PDR, data collection was conducted just after peak malaria season (July–October), between mid-November and the end of December, 2015. Outlet surveys are ideally conducted during peak transmission season to avoid fluctuations in stocking key commodities that may occur outside of peak season.

The outlet survey entails an audit of all available malaria commodities. Providers may have chosen to hide certain anti-malarial products. However, similar results were obtained through use of a mystery client study design implemented in the southern Lao PDR [13], suggesting that the outlet survey findings regarding very low levels of oral artemisinin monotherapy availability are valid.

The outlet survey was not designed to evaluate the PPM programme. PPM district status was determined post-data collection and analyses examined private sector readiness and performance in districts with and without the PPM programme. More rigorous evaluation of the PPM programme is needed, with a study designed to measure implementation strength and to compare outlets that are designated as PPM and non-PPM.

This outlet survey was also not explicitly designed to evaluate the malaria CHW programme. CHWs in selected clusters were screened to assess availability of malaria testing and treatment regardless of reported malaria case management training. While most CHWs approached were either Village Malaria Workers or Village Health Volunteers trained and equipped for malaria test and treat services, it is feasible that some Village Health Volunteers screened were not part of the malaria programme. This may have artificially increased the total number of CHWs included in the denominator therefore deflating estimates of the indicator showing the availability of any anti-malarial, among all screened CHWs.

The most senior provider was interviewed at each outlet for this survey. Senior-most providers were interviewed as they are generally in the best position to provide the most accurate reports of price, sales, availability, stock outs and service readiness. Some bias could have been introduced in that key indicators on provider knowledge only reflect answers from these better trained providers. Therefore, knowledge of first-line treatment and appropriate regimen may have been lower if lower-level providers were interviewed for this survey.

While all of the ACT that was audited in the Lao PDR was quality-assured, it should be acknowledged that this quality-assurance status granted by regulatory authorities does not necessarily preclude manufacturing quality failures or prevent conditions or practices that may lead to drug degradation over time. Moreover, anti-malarial treatments that have not been granted pre-qualification status or regulatory approval may be safe and efficacious. Nonetheless, quality-assurance status has been associated with high quality medicines in field drug quality studies [22]. Further research in the Lao PDR is necessary to address the quality of anti-malarial treatments and complement previous evidence on this topic [13].

Finally, while the current outlet survey provided supply side data on the anti-malarial and diagnostic markets of the southern Lao PDR, further information is merited to understand the demand side which this outlet survey did not investigate. A malaria indicator survey implemented in the Lao PDR would provide important and complementary evidence to the data presented herein.

## Conclusions

Public sector outlets in the southern Lao PDR are typically equipped to test and appropriately treat malaria according to national treatment guidelines. However, the private sector is responsible for the majority of anti-malarial distribution. As such there is need to address the widespread private sector availability and distribution of the non-first-line drug, chloroquine. Evidence suggests that the PPM programme approach has been successful in introducing first-line ACT and RDTs and improving readiness of private providers to manage malaria according to national guidelines. However, despite provision of training, supervision and key commodities, private providers continue to stock and distribute chloroquine. Supporting interventions to address provider and consumer behaviours are needed to drive uptake of first-line treatment.

## Additional files

**Additional file 1.** The Lao PDR ACTwatch Survey Questionnaire (English).

**Additional file 2.** The Lao PDR ACTwatch Survey Questionnaire (Lao).

**Additional file 3.** Detailed sample description.

**Additional file 4.** Provider perceptions regarding the most effective treatment for uncomplicated malaria.

**Additional file 5.** Maraquine^®^, a locally manufactured and popular anti-malarial.

## Abbreviations

ACT: artemisinin-based combination therapy
AETD: adult equivalent treatment dose
AL: artemether–lumefantrine
CMPE: Centre for Malaria Parasitology and Entomology
CHW: Community Health Worker
EMA: European Medicines Agency
GPS: Global Positioning System
GMS: Greater Mekong Sub-region
G6PD: glucose-6-phosphate dehydrogenase
LSHTM: London School of Hygiene and Tropical Medicine
RDT: rapid diagnostic test
PPM: public private sector mix
PPS: probability proportional to size
PSI: Population Services International
WHO: World Health Organization
