# Supplementary material for: The malaria testing and treatment landscape in the southern Lao People’s Democratic Republic (PDR)
Source: Malar J. 2017 Apr 25;16:169. doi: 10.1186/s12936-017-1769-0 (PMC5404290; doi:10.1186/s12936-017-1769-0)
Supplement: Supplementary file 1 — Additional file 1. The Lao PDR ACTwatch Survey Questionnaire (English). [file 12936_2017_1769_MOESM1_ESM.docx]

Additional File 1: ACTwatch Questionnaire in English

| **Section 1: Census Information** | | | | |
| --- | --- | --- | --- | --- |
| ***Interviewer completes this section for all outlets.*** | | | | |
| **Outlet ID: Interviewer-District-Village Group-Outlet ID** [___\|___]-[___\|___]-[___\|___\|___]--[___\|___\|___] | | | | |
| C1. Today’s date(dd/mm/yyyy) | | | [___\|___]-[___\|___]-[_2_\|_0_\|_1_\|_5_] | |
| C2. Interviewer’s name [_______________________________________________] | | | C2a. Interviewer’s code [___\|___] | |
| C3. District name [___________________________________________________] | | | C3a. District code [___\|___] | |
| C4. Village group name [_________________________________________________] | | | C4a. Village group code [___\|___\|___] | |
| C5. Village name [__________________________________________________________] | | | | |
| C6. Name of outlet ***If no name, record “no name” or owner’s name***  [__________________________________________________________________] | | | C6a. Outlet code [___\|___\|___] | |
| C7. Type of Outlet  01 Provincial Hospital  02 District Hospital  03 Health Center  04 Village Health Worker (CMPE trained)  05 Military/Police Hospital  07 Mission/NGO Clinic | 08 Private Hospital  09 Private Clinic  10 Clinical Pharmacy  11 Level 1 Pharmacy  12 Level 2 Pharmacy  13 Level 3 Pharmacy  14 Drug store | 15 Village Shop/ Grocery Store  16 Mobile Vendor  17 Private diagnostic laboratory  18 NGO/Mission diagnostic laboratory  19 Village Health Worker (NGO trained)  96 Other ***(specify)***  [_______________________________] | | [___\|___] |
| C8. Is this village group part of the booster sample? 1 = Yes 0=No | | | | [___] |

Hello, my name is __________, I work on behalf of Population Services International. We are conducting a study on the availability of antimalarial medicines and diagnostic testing services. The results will be used to improve the availability of appropriate antimalarial treatment in Lao PDR. I would like to ask you a few questions to see if you could be part of the survey.

**Section 2: Screening & Eligibility**

| S1. Do you have any medicines in stock today?  1 = Yes ***Go to S3***  0 = No | [___] |
| --- | --- |
| S2. Are there any medicines that are out of stock today, but that you stocked in the **past 3 months?**  1 = Yes ***Go to S4***  0 = No ***Go to S5***  8 = Don’t know ***Go to S5*** | [___] |
| S3. Do you have any antimalarial medicines in stock today?  1 = Yes ***Provide information sheet & gain consent. Record start time in C9.***  ***Proceed to Section 3: Antimalarial Audit.***  0 = No Verify with prompt card. ***Go to S4*** | [___] |
| S4. Are there any antimalarial medicines that are out of stock today, but that you stocked in the **past 3 months?**  1 = Yes ***Provide information sheet & gain consent. Record start time in C9. Proceed to A17.***  0 = No ***Verify with prompt card. Go to S5***  8 = Don’t know ***Verify with prompt card. Go to S5*** | [___] |
| S5. Are you offering any diagnostic services or selling any diagnostic tests here today?  1 = Yes ***Go to S6***  0 = No ***Verify with prompt card. Record details in C9 then complete Section X: Ending Interview*** | [___] |
| S6. Are any of these services or tests for suspected malaria?  1 = Yes ***Provide information sheet & gain consent. Record start time in C9.  Proceed to Section 4: Diagnostic Audit***  0 = No ***Verify with prompt card. Record details in C9 then complete Sec X: Ending Interview*** | [___] |

***Before proceeding to the full interview, ensure you have given the respondent a study information sheet, explained the study, and obtained informed consent.***

C9. Result of Visit(s)

| Date  (dd/mm/yy) | **Visit1** | **Visit 2** | **Visit 3** | |
| --- | --- | --- | --- | --- |
|  | [___\|___]-[___\|___]-[_1_\|_5_] | [___\|___]-[___\|___]-[_1_\|_5_] | [___\|___]-[___\|___]-[_1_\|_5_] | |
| Time started ***(in 24hr clock)*** | [___\|___]:[___\|___] | [___\|___]:[___\|___] | [___\|___]:[___\|___] | |
| Time completed ***(in 24hr clock)*** | [___\|___]:[___\|___] | [___\|___]:[___\|___] | [___\|___]:[___\|___] | |
| Result | [___\|___] | [___\|___] | [___\|___] | |
|  | 01 = Outlet eligible & survey completed ***go to E1*** | | | |
|  | 02 = Outlet ineligible: does not meet any screening criteria ***go to E1*** | | | |
|  | 03 = Interview interrupted **go to C11** | | | |
|  | 04 = Respondent not available/time not convenient **go to C11** | | | |
|  | 05 = Outlet not open at the time **go to C11** | | | |
|  | 06 = Outlet closed permanently **go to E1** | | | |
|  | 96 = Other **(specify)**:[_________________________________________________________] | | | |
|  | 97 = Refused **go to C10** | | | |
| C10. If the provider refused, why?  1 = Client load ***Ask respondent for a time they would prefer to be interviewed and note in C11***  2 = Thinks it’s an inspection/nervous about license ***go to E1***  3 = Not interested ***go to E1***  6 = Other (specify):[___________________________________________________________________]  7 = Refuses to give reason ***go to E1*** | | | | [___] |
| C11. ***Use this space to record call back details. If it is not possible to complete the interview at another time, go to E1.*** | | | | |

| **Section X: Ending the interview** | |
| --- | --- |
| E1. Name of interviewee:  [________________________________________________________]  *5 = Not applicable, no respondent; 7 = Refused* | |
| E2. Physical address or location identifiers of outlet (not PO box) ***(Give detailed description that will help supervisor to find the outlet)*** | E3. Telephone number  _________________________________________  *99999999995 = N/A: no respondent or has no telephone*  *99999999997 = Refused*  ***If the telephone number is less than 11 digits, then include leading zeros.*** |
| E4.Latitude:  [_**N**_] - **[___\|___] .** [___\|___\|___\|___\|___\|___] | E5.Longitude:  [_**E**_] - **[___\|___\|___] .** [___\|___\|___\|___\|___\|___] |
| E7. **Additional observations by interviewer (if any)** | |

***THANK THE PROVIDER AND END THE INTERVIEW***

| **Section 3: Antimalarial Audit** |
| --- |

| ***A0. Read to the provider:***  Can you please show us the full range of antimalarials that you currently have in stock? Do you currently have any of the following?  ***Prompt entire list using antimalarial prompt card; No response to be recorded.***   1. Artemether–Lumefantrim (Artefan, *Coartem, Combiart*) 2. Chloroquine Phosphate (Chloroquine Phosphate, *Malacin, Maraquine, Nitaquin*) 3. Chloroquine (*Chloroquine, Nivaquine*) 4. Artesunate (*Artesunate, Artesun, Artesunat*)   ***If the outlet has no antimalarials in stock, cross check screening results then proceed to question A17.*** |
| --- |

***Proceed to the antimalarial audit. Different antimalarial audit sheets will be used to record the antimalarial information based on the dosage form of the medicine.***

***Separate the antimalarials into two piles:***

- ***The first pile should contain all the antimalarials in the form of tablets, suppositories, or granules.
  Use the Tablets, Suppositories & Granules Drug Audit Sheet to record these.***
- ***The second pile should contain all the antimalarials in any form other than tablets, suppositories or granules. Use the Non-Tablet Drug Audit Sheet to record these.***

***If additional audit sheets are used, add these sheets after the ones provided and staple the questionnaire again.***

***All pages should be in order before you move onto the next outlet.***

***Number each drug by assigning a Product Number (starting from 1 for TSG drugs and again from 1 for NT drugs).***

***Number each audit sheet used in the spaces provided at the bottom of the page.***

***ADDITIONAL NOTES ON THE SUB-OUTLET CODE***

***In all outlets, complete the Sub-Outlet Code (as well as the Product Number) for each drug audited. These codes are listed below.***

| ***SUB-OUTLET CODES*** | |
| --- | --- |
| X | ALL outlets that have only ONE dispensing/distribution point for medicines/diagnostics |
| A | Outpatient department / dispensary/Main pharmacy (if used by all patients) |
| B | Adult outpatient department / adult dispensary / adult clinic |
| C | Child outpatient department / child dispensary / child clinic |
| D | Antenatal / maternity clinic/MCH |
| E | ART / HIV/AIDS clinic |
| F | Infectious Disease Department |
| G | Emergency Unit |
| H | Private dispensing unit within a public health facility |
| I | Laboratory ***(for RDT audit)*** |
| Z | Other ***(specify the type in the space for audit comments – TSG 15 or NT 15)*** |

| **Sub-outlet code**  [_____]  **Product number**  [__\|__] | [__\|__]  [__\|__]  [__\|__] | **1. Generic name** | | | | **2. Strength**  [__\|__\|__].[__]mg  [__\|__\|__].[__]mg  [__\|__\|__].[__]mg | | | **2a. Is this base strength?**  [__]  1 = Yes  [__] 0 = No  8 = Don’t know  [__]  ***If no, specify salt:***  [________________________] | | | **3. Dosage form/formulation**  1 = Tablet  2 = Suppository  3 = Granule  [___] | | **4. Brand name**  *(Include weight and age information)* | | |
| --- | --- | --- | --- | --- | --- | --- | --- | --- | --- | --- | --- | --- | --- | --- | --- | --- |
|  | [__\|__] | | | | |  |  |  |  |  |  |  |  |  |  |  |
| **5. Manufacturer** | | | **6. Country of manufacture** | | **7. Package size**  There are a total of  [___\|___\|___\|___] tablets/ suppositories/ granule sachets in each:  1 = Package  2 = Pot/tin  [___] | | **8. Is product a fixed-dose combination (FDC)**  1 = Yes  0 = No  8 = Don’t   know  [___] | | | **9. Does product have the Green leaf logo?**  1 = Yes  0 = No  8 = Don’t   know  [___] | **10. Amount sold/distributed in the last 7 days to individual consumers** (*Record # of packages / tins described in* Q7 *OR record the total # of tablets / suppositories / granule packs sold*)  This outlet sold [___\|___\|___] **packages/ tins** in the last 7 days    **OR**  This outlet sold [___\|___\|___] **tablets/ suppositories or granule sachets** in the last 7 days  ***Not applicable = 995; Refused = 997; Don’t know = 998*** | | | | | **11. Stocked out at any point in the past 3 months?**  1 = Yes  0 = No  8 = Don’t   know  [___] |
|  |  |  | [__\|__\|__] | |  |  |  |  |  |  |  |  |  |  |  |  |
| **12. Retail selling price**  [___\|___\|___]  **tablets, suppositories or granule sachets** cost an individual customer  [___\|___\|___\|___\|___]___] Kip | | | | **13. Wholesale purchase price**  For the outlet’s most recent wholesale purchase  [___\|___\|___\|___]  **tablets, suppositories or granule sachets** cost  [___\|___\|___\|___\|___\|___\|___] Kip | | | | **14. Why do you stock this medicine [SHOW PRODUCT]?**  ***Do not read list***.  ***Circle ALL responses given***  Free supply A  Profitable B  Recommended by the government C  Low price D  Customer demand or preference E  Positive brand reputation F  Often prescribed by doctors G  Most effective for treating malaria H  Don’t know X  Other Z  ***specify*** [_________________________] | | | | | **15. Comments** | | **16a. Is this medicine a tablet?**   \| 1 = Yes 0 = No \| [___] \| \| --- \| --- \|   ***If No then continue to the next audit sheet.***  **16b. Does this medicine have only 1 active ingredient?**   \| 1 = Yes 0 = No \| [___] \| \| --- \| --- \|   ***If No then continue to the next audit sheet.***  **16c. Is the active ingredient one of the following?**  ARTEMETHER  ARTESUNATE  DIHYDROARTEMISININ   \| 1 = Yes 0 = No \| [___] \| \| --- \| --- \|   ***If Yes then complete oral AMT module after "Antimalarial stock out section" completed.*** | |
| ***Free = 000000***  ***Refused = 999997 Don’t know = 999998*** | | | | ***Free = 0000000***  ***Refused = 9999997***  ***Don’t know = 9999998*** | | | |  |  |  |  |  |  |  |  |  |

TSG Audit Sheet [___|___] of [___|___]

| **Sub-outlet code**  **[_____]**  **Product number**  **[__\|__]** | [__\|__]  [__\|__]  [__\|__] | **1. Generic name** | | | | **2. Strength**  **[__\|__\|__\|__].[__]mg/[__\|__\|__] .[__]mL**  **[__\|__\|__\|__].[__]mg/[__\|__\|__] .[__]mL**  **[__\|__\|__\|__].[__]mg/[__\|__\|__] .[__]mL**  **(Note: no mL recorded for powder injection)** | | | | **2a. Is this base strength?**  **[__]**  **1 = Yes**  **[__] 0 = No**  **8 = Don’t know**  **[__]**  **If no, specify salt:**  **[______________________]** | | | **3. Dosage form/formulation**  **1 = Syrup**  **2 = Suspension**  **3 = Liquid injection**  **4 = Powder injection**  **5 = Drops**  **6 = Other (specify) [___________]**  **[___]** | |
| --- | --- | --- | --- | --- | --- | --- | --- | --- | --- | --- | --- | --- | --- | --- |
|  | [__\|__] | | | | |  |  |  |  |  |  |  |  |  |
| **4. Brand name**  *(Include weight and age information)* | | | **5. Manufacturer** | | **6. Country of manufacture** | | | **7. Package size**  There are a total of  [___\|___\|___\|___].[___] mL  (or mg for powder injections) in each:  1 = Bottle  2 = Ampoule/vial  [___] | **9. Does this product have the Green leaf logo?**  1 = Yes  0 = No  8 = Don’t   know  [___] | | **10. Amount sold/ distributed in the last 7 days to individual consumers**  This outlet sold  [___\|___\|___\|___]  **bottles, ampoules or vials** in the  last 7 days  ***Refused = 9997;***  ***Don’t know = 9998*** | | | **11. Stocked out at any point in the past 3 months?**  1 = Yes  0 = No  8 = Don’t   know  [___] |
|  |  |  |  |  | [__\|__\|__] | | |  |  |  |  |  |  |  |
| **12. Retail selling price**  [___\|___\|___]  **bottles ampoules or vials** cost an individual customer  [___\|___\|___\|___\|___]___] Kip | | | | **13. Wholesale purchase price**  For the outlet’s most recent wholesale purchase:  [___\|___\|___\|___]  **bottles, ampoules or vials** cost  [___\|___\|___\|___\|___\|___\|___] Kip | | | **14. Why do you stock this medicine [SHOW PRODUCT]? *Do not read list. Circle ALL responses given.***  Free supply A  Profitable B  Recommended by the government C  Low price D  Customer demand or preference E  Positive brand reputation F  Often prescribed by doctors G  Most effective for treating malaria H  Don’t know X  Other Z  ***specify*** [_________________________] | | | | | **15. Comments** | | |
| ***Free = 000000***  ***Refused = 999997***  ***Don’t know = 999998*** | | | | ***Free = 0000000***  ***Refused = 9999997***  ***Don’t know = 9999998*** | | |  |  |  |  |  |  |  |  |

Non-Tablet Audit Sheet [___|___] of [___|___]

| A16_1. Do you have any antibiotics in stock today?  1 = Yes  0 = No **Go to A17** | [___] |
| --- | --- |
| A16_2. Do you have any doxycycline tablets/capsules in stock today?  ***Show prompt card. Interviewer: ask to see the product and verify that it is doxycycline.***  1 = Yes  0 = No          ***Go to A17*** | [___] |
| A16_3. Which strength of doxycycline tablets/capsules do you have in stock today?  ***Read list, circle ALL that apply*** |  |
| 100 mg | A |
| Don't know | X |
| Other (specify): [________________________________________] | Z |

**Antimalarial stock outs**

| A17. Are there any antimalarial medicines that are out of stock today, but that you stocked in the  past **3 months?**  1 = Yes ***go to A18***  0 = No ***go to A20***  8 = Don’t know ***go to A20*** | [___] |
| --- | --- |
| A18.Do you know the names of the antimalarial medicines that are out of stock?  1 = Yes  0 = No, provider can’t remember ***Go to A20*** | [___] |

| A17a. What are the names and formulations of the antimalarial medicines that are out of stock?  ***Refer to the following list of brand names when completing question #4 below.***  **Brand Name:**   \| ***Artesunate*** \|  \| ***Artemether*** \| ***Dihydroartemisinin*** \| \| --- \| --- \| --- \| --- \| \| ***AA-Artesunate (tab)*** \| ***Falcinate*** \| ***Artem*** \| ***Alaxin*** \| \| ***Artesunate (tab)*** \| ***Gricin*** \| ***AA-Artemether*** \| ***Codisin*** \| \| ***ArthesisArinate*** \| ***Gsunate*** \| ***Armether*** \| ***Temecxin*** \| \| ***Actitesunate*** \| ***Lever-Artesunate*** \| ***Artemedine*** \| ***Santecxin*** \| \| ***Adamsnate*** \| ***MD-Artesunate*** \| ***Betamotil*** \|  \| \| ***Artemed*** \| ***Pedisunate*** \|  \|  \| \| ***Askasunate*** \| ***Traphasunat*** \|  \|  \| \| ***Cusnat-Artesunate*** \| ***Vatunate*** \|  \|  \| \| ***Artesunat*** \|  \|  \|  \| \| ***Eurosunate*** \|  \|  \|  \|  \| **Stock out number**  [___\|___] \| **1. Generic or brand name**  [____________________]  98 = Don't know \| ***Do not ask provider.***  **4. Does this medicine have a brand name included in the list of oral AMT brands?**   \| 1 = Yes 0 = No 8 = Don't know \| [___] \| \| --- \| --- \|   ***If Yes then complete oral AMT module after ‘Antimalarial Stock out section’ is completed.***  ***If No go to 5.***  **5. Is this medicine a tablet?**   \| 1 = Yes 0 = No 8 = Don't know \| [___] \| \| --- \| --- \|   ***If No or Don't know then continue to the next medicine.*** \| **6. Does this medicine have only 1 active ingredient?**   \| 1 = Yes 0 = No 8 = Don't know \| [___] \| \| --- \| --- \|   ***If No or Don't know then continue to the next medicine.***  **7. Is the active ingredient one of the following?**  ARTEMETHER  ARTESUNATE  DIHYDROARTEMISININ   \| 1 = Yes 0 = No \| [___] \| \| --- \| --- \|   ***If Yes then complete oral AMT module after "Antimalarial stock out section" completed.*** \| \| --- \| --- \| --- \| --- \| --- \| --- \| --- \| --- \| --- \| --- \| --- \| --- \| \| **2. Dosage form/formulation**  01 = Tablet 05 = Suspension  02 = Suppository 06 = IM/IV Injection  03 = Granule (liquid or powder)  04 = Syrup 07 = Drops  98 = Don't know  [___\|___] \| \| \| **3. Do you plan to purchase more of this medicine?**  1 = Yes  0 = No [___]  8 = Don't know \| \|  \| **Stock out number**  [___\|___] \| **1. Generic or brand name**  [____________________]  98 = Don't know \| ***Do not ask provider.***  **4. Does this medicine have a brand name included in the list of oral AMT brands?**   \| 1 = Yes 0 = No 8 = Don't know \| [___] \| \| --- \| --- \|   ***If Yes then complete oral AMT module after ‘Antimalarial Stock out section’ is completed.***  ***If No go to 5.***  **5. Is this medicine a tablet?**   \| 1 = Yes 0 = No 8 = Don't know \| [___] \| \| --- \| --- \|   ***If No or Don't know then continue to the next medicine.*** \| **6. Does this medicine have only 1 active ingredient?**   \| 1 = Yes 0 = No 8 = Don't know \| [___] \| \| --- \| --- \|   ***If No or Don't know then continue to the next medicine.***  **7. Is the active ingredient one of the following?**  ARTEMETHER  ARTESUNATE  DIHYDROARTEMISININ   \| 1 = Yes 0 = No \| [___] \| \| --- \| --- \|   ***If Yes then complete oral AMT module after "Antimalarial stock out section" completed.*** \| \| --- \| --- \| --- \| --- \| --- \| --- \| --- \| --- \| --- \| --- \| --- \| --- \| \| **2. Dosage form/formulation**  01 = Tablet 05 = Suspension  02 = Suppository 06 = IM/IV Injection  03 = Granule (liquid or powder)  04 = Syrup 07 = Drops  98 = Don't know  [___\|___] \| \| \| **3. Do you plan to purchase more of this medicine?**  1 = Yes  0 = No [___]  8 = Don't know \| \|   A17a. What are the names and formulations of the antimalarial medicines that are out of stock?  ***Refer to the following list of brand names when completing question #4 below.***  **Brand Name:**   \| ***Artesunate*** \|  \| ***Artemether*** \| ***Dihydroartemisinin*** \| \| --- \| --- \| --- \| --- \| \| ***AA-Artesunate (tab)*** \| ***Falcinate*** \| ***Artem*** \| ***Alaxin*** \| \| ***Artesunate (tab)*** \| ***Gricin*** \| ***AA-Artemether*** \| ***Codisin*** \| \| ***ArthesisArinate*** \| ***Gsunate*** \| ***Armether*** \| ***Temecxin*** \| \| ***Actitesunate*** \| ***Lever-Artesunate*** \| ***Artemedine*** \| ***Santecxin*** \| \| ***Adamsnate*** \| ***MD-Artesunate*** \| ***Betamotil*** \|  \| \| ***Artemed*** \| ***Pedisunate*** \|  \|  \| \| ***Askasunate*** \| ***Traphasunat*** \|  \|  \| \| ***Cusnat-Artesunate*** \| ***Vatunate*** \|  \|  \| \| ***Artesunat*** \| ***Eurosunate*** \|  \|  \|  \| **Stock out number**  [___\|___] \| **1. Generic or brand name**  [____________________]  98 = Don't know \| ***Do not ask provider.***  **4. Does this medicine have a brand name included in the list of oral AMT brands?**   \| 1 = Yes 0 = No 8 = Don't know \| [___] \| \| --- \| --- \|   ***If Yes then complete oral AMT module after ‘Antimalarial Stock out section’ is completed.***  ***If No go to 5.***  **5. Is this medicine a tablet?**   \| 1 = Yes 0 = No 8 = Don't know \| [___] \| \| --- \| --- \|   ***If No or Don't know then continue to the next medicine.*** \| **6. Does this medicine have only 1 active ingredient?**   \| 1 = Yes 0 = No 8 = Don't know \| [___] \| \| --- \| --- \|   ***If No or Don't know then continue to the next medicine.***  **7. Is the active ingredient one of the following?**  ARTEMETHER  ARTESUNATE  DIHYDROARTEMISININ   \| 1 = Yes 0 = No \| [___] \| \| --- \| --- \|   ***If Yes then complete oral AMT module after "Antimalarial stock out section" completed.*** \| \| --- \| --- \| --- \| --- \| --- \| --- \| --- \| --- \| --- \| --- \| --- \| --- \| \| **2. Dosage form/formulation**  01 = Tablet 05 = Suspension  02 = Suppository 06 = IM/IV Injection  03 = Granule (liquid or powder)  04 = Syrup 07 = Drops  98 = Don't know  [___\|___] \| \| \| **3. Do you plan to purchase more of this medicine?**  1 = Yes  0 = No [___]  8 = Don't know \| \|  \| **Stock out number**  [___\|___] \| **1. Generic or brand name**  [____________________]  98 = Don't know \| ***Do not ask provider.***  **4. Does this medicine have a brand name included in the list of oral AMT brands?**   \| 1 = Yes 0 = No 8 = Don't know \| [___] \| \| --- \| --- \|   ***If Yes then complete oral AMT module after ‘Antimalarial Stock out section’ is completed.***  ***If No go to 5.***  **5. Is this medicine a tablet?**   \| 1 = Yes 0 = No 8 = Don't know \| [___] \| \| --- \| --- \|   ***If No or Don't know then continue to the next medicine.*** \| **6. Does this medicine have only 1 active ingredient?**   \| 1 = Yes 0 = No 8 = Don't know \| [___] \| \| --- \| --- \|   ***If No or Don't know then continue to the next medicine.***  **7. Is the active ingredient one of the following?**  ARTEMETHER  ARTESUNATE  DIHYDROARTEMISININ   \| 1 = Yes 0 = No \| [___] \| \| --- \| --- \|   ***If Yes then complete oral AMT module after "Antimalarial stock out section" completed.*** \| \| --- \| --- \| --- \| --- \| --- \| --- \| --- \| --- \| --- \| --- \| --- \| --- \| \| **2. Dosage form/formulation**  01 = Tablet 05 = Suspension  02 = Suppository 06 = IM/IV Injection  03 = Granule (liquid or powder)  04 = Syrup 07 = Drops  98 = Don't know  [___\|___] \| \| \| **3. Do you plan to purchase more of this medicine?**  1 = Yes  0 = No [___]  8 = Don't know \| \|  \| **Stockout number**  [___\|___] \| **1. Generic or brand name**  [____________________]  98 = Don't know \| ***Do not ask provider.***  **4. Does this medicine have a brand name included in the list of oral AMT brands?**   \| 1 = Yes 0 = No 8 = Don't know \| [___] \| \| --- \| --- \|   ***If Yes then complete oral AMT module after ‘Antimalarial Stock out section’ is completed.***  ***If No go to 5.***  **5. Is this medicine a tablet?**   \| 1 = Yes 0 = No 8 = Don't know \| [___] \| \| --- \| --- \|   ***If No or Don't know then continue to the next medicine.*** \| **6. Does this medicine have only 1 active ingredient?**   \| 1 = Yes 0 = No 8 = Don't know \| [___] \| \| --- \| --- \|   ***If No or Don't know then continue to the next medicine.***  **7. Is the active ingredient one of the following?**  ARTEMETHER  ARTESUNATE  DIHYDROARTEMISININ   \| 1 = Yes 0 = No \| [___] \| \| --- \| --- \|   ***If Yes then complete oral AMT module after "Antimalarial stock out section" completed.*** \| \| --- \| --- \| --- \| --- \| --- \| --- \| --- \| --- \| --- \| --- \| --- \| --- \| \| **2. Dosage form/formulation**  01 = Tablet 05 = Suspension  02 = Suppository 06 = IM/IV Injection  03 = Granule (liquid or powder)  04 = Syrup 07 = Drops  98 = Don't know  [___\|___] \| \| \| **3. Do you plan to purchase more of this medicine?**  1 = Yes  0 = No [___]  8 = Don't know \| \|   A17a. What are the names and formulations of the antimalarial medicines that are out of stock?  ***Refer to the following list of brand names when completing question #4 below.***  **Brand Name:**   \| ***Artesunate*** \|  \| ***Artemether*** \| ***Dihydroartemisinin*** \| \| --- \| --- \| --- \| --- \| \| ***AA-Artesunate (tab)*** \| ***Falcinate*** \| ***Artem*** \| ***Alaxin*** \| \| ***Artesunate (tab)*** \| ***Gricin*** \| ***AA-Artemether*** \| ***Codisin*** \| \| ***ArthesisArinate*** \| ***Gsunate*** \| ***Armether*** \| ***Temecxin*** \| \| ***Actitesunate*** \| ***Lever-Artesunate*** \| ***Artemedine*** \| ***Santecxin*** \| \| ***Adamsnate*** \| ***MD-Artesunate*** \| ***Betamotil*** \|  \| \| ***Artemed*** \| ***Pedisunate*** \|  \|  \| \| ***Askasunate*** \| ***Traphasunat*** \|  \|  \| \| ***Cusnat-Artesunate*** \| ***Vatunate*** \|  \|  \| \| ***Artesunat*** \| ***Eurosunate*** \|  \|  \|  \| **Stock out number**  [___\|___] \| **1. Generic or brand name**  [____________________]  98 = Don't know \| ***Do not ask provider.***  **4. Does this medicine have a brand name included in the list of oral AMT brands?**   \| 1 = Yes 0 = No 8 = Don't know \| [___] \| \| --- \| --- \|   ***If Yes then complete oral AMT module after ‘Antimalarial Stock out section’ is completed.***  ***If No go to 5.***  **5. Is this medicine a tablet?**   \| 1 = Yes 0 = No 8 = Don't know \| [___] \| \| --- \| --- \|   ***If No or Don't know then continue to the next medicine.*** \| **6. Does this medicine have only 1 active ingredient?**   \| 1 = Yes 0 = No 8 = Don't know \| [___] \| \| --- \| --- \|   ***If No or Don't know then continue to the next medicine.***  **7. Is the active ingredient one of the following?**  ARTEMETHER  ARTESUNATE  DIHYDROARTEMISININ   \| 1 = Yes 0 = No \| [___] \| \| --- \| --- \|   ***If Yes then complete oral AMT module after "Antimalarial stock out section" completed.*** \| \| --- \| --- \| --- \| --- \| --- \| --- \| --- \| --- \| --- \| --- \| --- \| --- \| \| **2. Dosage form/formulation**  01 = Tablet 05 = Suspension  02 = Suppository 06 = IM/IV Injection  03 = Granule (liquid or powder)  04 = Syrup 07 = Drops  98 = Don't know  [___\|___] \| \| \| **3. Do you plan to purchase more of this medicine?**  1 = Yes  0 = No [___]  8 = Don't know \| \|  \| **Stock out number**  [___\|___] \| **1. Generic or brand name**  [____________________]  98 = Don't know \| ***Do not ask provider.***  **4. Does this medicine have a brand name included in the list of oral AMT brands?**   \| 1 = Yes 0 = No 8 = Don't know \| [___] \| \| --- \| --- \|   ***If Yes then complete oral AMT module after ‘Antimalarial Stock out section’ is completed.***  ***If No go to 5.***  **5. Is this medicine a tablet?**   \| 1 = Yes 0 = No 8 = Don't know \| [___] \| \| --- \| --- \|   ***If No or Don't know then continue to the next medicine.*** \| **6. Does this medicine have only 1 active ingredient?**   \| 1 = Yes 0 = No 8 = Don't know \| [___] \| \| --- \| --- \|   ***If No or Don't know then continue to the next medicine.***  **7. Is the active ingredient one of the following?**  ARTEMETHER  ARTESUNATE  DIHYDROARTEMISININ   \| 1 = Yes 0 = No \| [___] \| \| --- \| --- \|   ***If Yes then complete oral AMT module after "Antimalarial stock out section" completed.*** \| \| --- \| --- \| --- \| --- \| --- \| --- \| --- \| --- \| --- \| --- \| --- \| --- \| \| **2. Dosage form/formulation**  01 = Tablet 05 = Suspension  02 = Suppository 06 = IM/IV Injection  03 = Granule (liquid or powder)  04 = Syrup 07 = Drops  98 = Don't know  [___\|___] \| \| \| **3. Do you plan to purchase more of this medicine?**  1 = Yes  0 = No [___]  8 = Don't know \| \| |
| --- | --- | --- | --- | --- | --- | --- | --- | --- | --- | --- | --- | --- | --- | --- | --- | --- | --- | --- | --- | --- | --- | --- | --- | --- | --- | --- | --- | --- | --- | --- | --- | --- | --- | --- | --- | --- | --- | --- | --- | --- | --- | --- | --- | --- | --- | --- | --- | --- | --- | --- | --- | --- | --- | --- | --- | --- | --- | --- | --- | --- | --- | --- | --- | --- | --- | --- | --- | --- | --- | --- | --- | --- | --- | --- | --- | --- | --- | --- | --- | --- | --- | --- | --- | --- | --- | --- | --- | --- | --- | --- | --- | --- | --- | --- | --- | --- | --- | --- | --- | --- | --- | --- | --- | --- | --- | --- | --- | --- | --- | --- | --- | --- | --- | --- | --- | --- | --- | --- | --- | --- | --- | --- | --- | --- | --- | --- | --- | --- | --- | --- | --- | --- | --- | --- | --- | --- | --- | --- | --- | --- | --- | --- | --- | --- | --- | --- | --- | --- | --- | --- | --- | --- | --- | --- | --- | --- | --- | --- | --- | --- | --- | --- | --- | --- | --- | --- | --- | --- | --- | --- | --- | --- | --- | --- | --- | --- | --- | --- | --- | --- | --- | --- | --- | --- | --- | --- | --- | --- | --- | --- | --- | --- | --- | --- | --- | --- | --- | --- | --- | --- | --- | --- | --- | --- | --- | --- | --- | --- | --- | --- | --- | --- | --- | --- | --- | --- | --- | --- | --- | --- | --- | --- | --- | --- | --- | --- | --- | --- | --- | --- | --- | --- | --- | --- | --- | --- |

| A20. ***Don't read***: Was oral AMT identified during the antimalarial audit or in question A17a?  1 = Yes ***Complete the oral AMT module for each oral AMT identified.***  ***An oral AMT module should be completed for:***  ***1) All audited TSG products with responses of 1 for questions 16a, 16b and 16c***  ***2) All currently out of stock products with responses of 1 for question A17a, #4 and #7.***  0 = No ***Proceed to Section 4: Diagnostic Audit.*** | [___] |
| --- | --- |

| **Section 4: Diagnostic Audit**  *This section is about availability of malaria blood testing. Completing the questions may require speaking with more than 1 staff member at the outlet. If the respondent does not know the answer to a question in this section, ask to speak with another staff member who can provide the information.* |
| --- |

| D1. Does this outlet/facility have disposable gloves available today for staff to use when seeing customers/patients?  1 = Yes  0 = No  8 = Don’t know | [___] |
| --- | --- |
| D2. Does this outlet/facility have a sharps container, also called a sharps disposal box or safety box, available today for staff to use?  1 = Yes  0 = No  8 = Don’t know | [___] |
| D3.Is malaria microscopic testing available here today?  1= Yes  0= No **g*o to D6a*** | [___] |
| D4. How many people were tested for **malaria** at this facility/outlet **using microscopy** within the past 7 days?  **997 = Refused; 998 = Don’t know** | [___\|___\|___] |
| D5.  What is the total cost for a microscopic test for malaria for an adult: [___\|___\|___\|___\|___]___] Kip  ***Free = 000000; NA =999995; Refused = 999997; Don’t know=999998*** | |
| D6a. G6PD tests are used to determine if an individual has a G6PD deficiency. Specific types of malaria medicines can harm patients with G6PD deficiency.  **Have you ever heard of G6PD tests?**  1 = Yes  0 = No ***go to d7*** | [___] |
| D6b. Does this outlet test people for G6PD deficiency?  1 = Yes  0 = No ***go to d7***  Don’t know ***ask to speak with a respondent who has this information*** | [___] |
| D6c. Does this outlet use rapid diagnostic tests (RDTs) for G6PD deficiency testing?  1 = Yes  0 = No ***go to d7***  Don’t know ***ask to speak with a respondent who has this information*** | [___] |
| D6d. Which G6PD rapid diagnostic tests (RDTs) are used in this outlet?  ***Ask the provider to gather the G6PD rapid diagnostic tests.***  ***Do not read list. Circle ALL G6PD tests observed to be in stock.***  ***Record brand name and manufacturer for any observed G6PD RDTs not listed below.*** |  |
| CareStart™ G6PD RDT manufactured by Access Bio | A |
| BinaxNOW G6PD RDT manufactured by Alere | B |
| G-6-PDH Dye Reduction RDT manufactured by Trinity Biotech | C |
| Other (specify brand name and manufacturer) [_______________________________________] | X |
| D6e. How many people has this outlet tested for G6PD deficiency using an RDT in the past month?  ***997 = refused, 998 = don't know*** | [___\|___\|___] |
| D7. Malaria rapid diagnostic tests, also called RDTs, are small, individually wrapped blood tests that are able to quickly diagnose whether a person has malaria. ***Show RDT images in prompt card***  Are malaria RDTs available here today?  1 = Yes  0 = No  ***go to D9***  Don’t know ***ask to speak with a respondent who has this information*** | [___] |
| D8. Please show us the full range of RDTs that you currently have in stock. Do you currently have any of the following?  ***Read entire list; No response to be recorded.***   - *Accurate, CareStart™, Clungene, First Response, Malacheck, One Step, ParaHit, Paracheck, SD Bioloine, Humasis, Dialab, Combo-RDT* | |

***Proceed to the RDT audit.***

***If additional audit sheets are used, add these sheets after the ones provided and staple the questionnaire again. All pages should be in order before you move onto the next outlet.***

***Number each RDT by assigning a Product Number.***

***Number each audit sheet used in the spaces provided at the bottom of the page.***

***In all outlets, complete the Sub-Outlet Code (as well as the Product Number) for each drug audited. These codes are listed below.***

| ***SUB-OUTLET CODES*** | |
| --- | --- |
| X | ALL outlets that have only ONE dispensing/distribution point for medicines/diagnostics |
| A | Outpatient department / dispensary / Main pharmacy (if used by all patients) |
| B | Adult outpatient department / adult dispensary / adult clinic |
| C | Child outpatient department / child dispensary / child clinic |
| D | Antenatal / maternity clinic/MCH |
| E | ART / HIV/AIDS clinic |
| F | Infectious Disease Department |
| G | Emergency Unit |
| H | Private dispensing unit within a public health facility |
| I | Laboratory ***(for RDT audit)*** |
| Z | Other ***(specify the type in the space for audit comments – TSG 15 or NT 15)*** |

| **Sub-outlet code**  [_____]  **Product number**  [__\|__] | **1. Brand name** | | | **2. Antigen test**  *(circle ALL that apply)*  HRP2 **A**  pLDH **B**  Aldolase **C**  Not indicated **Z** | **3. Parasite species**  *(circle ALL that apply)*  Pf **A**  Pv **B**  Po **C**  pm **D**  pan **E**  vom/Pvom **F**  Other **G**  **Specify** [_____________________]  Not indicated **Z** | | **4. Manufacturer** | **5. Country of Manufacture** | | **5b. Product Catalogue Number** | **6. Lot Number** | **7. Is this a self test kit, with each test kit co-packaged with its own buffer, pipette and lancet ?**  1 = Yes  0 = No  8 = Don’t know  [___] | |
| --- | --- | --- | --- | --- | --- | --- | --- | --- | --- | --- | --- | --- | --- |
| **13. Number of tests sold/ distributed /used in the last 7 days to individual consumers**  *(Record total # of tests)*  This outlet sold or distributed  [___\|___\|___\|___] **tests** in the last 7 days  ***Refused = 9997; Don’t know = 9998*** | | **14. Has this test been stocked out at any point in the past 3 months?**  1 = Yes  0 = No  8 = Don’t know  [___] | **15a. Do you or other staff use this brand of RDT to test clients here at this facility/outlet?**  1 = Yes  0 = No ***go to 16a***  8 = Don’t know ***go to 16a***  [___]    **15b. If yes, what is the total cost to have a test conducted with this RDT, including RDT cost and service fee?**  [___\|___\|___\|___\|___]___] Kip | | | **16a. Does this facility/outlet provide this brand of RDT for clients to take away for testing somewhere else?**  1 = Yes  0 = No ***go to 17***  8 = Don’t know ***go to 17***  [___]    **16b. If yes, what is cost of this RDT?**  [___\|___\|___\|___\|___]___] Kip | **17. Wholesale purchase price**  For the outlet’s most recent wholesale purchase:  [___\|___\|___\|___] **tests**  cost  [___\|___\|___\|___\|___\|___\|___] Kip  ***Free = 0000000***  ***NA = 9999995***  ***Refused = 9999997***  ***Don’t know=9999998*** | | **18. Why do you stock this RDT [SHOW RDT]?**  ***Do not read list***  ***Circle ALL responses given***  Free supply A  Profitable B    Recommended by the government C  Low price D  Customer demand or preference E  Positive brand reputation F  Don’t know X  Other Z  ***specify*** [_______________________________] | | | | **19. Comment** |
|  |  |  | ***Free = 000000; NA = 999995; Refused = 999997; Don’t know=999998*** | | | |  |  |  |  |  |  |  |

RDT Audit Sheet [___|___] of [___|___]

**RDT stock outs**

| D9. Are there any malaria RDTs that are out of stock today, but that you stocked in the past **3 months**?  1 = Yes  0 = No ***go to D11***  8 = Don’t know ***go to D11*** | [___] |
| --- | --- |
| D9a. Does the provider know the brand name of the RDT?  1 = Yes  0 = No **go to D11** | [___] |
| D10.What are the brand names of the malaria RDTs that are out of stock?  ***Record one brand per line.***  0 = No ***go to D11***  1 = Yes ***specify***  [____________________________________________________________________________]  [____________________________________________________________________________]  [____________________________________________________________________________]  0 = No, provider can’t remember | [___] |
| D11.Does this facility/outlet **provide medicines or prescription** for medicines?  1 = Yes ***go to Section 5: Provider Module***  0 = No ***Confirm response in S1 or S2 is not equal to 1 and outlet type recorded in C7***  ***is 17 or 18 (“lab only”). Go to Section 6: Audit Tracking Sheet.*** | [___] |

| **Section 5: Provider Module**  ***This section is for the senior-most staff member who is responsible for providing treatment, prescriptions or medicines to clients/patients.*** |
| --- |

| P1. Do your responsibilities at this outlet/facility include: providing prescriptions, treatment, or medicines to clients?  1 = Yes  No ***ask to speak with the senior-most person at the outlet with 1 or more of these***  ***responsibilities.*** | | \|___] |
| --- | --- | --- |
| P2. For how many years have you worked in this outlet/facility? **If less than 1 year, enter 01** | | [___\|___] |
| P3.What age are you today? ***Write age in years***  97 = Refused  98 = Don’t know | [___\|___] | |
| P4.***Don’t read:*** Is respondent male or female?  1 = Male  2= Female | \|___] | |
| P5.What is the highest level of education you completed?  1 = No formal education  2 = Some primary school  3 = Completed primary school  4 = Some secondary school  5 = Completed secondary school  6 = Some university/college/technical  7 = Completed a university/college/technical degree/diploma | [___] | |
| P6. Has anyone in this outlet (including you) received any training in the last 12 months that included a component on malaria diagnosis, including malaria rapid diagnostic tests or microscopy?  ***Include pre-service training and stand-alone workshops.***  1 = Yes  0 = No **go to P7**.  8 = Don’t know **go to P7** | | [___] |
| P6a. What organization provided the training that included a component on malaria diagnosis?  ***Do not read list and circle all that apply.***  ***Prompt “any other organizations” until the respondent is finished.*** | |  |
| 1. Government/MOH / CMPE (PPM) | | A |
| 1. World Health Organization (WHO) | | B |
| 1. Health Poverty Action (HPA) | | C |
| 1. Other, specify [__________________________________________________] | | X |
| 1. Don't Know | | Z |
| P7. Has anyone in this outlet (including you) received any training in the last 12 months on the national treatment guidelines for malaria? ***Include pre-service training and stand-alone workshops.***  1 = Yes  0 = No **go to P8**  8 = Don’t know **go to P8** | | [___] |
| P7a. What organization provided the training on the national treatment guidelines for malaria?  ***Do not read list and circle all that apply.***  ***Prompt “any other organizations” until the respondent is finished.*** | |  |
| 1. MOH / CMPE | | A |
| 1. Health Poverty Action (HPA) | | B |
| 1. Other, specify [__________________________________________________] | | X |
| 1. Don't Know | | Z |
| P8. Do you have any of the following **health qualifications**?  ***Read list*.  *Record 1 for yes, 0 for no*** | |  |
| 1. Pharmacist | | [___] |
| 1. Medical Doctor | | [___] |
| 1. Medical Assistant | | [___] |
| 1. Nurse | | [___] |
| 1. Midwife | | [___] |
| 1. Laboratory technician / Lab assistant | | [___] |
| 1. Pharmacy technician / Pharmacy assistant | | [___] |
| 1. Nursing Assistant / Nursing Aid | | [___] |
| 1. Village Health Worker / Village Health Volunteer | | [___] |

| P9. **Not** including yourself, do any other people working in this outlet or facility have the following **health qualifications**? ***Read list***.  ***Record 1 for yes, 0 for no, 8 for don’t know*** |  |
| --- | --- |
| 1. Pharmacist | [___] |
| 1. Medical Doctor | [___] |
| 1. Medical Assistant | [___] |
| 1. Nurse / Nursing Officer | [___] |
| 1. Midwife | [___] |
| 1. Laboratory technician / Lab assistant | [___] |
| 1. Pharmacy technician / Pharmacy assistant | [___] |
| 1. Health Assistant, Nursing Assistant / Nursing Aid | [___] |
| 1. Village Health Worker / Village Health Volunteer | [___] |

| M1. ***Do not read***: Is this outlet a Public Health Facility? Public health facilities include provincial hospitals, district hospitals, health centers or village health workers trained by CMPE. ***Cross-check with question c7.***  1 = Yes ***Go to P10***  0 = No | [___] |
| --- | --- |
| M2. Does this outlet receive free antimalarial treatments or purchase reduced-cost antimalarial treatments from a government or non-governmental organization?  1 = Yes  0 = No ***Go to M4***  8 = Don’t know ***Go to M4*** | [___] |
| M3. From where does this outlet receive free or reduced-cost antimalarial treatments?  ***Do not read list and circle all that apply.*** |  |
| 1. Government / Medical Products Supply Center (MPSC) | A |
| 1. Specific Government / Public Health Facility | B |
| 1. Health Poverty Action (HPA) | C |
| 1. Other, specify [___________________________________________________] | X |
| M4. Does this outlet receive free RDTs or purchase reduced-cost RDTs from a government or non-governmental organization?  1 = Yes  0 = No ***Go to M6***  8 = Don’t know ***Go to M6*** | [___] |
| M5. From where does this outlet receive free or reduced-cost RDTs?  ***Do not read list and circle all that apply.*** |  |
| 1. Government / Medical Products Supply Center (MPSC) | A |
| 1. Specific government / Public Health Facility | B |
| 1. Other, specify [___________________________________________________] | X |

| M6. Has this outlet received a visit within the last year from a government or non-governmental organization that is providing a kind of support, regulation or supervision for the outlet specifically for malaria case management?  1 = Yes  0 = No ***Go to M9***  8 = Don’t know ***Go to M9*** | [___] |
| --- | --- |
| M7. From which organization(s) does this outlet receive support, regulation or supervisory visits?  ***Do not read list and circle all that apply.*** |  |
| 1. MOH/CMPE | A |
| 1. Food and Drug Department | B |
| 1. Provincial Health Office | C |
| 1. District Health Office | D |
| 1. Health Poverty Action (HPA) | E |
| Other, ***specify*** [___________________________________________________] | X |
| M8. How often does this outlet receive a support, regulation or supervisor visit?  1 = One or more times per month  2 = One time per 3 months  3 = One time per 6 months  4 = One time per year  5 = Other, **specify** [__________________________________________________] | [___] |
| M9. Does this outlet record information about the number of patients that have received a malaria test or malaria treatment?  1 = Yes  0 = No ***Go to P10***  8 = Don’t know ***Go to P10*** | [___] |
| M10. May I see the record-keeping system?  ***Record 1 if the record keeping was observed and 0 if the record keeping system was not observed.***  1 = Record keeping system observed  0 = Record keeping system not observed | [___] |
| M11. Does the system record information about each individual patient (1 line in the register = 1 patient)?  1 = Yes  0 = No  8 = Don’t know | [___] |
| M12. Does the system keep a tally or count of the number of patients that have received a malaria test or malaria treatment for a certain period such as per day, per week or per month?  1 = Yes  0 = No  8 = Don’t know | [___] |
| M13. Are the numbers of patients that receive malaria testing or treatment reported to any other government or non-governmental organizations?  1 = Yes  0 = No ***Go to P10***  8 = Don’t know ***Go to P10*** | [___] |
| M14. To which organizations (authorities) are the numbers of patients tested or treated for malaria reported?  ***Do not read list and circle all that apply.*** |  |
| 1. CMPE | A |
| 1. Provincial authorities (including provincial hospital) | B |
| 1. District authorities (including district hospital) | C |
| 1. Other, specify [___________________________________________________] | X |

| M15. How often are the numbers of patients tested or treated for malaria reported?  1 = One time per month  2 = One time per quarter  3 = Other, **specify** [__________________________________________________] | [___] |
| --- | --- |
| M16. How do you share the patient data with these organizations? Read list and record 1 for yes, 0 for no. ***Read list. Record 1 for yes, 0 for no, 8 for don’t know*** |  |
| 1. By paper form/record | [___] |
| 1. By phone - SMS (text) message | [___] |
| 1. By phone - electronic form | [___] |
| 1. By fax | [___] |
| 1. By phone - verbal report | [___] |
| 1. In person - verbal report | [___] |
| 1. By computer - electronic form | [___] |

| ***Interviewer:*** For the following questions, record the antimalarial brand name or generic name, and dosage form, in the spaces provided. Ask the provider to show you the medicine if it is in stock to verify the name and dosage form. |
| --- |

| P10. In your opinion, for treating uncomplicated malaria in adults, what is the most effective treatment?  ***Ask the provider to show you the medicine(s) if it is in stock.***   \| **Generic or brand name** \| **Dosage form/formulation** \| \| \| \| --- \| --- \| --- \| --- \| \| 01 = Tablet  02 = Suppository  03 = Granule \| 04 = Syrup  05 = Suspension  06 = IM/IV Injection (liquid or powder) \| 07 = Drops  95 = None specified  98 = Don’t know \| \| [______________________________________]  ***Don’t know = 98*** \| [___\|___] \| \| \|  \| **Generic or brand name** \| **Dosage form/formulation** \| \| \| \| --- \| --- \| --- \| --- \| \| 01 = Tablet  02 = Suppository  03 = Granule \| 04 = Syrup  05 = Suspension  06 = IM/IV Injection (liquid or powder) \| 07 = Drops  95 = None specified  98 = Don’t know \| \| [______________________________________]  ***Don’t know = 98*** \| [___\|___] \| \| \|  \| **Generic or brand name** \| **Dosage form/formulation** \| \| \| \| --- \| --- \| --- \| --- \| \| 01 = Tablet  02 = Suppository  03 = Granule \| 04 = Syrup  05 = Suspension  06 = IM/IV Injection (liquid or powder) \| 07 = Drops  95 = None specified  98 = Don’t know \| \| [______________________________________]  ***Don’t know = 98*** \| [___\|___] \| \| \| |
| --- | --- | --- | --- | --- | --- | --- | --- | --- | --- | --- | --- | --- | --- | --- | --- | --- | --- | --- | --- | --- | --- | --- | --- | --- | --- | --- | --- | --- | --- | --- | --- | --- | --- |
|  |

| P13. What treatment for uncomplicated malaria in adults do you most often recommend to customers?  ***Ask the provider to show you the medicine if it is in stock.***   \| **Generic or brand name** \| **Dosage form/formulation** \| \| \| \| --- \| --- \| --- \| --- \| \| 01 = Tablet  02 = Suppository  03 = Granule \| 04 = Syrup  05 = Suspension  06 = IM/IV Injection (liquid or powder) \| 07 = Drops  95 = None specified  98 = Don’t know \| \| [______________________________________]  ***Don’t know = 98*** \| [___\|___] \| \| \| |
| --- | --- | --- | --- | --- | --- | --- | --- | --- | --- | --- | --- |
| \| **Generic or brand name** \| **Dosage form/formulation** \| \| \| \| --- \| --- \| --- \| --- \| \| 01 = Tablet  02 = Suppository  03 = Granule \| 04 = Syrup  05 = Suspension  06 = IM/IV Injection (liquid or powder) \| 07 = Drops  95 = None specified  98 = Don’t know \| \| [______________________________________]  ***Don’t know = 98*** \| [___\|___] \| \| \|  \| **Generic or brand name** \| **Dosage form/formulation** \| \| \| \| --- \| --- \| --- \| --- \| \| 01 = Tablet  02 = Suppository  03 = Granule \| 04 = Syrup  05 = Suspension  06 = IM/IV Injection (liquid or powder) \| 07 = Drops  95 = None specified  98 = Don’t know \| \| [______________________________________]  ***Don’t know = 98*** \| [___\|___] \| \| \| |

| P16. Please name the first line treatment recommended by the government to treat uncomplicated *Plasmodium falciparum* malaria for an adult (60kg).  ***Do not read list. Circle ALL responses given.*** |  |
| --- | --- |
| Artemether Lumefantrine (Artefan, Coartem, Combiart) | A |
| Primaquine | B |
| Other ***specify***: [_____________________________________________________] | X |
| Don’t know | Z |
| ***SKIP INSTRUCTIONS***  ***If Artemether Lumefantrine was circled >>> continue to P17a***  ***If Artemether Lumefantrine was not circled >>> continue to P20a*** | |

| P17a. Please explain the government-recommended treatment regimen for **Artemether Lumefantrine** to treat **uncomplicated *Plasmodium falciparum* malaria** for an **adult (60kg)**.  ***If the provider has the medicine(s) available, use the package to complete the medicine(s) details.***  ***If the medicine(s) is not available, ask the provider to identify the medicine(s) from the prompt card.***  ***If identification of the medicine(s) is not possible, ask the provider to recall the medicine(s) details.***  *i. What is the strength of Artemether?*  *ii. What is the strength of Lumefantrine?*  ***Read the following 3 questions to the provider (do not record this information from the package):***  iv. How many tablets of Artemether Lumefantrine should they take at a time?  v. How many times per day should Artemether Lumefantrine be taken?  vi. Over how many days should Artemether Lumefantrine be taken? | [__\|__\|__].[__]mg  [__\|__\|__].[__]mg  ***Don’t know = 999.8***  [___\|___]**.**[___\|___]  [___\|___]  [___\|___]  ***Don’t know = 98*** |
| --- | --- |

| P18. Please name the first-line treatment recommended by the government to treat uncomplicated *Plasmodium vivax* malaria for an adult (60kg).  ***Do not read list. Circle ALL responses given.*** |  |
| --- | --- |
| Artemether Lumefantrine (Artefan, Coartem, Combiart) | A |
| Primaquine | B |
| Other ***specify***: [_____________________________________________________] | X |
| Don’t know | Z |
| ***SKIP INSTRUCTIONS***  ***If Artemether Lumefantrine was circled >>> continue to P19a***  ***If Artemether Lumefantrine and Primaquine were both not circled >>> continue to P20a***  ***If Primaquine only was circled >>> continue to P20b*** | |

| P19a. Please explain the government-recommended treatment regimen for **Artemether Lumefantrine** to treat **uncomplicated *Plasmodium vivax* malaria** for an **adult (60kg)**.  ***If the provider has the medicine(s) available, use the package to complete the medicine(s) details.***  ***If the medicine(s) is not available, ask the provider to identify the medicine(s) from the prompt card.***  ***If identification of the medicine(s) is not possible, ask the provider to recall the medicine(s) details.***   1. *What is the strength of Artemether?*   *ii. What is the strength of Lumefantrine?*  ***Read the following 3 questions to the provider (do not record this information from the package).***  iv. How many tablets of Artemether Lumefantrine should they take at a time?  v. How many times per day should Artemether Lumefantrine be taken?  vi. Over how many days should Artemether Lumefantrine be taken? | [__\|__\|__].[__]mg  [__\|__\|__].[__]mg  ***Don’t know = 999.8***  [___\|___]**.**[___\|___]  [___\|___]  [___\|___]  ***Don’t know = 98*** |
| --- | --- |
| ***SKIP INSTRUCTIONS***  ***If Primaquine was circled in P18 >>> continue to P19b***  ***If Primaquine was not circled in P18 >>> continue to P20a*** | |

| P19b. Please explain the government-recommended treatment regimen for **Primaquine** to treat **uncomplicated *Plasmodium vivax* malaria** for an **adult (60kg)**.  ***If the provider has the medicine(s) available, use the package to complete the medicine(s) details.***  ***If the medicine(s) is not available, ask the provider to identify the medicine(s) from the prompt card.***  ***If identification of the medicine(s) is not possible, ask the provider to recall the medicine(s) details.***  *i. What is the strength of Primaquine?*  ***Read the following 3 questions to the provider (do not record this information from the package).***  iii. How many tablets of Primaquine should they take at a time?  iv. How many times per day should Primaquine be taken?  v. Over how many days should Primaquine be taken? | [__\|__\|__].[__]mg  ***Don’t know = 999.8***  [___\|___]**.**[___\|___]  [___\|___]  [___\|___]  ***Don’t know = 98*** |
| --- | --- |
| ***SKIP INSTRUCTIONS***  ***Continue to P20b*** | |

| P20a. Have you seen or heard of a medicine called primaquine before?  1 = Yes ***go to P20b***  0 = No  ***go to P21***  8 = Don’t know ***go to P21*** | [___] |
| --- | --- |
| P20b. What is primaquine used for?  ***Do not read list. Circle ALL responses given.*** |  |
| As an **antimalarial medicine** / to **treat malaria** | A |
| As a **gametocyte** / to **prevent transmission** of malaria | B |
| As a **radical cure**/treatment for ***P. vivax*** malaria / to **prevent relapse** of ***P. vivax*** malaria | C |
| Don't know | Z |
| P20c. Have you ever prescribed primaquine for a patient/customer?  1 = Yes  0 = No | [___] |
| P20d. Have you ever provided or sold primaquine to a patient/customer?  1 = Yes  0 = No | [___] |

| P21. Malaria rapid diagnostic tests, also called RDTs, are small, individually wrapped blood tests that are able to quickly diagnose whether a person has malaria. ***Show RDT images in prompt card.***  Have you ever seen or heard of malaria RDTs?  1 = Yes ***go to P22***  0 = No  ***go to Section 6***  8 = Don’t know ***go to Section 6*** | [___] |
| --- | --- |
| P22. Have you ever tested a client for malaria using an RDT?  1 = Yes  0 = No  8 = Don’t know | [___] |
| P23. Would you ever recommend a patient/customer take an antimalarial if a blood test using a rapid diagnostic test produced a negative test result for malaria? **Read list. Record only one response.**  1 = Yes, Sometimes  2 = Yes, Always  3 = No, Never ***go to Section 6***  8 = Don’t know ***go to Section 6*** | [___] |
| P24. Under what circumstances would you recommend a patient/customer take an antimalarial following a negative RDT test for malaria? ***Do not read list****.* ***Prompt “anything else” until the respondent is finished.***  ***Circle ALL responses given*** |  |
| When they have signs/symptoms of malaria | A |
| When they ask for antimalarial treatment | B |
| When they are a child | C |
| When they are an adult | D |
| When they are a pregnant woman | E |
| When I do not trust/believe the test | F |
| When I know the patient/customer | G |
| Other (specify) [_______________________________________________________] | X |

***Complete the audit sheet tracker on the next page then follow the instructions for ending the interview.***

| **Section 6: Audit Tracking Sheet** |
| --- |

| T1. Were there any antimalarial TABLETS/SUPPOSITORIES/GRANULES in stock at this outlet?  1 = Yes  0 = No ***go to T4***  8 = Don’t know ***go to T4*** | [___] |
| --- | --- |
| T2. Total number of TABLET/SUPPOSITORY/GRANULE audit sheets completed | [___\|___] |
| T3. Did you complete audit sheet information for all available TABLETS/SUPPOSITORIES/GRANULES*?*  1 = Yes, audit complete  0 = No, audit not complete | [___] |

| T4. Were there any antimalarial NON TABLETS (Syrups, suspensions, Injectables) in stock at this outlet?  1 = Yes  0 = No ***go to T7***  8 = Don’t know ***go to T7*** | [___] |
| --- | --- |
| T5. Total number of NON-TABLET audit sheets completed | [___\|___] |
| T6. Did you complete audit sheet information for all available NON-TABLETS*?*  1 = Yes, audit complete  0 = No, audit not complete | [___] |

| T7. Were there any RDTs in stock at this outlet?  1 = Yes  0 = No ***go to T 10***  8 = Don’t know ***go to T10*** | [___] |
| --- | --- |
| T8. Total number of RDT audit sheets completed | [___\|___] |
| T9. Did you complete audit sheet information for all available RDT*?*  1 = Yes, audit complete  0 = No, audit not complete | [___] |
| T10. Were there any products currently out of stock but stocked within the past 3 months at this outlet?  1 = Yes  0 = No ***go to T12***  8 = Don't know ***go to T12*** | [___] |
| T11.Total number of stock-out products recorded in question A17a | [___\|___] |

| T12. COMMENTS: Reason for incomplete audit sheets (if response is no to T3, T6, or T9): |
| --- |

***Proceed to C9 and record the final status of the interview and time completed, then complete Section X: Ending the Interview***.
