# Supplementary material for: The malaria testing and treatment landscape in the southern Lao People’s Democratic Republic (PDR)
Source: Malar J. 2017 Apr 25;16:169. doi: 10.1186/s12936-017-1769-0 (PMC5404290; doi:10.1186/s12936-017-1769-0)
Supplement: Supplementary file 2 — Additional file 2. The Lao PDR ACTwatch Survey Questionnaire (Lao). [file 12936_2017_1769_MOESM2_ESM.pdf]

ລະຫັດຮ້ານຈຳໜ່າຍ: [___|___]-[___|___]-[___|___|___]-[___|___|___]

# ແບບຊຸດສອບຖາມຮ້ານຈຳໜ່າຍ (OS)

**ການສຳຫຼວດຮ້ານຈຳໜ່າຍ ACTwatch** **ສປປລາວ 2015**

| **ພາກທີ 1: ການສຳຫຼວດຂໍ້ມູນທັງໝົດ** |
|---|

***ໃຫ້ສຳພາດທຸກໆຮ້ານ ສຳລັບພາກນີ້***

| | |
|---|---|
| **ລະຫັດຮ້ານຈຳໜ່າຍ:** ນັກສຳພາດ-ເມືອງ-ກຸ່ມບ້ານ-ລະຫັດຮ້ານຈຳໜ່າຍ | [___|___]-[___|___]-[___|___|___]--[___|___|___] |
| C1. ວັນທີຂອງມື້ນີ້ (ວວ/ດດ/ປປປປ) | [___|___]-[___|___]-[_2_|_0_|_1_|_5_] |
| C2. ຊື່ນັກສຳພາດ [________________________________] | C2a. ລະຫັດຂອງນັກສຳພາດ [___|___] |
| C3. ຊື່ເມືອງ [________________________________] | C3a. ລະຫັດເມືອງ [___|___] |
| C4. ຊື່ກຸ່ມບ້ານ [________________________________] | C4a. ລະຫັດກຸ່ມບ້ານ [___|___|___] |
| C5. ຊື່ບ້ານ [________________________________] | |
| C6. ຊື່ຮ້ານຄ້າ, ***ຖ້າບໍ່ມີຊື່, ໃຫ້ບັນທຶກ "ບໍ່ມີຊື່ " ຫຼື ໃຫ້ໃສ່ຊື່ "ເຈົ້າຂອງຮ້ານ"*** [________________________________] | C6a. ລະຫັດຮ້ານຈຳໜ່າຍ [___|___|___] |

| C7. ປະເພດຮ້ານຈຳໜ່າຍ | | | |
|---|---|---|---|
| 01 ໂຮງໝໍແຂວງ | 08 ໂຮງໝໍເອກະຊົນ | 14 ຮ້ານຂາຍຢາທີ່ໄດ້ບໍ່ຂຶ້ນທະບຽນ | |
| 02 ໂຮງໝໍເມືອງ | 09 ຫ້ອງກວດພະຍາດເອກະຊົນ (Clinic) | 15 ຮ້ານນ້ອຍຂາຍເຄື່ອງຍ່ອຍໃນໝູ່ບ້ານ /ຮ້ານຂາຍເຄື່ອງຍ່ອຍ | |
| 03 ສຸກສາລາ | 10 ຮ້ານຂາຍຢາທີ່ໃຫ້ບໍລິການການກວດພະຍາດນຳ | 16 ຄົນຂາຍເຄື່ອງເຄື່ອນທີ່ | |
| 04 ອາສາສະໝັກແພດຂັ້ນບ້ານ (ທີ່ໄດ້ຮັບຝຶກອົບຮົມຈາກສູນໄຂ້ຍຸງ) | 11 ຮ້ານຂາຍຢາ ຂັ້ນ 1 | 17 ຫ້ອງວິເຄາະເອກະຊົນ | [___|___] |
| 05 ໂຮງໝໍທະຫານ/ຕຳຫລວດ | 12 ຮ້ານຂາຍຢາ ຂັ້ນ 2 | 18 ຫ້ອງວິເຄາະຂອງອົງການທີ່ບໍ່ຂຶ້ນກັບລັດຖະບານ (NGO)/ໂຄງການໄດ້ຮັບທຶນຈາກສາສະໜາ | |
| 07 ຄີຣນິກຂອງອົງການທີ່ບໍ່ຂຶ້ນກັບລັດຖະບານ (NGO)/ ຄຣີນິກທີ່ໄດ້ຮັບທຶນຈາກສາສະໜາ | 13 ຮ້ານຂາຍຢາ ຂັ້ນ 3 | 19 ອາສາສະໝັກແພດບ້ານ (ທີ່ໄດ້ຮັບຝຶກອົບຮົມຈາກອົງການທີ່ບໍ່ຂຶ້ນກັບລັດຖະບານ) | |
| | | 96 ອື່ນໆ ***(ລະບຸ)*** [________________] | |

| | |
|---|---|
| C8. ກຸ່ມບ້ານນີ້ແມ່ນສ່ວນໜຶ່ງຂອງກຸ່ມຕົວຢ່າງຂອງ ບູດສະເຕີ້ ບໍ່? 1 = ແມ່ນ 0 = ບໍ່ແມ່ນ | [___] |

ລະຫັດຮ້ານຈຳໜ່າຍ: [___|___]-[___|___]-[___|___|___]-[___|___|___]

ສະບາຍດີ, ຂ້າພະເຈົ້າຊື່ __________, ຂ້າພະເຈົ້າເຮັດວຽກໃນນາມຂອງ ອົງການສາກົນ ບໍລິການປະຊາຊົນ (ພີເອສໄອ). ພວກເຮົາກຳລັງດຳເນີນການສຶກສາກ່ຽວກັບການມີຢາປິ່ນປົວໄຂ້ມາເລເລຍ ແລະ ການໃຫ້ບໍລິການການກວດເພື່ອບົ່ງມະຕິພະຍາດໄຂ້ມາເລເລຍ. ຜົນໄດ້ຮັບຈາກການສຶກສາຄັ້ງນີ້ຈະຖືກນຳໃຊ້ເຂົ້າໃນການປັບປຸງ ການມີຢາປິ່ນປົວໄຂ້ມາເລເລຍ ທີ່ເໝາະສົມຢູ່ປະເທດລາວ. ຂ້າພະເຈົ້າຂໍຖາມຄຳຖາມ ທ່ານເພື່ອເບິ່ງວ່າທ່ານຈະເປັນສ່ວນໜຶ່ງ ໃນການສຳຫຼວດຄັ້ງນີ້ບໍ່.

**ພາກທີ 2: ການຄັດເລືອກ & ເງື່ອນໄຂທີ່ເໝາະສົມ**

| | |
|---|---|
| S1. ມື້ນີ້, ທ່ານມີຢາຊະນິດໃດໜຶ່ງບໍ່?<br>1 = ມີ *ໄປຂໍ້ S3*<br>0 = ບໍ່ມີ | [___] |
| S2.ມື້ນີ້, ມີຢາຊະນິດໃດໜຶ່ງບໍ່ທີ່ບໍ່ມີໃນຮ້ານ, ແຕ່ວ່າເຄີຍມີໃນຮ້ານ **<u>ໃນໄລຍະ 3 ເດືອນຜ່ານມາ</u>?**<br>1 = ມີ *ໄປຂໍ້ S4*<br>0 = ບໍ່ມີ *ໄປຂໍ້ S5*<br>8 = ບໍ່ຮູ້ *ໄປຂໍ້ S5* | [___] |
| S3. ມື້ນີ້, ທ່ານມີຢາປິ່ນປົວໄຂ້ມາເລເລຍບໍ່?<br>1 = ມີ *ເອົາເຈ້ຍຂໍ້ມູນຂອງການສຶກສາໃຫ້ ແລະ ຖາມຄວາມຍິນຍອມໃນການເຂົ້າຮ່ວມ. ບັນທຶກເວລາເລີ່ມ ໃນ C9. ໄປຂໍ້ທີ່ ພາກທີ 3: ການກວດສອບຢາປົວໄຂ້ມາເລເລຍ .*<br>0 = ບໍ່ມີ **ກວດກາຄວາມຖືກຕ້ອງດ້ວຍບັດສະແດງ.** *ໄປຂໍ້ S4* | [___] |
| S4. ມື້ນີ້, ມີຢາປິ່ນປົວໄຂ້ມາເລເລຍທີ່ບໍ່ມີໃນຮ້ານບໍ່, ແຕ່ວ່າເຄີຍມີໃນຮ້ານ **<u>ໃນໄລຍະ 3 ເດືອນຜ່ານມາ</u>?**<br>1 = ມີ *ເອົາເຈ້ຍຂໍ້ມູນຂອງການສຶກສາໃຫ້ ແລະ ຖາມຄວາມຍິນຍອມໃນການເຂົ້າຮ່ວມ. ບັນທຶກເວລາເລີ່ມ ໃນ C9. ໄປຂໍ້ທີ່ A17.*<br>0 = ບໍ່ມີ **ກວດກາຄວາມຖືກຕ້ອງດ້ວຍບັດສະແດງ.** *ໄປຂໍ້ S5*<br>8 = *ບໍ່ຮູ້* **ກວດກາຄວາມຖືກຕ້ອງດ້ວຍບັດສະແດງ.** *ໄປຂໍ້ S5* | [___] |
| S5. ມື້ນີ້, ທ່ານໄດ້ໃຫ້ບໍລິການບົ່ງມະຕິ ຫຼື ໄດ້ຂາຍອຸປະກອນໃນການກວດເພື່ອບົ່ງມະຕິບໍ່?<br>1 = ມີ **ໄປຂໍ້** *S6*<br>0 = ບໍ່ມີ *ກວດກາຄວາມຖືກຕ້ອງດ້ວຍບັດສະແດງ. ບັນທຶກລາຍລະອຽດໃນ C9 ແລ້ວຕື່ມຂໍ້ມູນໃສ່ ພາກ X: ການຈົບການສຳພາດ* | [___] |
| S6. ການບໍລິການ ຫຼື ອຸປະກອນກວດເຫຼົ່ານີ້ແມ່ນໃຊ້ສຳລັບກວດຫາກຳລະນີສົງໃສເປັນໄຂ້ມາເລເລຍ ບໍ່?<br>1 = ມີ *ເອົາເຈ້ຍຂໍ້ມູນຂອງການສຶກສາໃຫ້ ແລະ ຖາມຄວາມຍິນຍອມໃນການເຂົ້າຮ່ວມ. ບັນທຶກເວລາເລີ່ມ ໃນ C9. ໄປພາກທີ* 4: *ກວດສອບການບົ່ງມະຕິ*<br>0 = ບໍ່ມີ *ກວດກາຄວາມຖືກຕ້ອງດ້ວຍບັດສະແດງ. ບັນທຶກລາຍລະອຽດໃນ C9 ແລ້ວຕື່ມຂໍ້ມູນໃສ່ ພາກ X: ການຈົບການສຳພາດ* | [___] |

*ກ່ອນການດຳເນີນການສຳພາດ ແບບຄົບຖ້ວນໃຫ້ໝັ້ນໃຈວ່າ ທ່ານ<u>ໄດ້ໃຫ້ເຈ້ຍຂໍ້ມູນຂອງການສຶກສານີ້ແກ່ຜູ້ຖືກສຳພາດ, ໄດ້ອະທິບາຍການສຶກສາ</u> ແລະ <u>ໄດ້ຂໍຄວາມຍິນຍອມໃນການເຂົ້າຮ່ວມແລ້ວ</u>*

ລະຫັດຮ້ານຈຳໜ່າຍ: [___|___]-[___|___]-[___|___|___]-[___|___|___]

C9. ຜົນຂອງການຢ້ຽມຢາມ

| ວັນທີ<br>(ວວ/ດດ/ປປ) | ການຢ້ຽມຢາມຄັ້ງທີ 1 | ການຢ້ຽມຢາມຄັ້ງທີ 2 | ການຢ້ຽມຢາມຄັ້ງທີ 3 |
|---|---|---|---|
| | [___\|___]-[___\|___]-[_1_\|_5_] | [___\|___]-[___\|___]-[_1_\|_5_] | [___\|___]-[___\|___]-[_1_\|_5_] |
| ເວລາເລີ່ມ<br>(ບັນທຶກເປັນ 24 ຊົ່ວໂມງ) | [___\|___]:[___\|___] | [___\|___]:[___\|___] | [___\|___]:[___\|___] |
| ເວລາທີ່ສຳເລັດ<br>(ບັນທຶກເປັນ 24 ຊົ່ວໂມງ) | [___\|___]:[___\|___] | [___\|___]:[___\|___] | [___\|___]:[___\|___] |
| ຜົນທີ່ໄດ້ | [___\|___] | [___\|___] | [___\|___] |

01 = ຮ້ານຈຳໜ່າຍທີ່ເໝາະສົມ ແລະ ໄດ້ສຳເລັດການສຳຫຼວດ *ໄປຂໍ້ E1*
02 = ຮ້ານຈຳໜ່າຍທີ່ບໍ່ເໝາະສົມ: ບໍ່ເໝາະສົມກັບເງື່ອນໄຂໃດໜຶ່ງໃນພາກຄັດເລືອກ *ໄປຂໍ້ E1*
03 = ໄດ້ຢຸດການສຳພາດເພາະຖືກລົບກວນ/ຂັດຈັງຫວະ *ໄປຂໍ້ C11*
04 = ຜູ້ຖືກສຳພາດບໍ່ຢູ່/ບໍ່ສະດວກໃນເວລານີ້ *ໄປຂໍ້ C11*
05 = ຮ້ານຈຳໜ່າຍບໍ່ເປີດໃນເວລານັ້ນ *ໄປຂໍ້ C11*
06 = ຮ້ານຈຳໜ່າຍປິດຖາວອນ *ໄປຂໍ້ E1*
96 = ອື່ນໆ **(ລະບຸ)**[________________________________]
97 = ປະຕິເສດການເຂົ້າຮ່ວມ *ໄປຂໍ້ C10*

C10. ຖ້າຜູ້ໃຫ້ບໍລິການປະຕິເສດການສຳພາດ, ຍ້ອນຫຍັງ? [___]

1 = ລູກຄ້າຫຼາຍ *ຖາມຜູ້ຖືກສຳພາດສຳລັບເວລາທີ່ເຂົາເຈົ້າສະດວກ ແລະ ບັນທຶກໄວ້ໃນ C11*
2 = ຄິດວ່າມັນແມ່ນການກວດສອບ / ກັງວົນກ່ຽວກັບໃບອະນຸຍາດ *ໄປຂໍ້ E1*
3 = ບໍ່ມີຄວາມສົນໃຈ *ໄປຂໍ້ E1*
6 = ອື່ນໆ **(ລະບຸ)**:[________________________________]
7 = ປະຕິເສດທີ່ຈະໃຫ້ເຫດຜົນ *ໄປຂໍ້ E1*

C11. *ນຳໃຊ້ບ່ອນວ່າງນີ້ໃນການບັນທຶກລາຍລະອຽດເວລາກັບໄປສຳພາດຄືນ. ຖ້າວ່າບໍ່ສາມາດສຳເລັດການສຳພາດໄດ້ໃນເວລາອື່ນ, ໄປຂໍ້ E1*

## ພາກ X: ການຈົບການສຳພາດ

E1. ຊື່ຜູ້ຖືກສຳພາດ:

[________________________________]

5 = *ບໍ່ມີຂໍ້ມູນ, ບໍ່ມີຜູ້ໃຫ້ສຳພາດ*; 7 = *ປະຕິເສດ*

E2. ທີ່ຢູ່ ຫຼື ຂໍ້ມູນທີ່ຕັ້ງຂອງຮ້ານຈຳໜ່າຍ (ບໍ່ແມ່ນຕູ້ ປນ)
(ໃຫ້ການອະທິບາຍທີ່ລະອຽດ ທີ່ຈະຊ່ວຍໃຫ້ທີວໜ້າທີມ ໃນການຊອກຫາຮ້ານຈຳໜ່າຍ)

E3. ເບີໂທລະສັບ (ເບີໂທຫລັກ)

________________________________

*99999999995* = ບໍ່ມີຜູ້ໃຫ້ສຳພາດ/ ບໍ່ມີເບີໂທລະສັບ
*99999999997* = ປະຕິເສດ

E4. *ເສັ້ນຂະໜານ*:
[_N_] - [___|___] . [___|___|___|___|___|___]

E5. *ເສັ້ນແວງ*:
[_E_] - [___|___|___] . [___|___|___|___|___|___]

E7. **ການສັງເກດເພີ່ມເຕີມໂດຍນັກສຳພາດ (ຖ້າມີ)**

***ຂອບໃຈຜູ້ໃຫ້ບໍລິການ ແລະ ຈົບການສຳພາດ***

(Blank page)

| ພາກທີ 3: ກວດສອບຢາປິ່ນປົວພະຍາດໄຂ້ມາເລເລຍ |
|---|

| |
|---|
| *A0. ອ່ານໃຫ້ຜູ້ໃຫ້ບໍລິການຟັງ:*<br>ພວກເຮົາຂໍເບິ່ງລາຍການ ທີ່ທ່ານມີຢູ່ໃນຮ້ານໃນປະຈຸບັນໄດ້ບໍ່? ທ່ານມີຢາຕາມລາຍຊື່ຢາດັ່ງລຸ່ມນີ້ບໍ່?<br>***ສະແດງລາຍການທັງໝົດໃຊ້ບັດສະແດງລາຍການຢາປິ່ນປົວໄຂ້ມາເລເລຍ ; ບໍ່ຕ້ອງບັນທຶກຄໍາຕອບ***<br>1. Artemether– Lumefantrim (*Coartem, Combiart*)<br>2. Chloroquine Phosphate (*Cloroquin Phosphat, Malacin, Maraquine, Nitaquin*)<br>3. Cholorquine (*Chloroquine, Nivaquine*)<br>4. Artesunate (*Artesunate, Artesun, Artesunat*)<br><br>***ຖ້າວ່າຮ້ານຈໍາໜ່າຍບໍ່ມີຢາປິ່ນປົວພະຍາດໄຂ້ຍູງຢູ່ໃນຮ້ານໃຫ້ ກວດເບິ່ງພາກຄັດເລືອກອີກເທື່ອໜຶ່ງແລ້ວໄປສູ່ຄໍາຖາມຂໍ້*** **A17.** |

- *ດໍາເນີນການກວດສອບຢາປິ່ນປົວພະຍາດໄຂ້ມາເລເລຍ. ແບບຟອມໃນການກວດສອບຢາປິ່ນປົວພະຍາດໄຂ້ມາເລເລຍ ຕ່າງໆຈະຖືກນໍາໃຊ້ໃນການບັນທຶກຂໍ້ມູນຢາປິ່ນປົວພະຍາດໄຂ້ມາເລເລຍ ອີງຕາມຮູບແບບຂອງຢາ .*
- *ແບບຟອມໃນການປິ່ນປົວພະຍາດໄຂ້ມາເລເລຍ ແບບຟອມກວດສອບຢາປິ່ນປົວໄຂ້ມາເລເລຍ ຊຸດອື່ນຈະຖືກນໍາໃຊ້ເພື່ອມາບັນທຶກຂໍ້ມູນມາປິ່ນປົວໄຂ້ມາເລເລຍ ໂດຍອີງຕາມຮູບແບບຂອງຢາ: (ຮູບແບບຢາເມັດ ແລະ ຮູບແແບບບໍ່ແມ່ນຢາເມັດ)*
- *ແຍກຢາປິ່ນປົວພະຍາດໄຂ້ມາເລເລຍ ເປັນສອງຈໍາພວກ:*
  - *ຈໍາພວກທໍາອິດແມ່ນປະກອບມີຢາປິ່ນປົວພະຍາດໄຂ້ມາເລເລຍ ທັງໝົດທີ່ຢູ່ໃນຮູບແບບເມັດ, ຢາຍັດ, ຫຼື ຢາຝຸ່ນເປັນຊອງ. ນໍາໃຊ້ແບບຟອມສໍາລັບກວດສອບຢາເມັດ, ຢາຍັດ ແລະ ຢາເປັນຝຸ່ນຊອງໃນການບັນທຶກຢາເຫຼົ່ານີ້.*
  - *ຈໍາພວກທີສອງແມ່ນປະກອບມີຢາປິ່ນປົວພະຍາດໄຂ້ມາເລເລຍ ທັງໝົດທີ່ຢູ່ໃນຮູບແບບອື່ນໆທີ່ບໍ່ແມ່ນຮູບແບບເມັດ, ຢາຍັດ, ຫຼື ແບບເປັນຝຸ່ນຊອງ .. ນໍາໃຊ້ແບບຟອມກວດສອບຢາທີ່ບໍ່ແມ່ນຢາເມັດໃນການບັນທຶກຢາເຫຼົ່ານີ້.*
- *ຖ້າວ່າຫາກຕ້ອງໃຊ້ເຈ້ຍ ຫຼື ແບບຟອມກວດສອບເພີ້ມຕື່ມອີກ, ໃຫ້ເອົາເຈ້ຍ ຫຼື ແບບຟອມກວດສອບເຫຼົ່ານັ້ນ ມາໜີບຕິດຂັດໃສ່ທາງດ້ານຫຼັງຂອງໂຕທີ່ຕໍ່.*
- *ທຸກໆໜ້າເຈ້ຍຈະຕ້ອງລຽງເລກລໍາດັບ ກ່ອນທີ່ຈະຍ້າຍໄປຮ້ານໃໝ່.*
- *ໝາຍເລກ**<u>ຢາແຕ່ລະຕົວ</u>**ໂດຍການກໍານົດໝາຍເລກຜະລິດຕະພັນ (ເລີ້ມຈາກ 1 ສໍາລັບຢາທີ່ເປັນ ຢາເມັດ, ຢາຍັດ, ຢາຝຸ່ນເປັນຊອງ ແລະ ເລີ້ມຈາກ 1 ອີກສໍາລັບຢາທີ່ ບໍ່ແມ່ນເມັດ)*
- *ໝາຍເລກ**<u>ເຈ້ຍກວດສອບແຕ່ລະໃບ</u>**ທີ່ໄດ້ນໍາໃຊ້ລົງໃນບ່ອນວ່າງດ້ານລຸ່ມຂອງໜ້າເຈ້ຍ,*

## *ຄໍາອະທິບາຍເພີ່ມເຕີມສໍາລັບລະຫັດໜ່ວຍງານຍ່ອຍ*

***ໃນບັນດາຮ້ານຈໍາໜ່າຍທັງໝົດ, ໃຫ້ຕື່ມລະຫັດ <u>ໜ່ວຍງານຍ່ອຍໃສ່ (ພ້ອມທັງໝາຍເລກຜະລິດຕະພັນ) ສໍາລັບແຕ່ລະຢາທີ່ໄດ້ຖືກກວດສອບ. ລະຫັດເຫຼົ່ານີ້ແມ່ນມີລາຍການດັ່ງລຸ່ມນີ້</u>***

| ***ລະຫັດໜ່ວຍງານຍ່ອຍ*** | |
|---|---|
| X | ບັນດາຮ້ານຈໍາໜ່າຍຕ່າງໆທີ່ມີ ຈຸດຈ່າຍ/ແຈກຢາຍຢາ ສໍາລັບຢາປິ່ນປົວ/ການບົ່ງມະຕິ **ຈຸດດຽວເທົ່ານັ້ນ.** |
| A | ພະແນກກວດເຂດນອກ / ບ່ອນຈ່າຍຢາ/ ຮ້ານຂາຍຢາຫຼັກ ( (ທີ່ຄົນເຈັບທັງໝົດມາໃຊ້ບໍລິການ). |
| B | ພະແນກກວດເຂດນອກສໍາລັບຜູ້ໃຫຍ່/ບ່ອນຈ່າຍຢາສໍາລັບຜູ້ໃຫຍ່/ຄລີນິກຜູ້ໃຫຍ່ |
| C | ພະແນກກວດເຂດນອກສໍາລັບເດັກ/ບ່ອນຈ່າຍຢາສໍາລັບເດັກ/ຄລີນິກເດັກ |
| D | ຝາກທ້ອງ / ຄລີນິກສໍາລັບແມ່ / ສຸຂະພາບແມ່ ແລະ ເດັກ |
| E | ຄລີນິກບໍລິການປິ່ນປົວພະຍາດເອດສ໌ (ART) / ຄລີນິກ HIV / ໂລກເອດສ໌ AIDS |
| F | ພະແນກ ພະຍາດຕິດຕໍ່ |
| G | ພະແນກສຸກເສີນ |
| H | ພະແນກຈ່າຍຢາທີ່ເປັນຂອງເອກະຊົນທີ່ຕັ້ງຢູ່ໃນສະຖານທີ່ບໍລິການສາທາລະນະສຸກຂອງລັດ |
| I | ໜ່ວຍງານວິເຄາະ (***ສໍາລັບການກວດບົ່ງມະຕິແບບວ່ອງໄວ***) |
| Z | ອື່ນໆ ***(ລະບຸລະອຽດກ່ຽວກັບປະເພດ ໃນບ່ອນວ່າງສໍາລັບຫ້ອງຄໍາເຫັນຢູ່ໃນເຈ້ຍກວດສອບເຈ້ຍກວດສອບ ຢາເມັດ, ຢາຍັດ, ຢາຝຸ່ນເປັນຊອງ (TSG) ຂໍ້ 15 ຫຼື ເຈ້ຍກວດສອບຢາທີ່ບໍ່ແມ່ນຊະນິດເມັດ (NT) ຂໍ້ 15)*** |

(Black page)

ເຈ້ຍກວດສອບ ຢາເມັດ, ຢາຢັດ, ຢາຝຸ່ນເປັນຊອງ **(TSG):** ລະຫັດຮ້ານຍ່ອຍ: [___|___]-[___|___]-[___|___|___]-[___|___|___]

| **ລະຫັດໜ່ວຍງານຍ່ອຍ** | | 1. **ຊື່ຕົວຢາ** | 2. **ຄວາມແຮງ** | **2a.ນີ້ເປັນຄ່າຄວາມແຮງພື້ນຖານບໍ່?** | 3. **ຮູບແບບຢາ/ລັກສະນະຢາ** | 4. **ຊື່ການຄ້າ/ຊື່ຍີ່ຫໍ້**<br>*(ລວມທັງຂໍ້ມູນ ນ້ຳໜັກ ແລະ ອາຍຸ)* |
|---|---|---|---|---|---|---|
| [_____]<br><br>**ໝາຍເລກຜະລິດຕະພັນ**<br><br>[__\|__] | [__\|__]<br>[__\|__]<br>[__\|__]<br><br>[__\|__] | ____________<br>____________<br>____________ | [__\|__\|__].[__]mg<br>[__\|__\|__].[__]mg<br>[__\|__\|__].[__]mg | [__] 1 = ແມ່ນ<br>[__] 0 = ບໍ່ແມ່ນ<br>[__] 8 = ບໍ່ຮູ້<br>**ຖ້າບໍ່ແມ່ນ, ໃຫ້ລະບຸສານເຄມີປະສົມ**<br>[____________________] | 1 = ຢາເມັດ<br>2 = ຢາຢັດ<br>3 = ຢາຝຸ່ນເປັນຊອງ<br>[___] | |

| 5. **ຜູ້ຜະລິດ** | 6. **ປະເທດທີ່ຜະລິດ** | 7. **ຂະໜາດຂອງຫໍ່**<br>**(Package size)** | 8. **ເປັນຕົວຢາທີ່ມີສ່ວນປະສົມຫຼາຍຕົວຢາຢູ່ໃນອັນດຽວ (FDC)** | 9. **ຜະລິດຕະພັນມີເຄື່ອງໝາຍໃບໄມ້ສີຂຽວບໍ່?** | 10. **ຈຳນວນທີ່ໄດ້ຂາຍ/ແຈກຢາຍໃຫ້ກັບລູກຄ້າຍ່ອຍທັງໝົດໃນ 7 ມື້ທີ່ຜ່ານມາ** *(ກໍລະນີ ບັນທຶກ ຈຳນວນຫໍ່ / ກະປ໋ອງ ແມ່ນຕ້ອງບັນທຶກ ຕາມຫົວໜ່ວຍທີ່ໄດ້ລະບຸໄວ້ໃນຄຳຖາມ ຂໍ້ 7 ຫຼື ສາມາດບັນທຶກ ຈຳນວນທັງໝົດຂອງຢາເມັດ / ຢາຢັດ /* ຢາຝຸ່ນເປັນຊອງ*ທີ່ໄດ້ຂາຍ*) | 11. **ເຄີຍມີຢາບໍ່ມີໃນຮ້ານ ໃນຊ່ວງເວລາໃດໜຶ່ງໃນໄລຍະ3 ເດືອນຜ່ານມາບໍ່?** |
|---|---|---|---|---|---|---|
| | [__\|__\|__] | ມີຈຳນວນທັງໝົດ<br>[___\|___\|___\|___]<br>**ເມັດ/ຢາຢັດ/ຢາຝຸ່ນເປັນຊອງ** ຢູ່ໃນແຕ່ລະ:<br>1 = ຫໍ່/ແຜງ<br>2 = ໂຖ/ກະປ໋ອງ<br>[___] | 1 = ແມ່ນ<br>0 = ບໍ່ແມ່ນ<br>8 = ບໍ່ຮູ້<br>[___] | 1 = ມີ<br>0 = ບໍ່ມີ<br>8 = ບໍ່ຮູ້<br>[___] | ຮ້ານຈຳໜ່າຍແຫ່ງນີ້ໄດ້ຂາຍ [___\|___\|___] **ຫໍ່/ແຜງ ຫຼືໂຖ/ກະປ໋ອງ** ໃນ7ມື້ຜ່ານມາ<br><br>**ຫຼື**<br><br>ຮ້ານຈຳໜ່າຍແຫ່ງນີ້ໄດ້ຂາຍ [___\|___\|___] **ຢາເມັດ/ຢາຢັດ ຫຼື ຫໍ່ຢາຝຸ່ນເປັນຊອງ** ໃນ 7 ມື້ຜ່ານມາ<br><br>***ບໍ່ມີຂໍ້ມູນ = 995; ປະຕິເສດ = 997; ບໍ່ຮູ້ = 998*** | 1 = ແມ່ນ<br>0 = ບໍ່ແມ່ນ<br>8 = ບໍ່ຮູ້<br>[___] |

| 12. ລາຄາຂາຍຍ່ອຍ | 13. ລາຄາຊື້ຍົກ (ຄັ້ງລ້າສຸດ) | 14. ເປັນຫຍັງທ່ານຈຶ່ງມີຢານີ້ [ສະແດງຜະລິດຕະພັນ]? | 15. ຄຳເຫັນອື່ນໆ | 16a. ຢານີ້ແມ່ນຢາເມັດບໍ່? |
|---|---|---|---|---|
| ຈຳນວນ<br><br>[___\|___\|___]<br>**ຢາເມັດ/ຢາຢັດ ຫຼື ຢາຝຸ່ນເປັນຊອງ**<br><br>ລູກຄ້າແຕ່ລະຄົນຕ້ອງຈ່າຍ<br><br>[___\|___\|___\|___\|___\|___]<br>**ກີບ** | ຈຳນວນ<br><br>[___\|___\|___\|___]<br>**ຢາເມັດ/ຢາຢັດ ຫຼື ຢາຝຸ່ນເປັນຊອງ**<br><br>ແມ່ນຊື້ມາໃນລາຄາ<br><br>[___\|___\|___\|___\|___\|___\|___]<br>**ກີບ** | *ບໍ່ຕ້ອງອ່ານລາຍການ*<br>*ຂີດວົງອ້ອມເອົາທຸກຄຳຕອບທີ່ຜູ້ຕອບຕອບ*<br><br>ໄດ້ຮັບຢາລ້າ A<br>ມີກຳໄລ B<br>ຖືກແນະນຳໂດຍພາກລັດ C<br>ລາຄາຖືກ D<br>ຄວາມຕ້ອງການ ຫຼື ຄວາມນິຍົມຂອງລູກຄ້າ E<br>ຊື່ສຽງທາງບວກຂອງເຄື່ອງໝາຍສິນຄ້າ F<br>ໄດ້ຮັບການສັ່ງໂດຍທ່ານໝໍເລື້ອຍໆ G<br>ໄດ້ຮັບຜົນດີທີ່ສຸດສຳລັບການປິ່ນປົວໄຂ້ມາເລເລຍ H<br>ບໍ່ຮູ້ X<br>ອື່ນໆ Z<br>ອະທິບາຍລະອຽດ<br>[______________________________] | | 1 = ແມ່ນ<br>0 = ບໍ່ແມ່ນ [___]<br>*ຖ້າບໍ່ແມ່ນ, ໃຫ້ໄປຕໍ່ທີ່ແບບຟອມຕໍ່ໄປ.*<br><br>**16b. ຢາໂຕນີ້ມີສານອອກລິດພຽງຕົວດຽວບໍ່?**<br>1 = ແມ່ນ<br>0 = ບໍ່ແມ່ນ [___]<br>*ຖ້າບໍ່ແມ່ນ, ໃຫ້ໄປຕໍ່ທີ່ແບບຟອມຕໍ່ໄປ*<br><br>**16c. ສານອອກລິດດັ່ງກ່າວແມ່ນໜຶ່ງໃນສານດັ່ງຕໍ່ໄປນີ້ບໍ່?**<br>ອາເຕເມເທີ (ARTEMETHER)<br>ອາເຕຊູເນັ (ARTESUNATE)<br>ດີຮາຍໂດອາເຕມີຊີນິນ (DIHYDROARTEMISININ)<br><br>1 = ແມ່ນ<br>0 = ບໍ່ແມ່ນ [___]<br><br>*ຖ້າວ່າແມ່ນ, ໃຫ້ຕື່ມພາກ ຢາກິນປິ່ນປົວໄຂ້ມາເລເລຍຊະນິດດຽວອາເຕີມີຊີນິນ (Oral AMT) ຫຼັງຈາກທີ່ພາກ "ຢາປິ່ນປົວພະຍາດໄຂ້ມາເລເລຍ ທີ່ຂາດສາງ" ໄດ້ຖືກຕື່ມຄົບແລ້ວ* |
| *ບໍ່ເສຍຄ່າ = 000000*<br>*ປະຕິເສດ = 999997*<br>*ບໍ່ຮູ້ = 999998* | *ບໍ່ເສຍຄ່າ = 000000*<br>*ປະຕິເສດ = 999997*<br>*ບໍ່ຮູ້ = 999998* | | | |

เจ้ยกวดสอบยาที่บ่แม่นຊະນິດເມັດ **(NT):** ຢານ້ຳເຊື່ອມ, ຢານ້ຳແຂວນຕະກອນ, ຢາສັກ **&** ອື່ນໆ ລະຫັດຮ້ານຈຳໜ່າຍ: [___|___]-[___|___]-[___|___|___]-[___|___|___]

| **ລະຫັດໜ່ວຍງານຍ່ອຍ** | | **1. ຊື່ຕົວຢາ** | **2. ຄວາມແຮງ** | **2a.ນີ້ເປັນຄ່າຄວາມແຮງພື້ນຖານບໍ່?** | **3. ຮູບແບບຢາ/ປະເພດ** |
|---|---|---|---|---|---|
| [_____]<br><br>**ໝາຍເລກຜະລິດຕະພັນ**<br><br>[__\|__] | [__\|__]<br>[__\|__]<br>[__\|__]<br><br>[__\|__] | ____________<br>____________<br>____________ | [__\|__\|__\|__].[__] (mg) /[__\|__\|__] .[__] (mL)<br>[__\|__\|__\|__].[__] (mg) /[__\|__\|__] .[__] (mL)<br>[__\|__\|__\|__].[__] (mg) /[__\|__\|__] .[__] (mL)<br><br>**(ໝາຍເຫດ*:* ຖ້າແມ່ນຢາສັກທີ່ເປັນປະເພດຝຸ່ນ, ໃຫ້ບັນທຶກໃສ່ຫ້ອງ *mg* ແລະ ບໍ່ໃຫ້ຕື່ມໃສ່ຫ້ອງ *mL*.)** | [__]<br>[__]<br>[__]<br><br>1 = ແມ່ນ<br>0 = ບໍ່ແມ່ນ<br>8 = ບໍ່ຮູ້<br><br>**ຖ້າ ບໍ່ແມ່ນ ຫຼື ໝາຍ "0", ໃຫ້ລະບຸສານເຄມີປະສົມ**<br>[____________________] | 1 = ຢານ້ຳເຊື່ອມ<br>2 = ຢານ້ຳແຂວນຕະກອນ<br>3 = ຢາສັກທີ່ເປັນປະເພດນ້ຳ<br>4 = ຢາສັກທີ່ເປັນປະເພດຝຸ່ນ<br>5 = ຢາຢອດ<br>6 = ອື່ນໆ **(ອະທິບາຍລະອຽດ)**<br><br>[____________________]<br><br>[___] |

| **4.ຊື່ການຄ້າ/ຊື່ຍີ່ຫໍ້**<br>*(ລວມທັງຂໍ້ມູນນ້ຳໜັກ ແລະ ອາຍຸ)* | **5. ຜູ້ຜະລິດ** | **6. ປະເທດທີ່ຜະລິດ** | **7. ຂະໜາດບັນຈຸພັນ (Package size)** | **9. ຜະລິດຕະພັນມີເຄື່ອງໝາຍໃບໄມ້ສີຂຽວບໍ່?** | **10. ຈຳນວນທີ່ໄດ້ຂາຍ/ແຈກຢາຍໃຫ້ກັບລູກຄ້າບຸກຄົນໃນ 7 ມື້ທີ່ຜ່ານມາ** | **11.ເຄີຍມີຢາບໍ່ມີໃນຮ້ານໃນຊ່ວງເວລາໃດໜຶ່ງໃນໄລຍະ 3 ເດືອນຜ່ານມາບໍ່?** |
|---|---|---|---|---|---|---|
| | | [__\|__\|__] | ທັງໝົດມີ<br><br>[___\|___\|___\|___].[___] **mL**<br>**(ຫຼື mg ສຳລັບຢາສັກທີ່ເປັນປະເພດຝຸ່ນ)** ໃນແຕ່ລະ:<br><br>1 = ຂວດແກ້ວ<br>2 = ຫຼອດແກ້ວສຳລັບຢາສັກ<br><br>[___] | 1 = ແມ່ນ<br>0 = ບໍ່ແມ່ນ<br>8 = ບໍ່ຮູ້<br><br>[___] | ຮ້ານຈຳໜ່າຍນີ້ໄດ້ຂາຍ/ແຈກຢາຍ<br><br>[___\|___\|___\|___]<br>**ຂວດແກ້ວ ຫຼື ຫຼອດແກ້ວສຳລັບຢາສັກ** ໃນ 7 ມື້ທີ່ຜ່ານມາ<br><br>***ປະຕິເສດ = 9997***<br>***ບໍ່ຮູ້ = 9998*** | 1 = ແມ່ນ<br>0 = ບໍ່ແມ່ນ<br>8 = ບໍ່ຮູ້<br><br>[___] |

| 12. **ລາຄາໃນການຂາຍຍ່ອຍ** | 13. **ລາຄາຊື້ຍົກ (ຄັ້ງລ້າສຸດ)** | 14. **ເປັນຫຍັງທ່ານຈຶ່ງມີຢານີ້ [ສະແດງຜະລິດຕະພັນ]?** | 15. **ຄຳເຫັນອື່ນໆ** |
|---|---|---|---|
| ຈຳນວນ<br><br>[___|___|___]<br>**ຂວດແກ້ວ ຫຼື ຫຼອດແກ້ວສຳລັບຢາສັກ**<br><br>ລູກຄ້າແຕ່ລະຄົນຕ້ອງຈ່າຍ<br><br>[___|___|___|___|___]___] ກີບ | ຈຳນວນ<br><br>[___|___|___|___|___]<br>**ຂວດແກ້ວ ຫຼື ຫຼອດແກ້ວສຳລັບຢາສັກ**<br><br>ແມ່ນລາຄາ<br><br>[___|___|___|___|___|___]___] ກີບ | *ບໍ່ຕ້ອງອ່ານລາຍການ*<br>*ຂີດວົງອ້ອມເອົາທຸກຄຳຕອບທີ່ຜູ້ຕອບກ່າວເຖິງ*<br><br>ໄດ້ຮັບຢາລ້າ A<br>ມີກຳໄລ B<br>ຖືກແນະນຳໂດຍພາກລັດ C<br>ລາຄາຖືກ D<br>ຄວາມຕ້ອງການ ຫຼື ຄວາມນິຍົມຂອງລູກຄ້າ E<br>ຊື່ສຽງທາງບວກຂອງເຄື່ອງໝາຍສິນຄ້າ F<br>ໄດ້ຮັບການສັ່ງໂດຍທ່ານໝໍເລື້ອຍໆ G<br>ໄດ້ຮັບຜົນດີທີ່ສຸດສຳລັບການປົວໄຂ້ມາເລເລຍ H<br>ບໍ່ຮູ້ X<br>ອື່ນໆ Z<br>**ອະທິບາຍລະອຽດ**<br>[___________________________] | |
| *ບໍ່ເສຍຄ່າ = 000000*<br>*ປະຕິເສດ = 999997*<br>*ບໍ່ຮູ້ = 999998* | *ບໍ່ເສຍຄ່າ = 000000*<br>*ປະຕິເສດ = 999997*<br>*ບໍ່ຮູ້ = 999998* | | |

ເຈ້ຍກວດສອບຢາທີ່ບໍ່ແມ່ນຊະນິດເມັດ **(NT)** [___|___] ຂອງ [___|___]

| | |
|---|---|
| A16_1. ປະຈຸບັນທ່ານມີຢາຕ້ານເຊື້ອໃດໆບໍ່ ຢູ່ໃນຮ້ານນີ້ບໍ?<br>1 = ແມ່ນ<br>0 = ບໍ່ແມ່ນ ***ໄປຂໍ້ A17*** | [___] |
| A16_2. ທ່ານມີຢາດ໋ອກຊີ້ຊີຄລິນ (doxycycline) ເປັນເມັດ/ແຄັບຊູບໃດໆບໍ່ ຢູ່ໃນຮ້ານນີ້ບໍ?<br>*ເອົາບັດສະແດງໃຫ້ເບິ່ງ. ນັກສຳພາດ: ຂໍອະນຸຍາດເບິ່ງຜະລິດຕະພັນ ແລະ ຢັ້ງຢືນວ່າມັນແມ່ນ doxycycline (ດ໋ອກຊີ້ຊີຄລິນ)*<br>1 = ແມ່ນ<br>0 = ບໍ່ແມ່ນ ***ໄປຂໍ້ A17*** | [___] |
| A16_3. ຄວາມແຮງເທົ່າໃດຂອງຢາ doxycycline (ດ໋ອກຊີ້ຊີຄລິນ) ໃນໜຶ່ງເມັດ/ແຄັບຊູນ ທີທ່ານມີໃນຮ້ານນີ້ບໍ?<br>*ອ່ານລາຍການ, ວົງມົນເອົາຄຳຕອບທີ່ກ່າວເຖິງ* | |
| 100 mg | A |
| ບໍ່ຮູ້ | X |
| ອື່ນໆ (ລະບຸ): [_______________________________] | Z |

ລະຫັດຮ້ານຈຳໜ່າຍ: [___|___]-[___|___]-[___|___|___]- [___|___|___]

## ຢາປິ່ນປົວພະຍາດໄຂ້ມາເລເລຍທີ່ບໍ່ມີຢູ່ໃນຮ້ານ (ຂາດສາງ)

| | |
|---|---|
| A17. ປະຈຸບັນມີຢາປິ່ນປົວພະຍາດໄຂ້ມາເລເລຍທີ່ບໍ່ມີຢູ່ໃນຮ້ານ (ຂາດສາງ) ບໍ່, ແຕ່ທ່ານເຄີຍມີ ໃນ <u>3 ເດືອນ</u> ຜ່ານມາ ?<br>1 = ມີ *ໄປຂໍ້ A18*<br>0 = ບໍ່ມີ *ໄປຂໍ້ A20*<br>8 = ບໍ່ຮູ້ *ໄປຂໍ້ A20* | [___] |
| A18. ທ່ານຮູ້ຈັກຊື່ຢາປິ່ນປົວພະຍາດໄຂ້ມາເລເລຍທີ່ບໍ່ມີຢູ່ໃນຮ້ານ (ຂາດສາງ) ບໍ່?<br>1 = *ຮູ້ຈັກ*<br>0 = *ບໍ່, ຜູ້ໃຫ້ບໍລິການບໍ່ສາມາດຈື່ໄດ້* *ໄປຂໍ້ A20* | [___] |

A17a. ຊື່ ແລະ ສູດສະແດງຂອງຢາປິ່ນປົວພະຍາດໄຂ້ມາເລເລຍທີ່ບໍ່ມີຢູ່ໃນຮ້ານ (ຂາດສາງ) ນັ້ນແມ່ນຫຍັງ?
*ໃຫ້ເບິ່ງບັນຊີລາຍຊື່ການຄ້າດັ່ງລຸ່ມນີ້ ເພື່ອຕື່ມຄຳຕອບໃນຂໍ້ທີ* 4

ລາຍການ ຢາປິ່ນປົວໄຂ້ມາເລເລຍຊະນິດດຽວ ອາເຕີມີຊີນິນ (Oral AMT)

| ***Artesunate*** | | ***Artemether*** | ***Dihydroartemisinin*** |
|---|---|---|---|
| ***AA-Artesunate(tab)*** | ***Falcinate*** | ***Atem*** | ***Alaxin*** |
| ***Artesuate (tab)*** | ***ricn*** | ***A-Artmthr*** | ***Codisin*** |
| ***ArthsisAinae*** | ***Gsunae*** | ***meher*** | ***Temexin*** |
| ***Actitesunate*** | ***Lever-Artesunate*** | ***Artemedine*** | ***Santecxin*** |
| ***Adamsnate*** | ***MD-Artesunat*** | ***Betamotil*** | |
| ***Artemed*** | ***Pedisunate*** | | |
| ***Askasunate*** | ***Traphasunat*** | | |
| ***Cusnat-Artesunate*** | ***Vatunate*** | | |
| ***Artesunat*** | | | |
| ***Eurosunate*** | | | |

**ໝາຍເລກ ຢາຂາດສາງ**

[___|___]

**1. ຊື່ຕົວຢາ ຫຼື ຊື່ຍີ່ຫໍ້**

[______________________]

98 = ບໍ່ຮູ້

**2. ຮູບແບບຢາ/ສູດສະແດງ**

01 = ຢາເມັດ
02 = ຢາຍັດ
03 = ຢາຝຸ່ນຝຸ່ນເປັນຊອງ
04 = ນ້ຳເຊື່ອມ
05 = ຢານ້ຳແຂວນຕະກອນ
06 = ຢາສັກ (ນ້ຳ ຫຼື ຝຸ່ນ)
07 = ຢາຢອດ
98 = ບໍ່ຮູ້

[___|___]

**3. ທ່ານມີແຜນທີ່ຈະຊື້ຢານີ້ຕື່ມອີກບໍ່?**

1 = ແມ່ນ
0 = ບໍ່ແມ່ນ [___]
8 = ບໍ່ຮູ້

*<u>ບໍ່ຕ້ອງຖາມຜູ້ໃຫ້ບໍລິການ.</u>*

**4. ຢານີ້ມີຊື່ການຄ້າ/ຊື່ຍີ່ຫໍ້ ລວມຢູ່ໃນ ລາຍການຢາກິນປິ່ນປົວໄຂ້ມາເລເລຍ ຊະນິດດ່ຽວອາເຕີມີຊີນິນ (Oral AMT) ຂ້າງເທິງ ນັ້ນ ບໍ່?**

| | |
|---|---|
| 1 = ແມ່ນ 0 = ບໍ່ແມ່ນ<br>8 = ບໍ່ຮູ້ | [___] |

*ຖ້າວ່າ "ແມ່ນ", ໃຫ້ຕື່ມພາກ* ຢາກິນປິ່ນປົວໄຂ້ມາເລເລຍ ຊະນິດດ່ຽວອາເຕີມີຊີນິນ (*Oral AMT)* ຫຼັງຈາກທີ່ພາກ *"ຢາປິ່ນປົວພະຍາດໄຂ້ມາເລເລຍ ທີ່ຂາດສາງ"* ໄດ້ຖືກຕື່ມຄົບແລ້ວ.

ຖ້າວ່າ "ບໍ່ແມ່ນ" ໄປຂໍ້ 5

**5. ຢານີ້ແມ່ນເປັນຢາເມັດບໍ່?**

| | |
|---|---|
| 1 = ແມ່ນ 0 = ບໍ່ແມ່ນ<br>8 = ບໍ່ຮູ້ | [___] |

ຖ້າ "ບໍ່ແມ່ນ" ຫຼື "ບໍ່ຮູ້" ໃຫ້ໄປຕໍ່ທີ່ຢາອື່ນ

**6. ຢານີ້ມີສານອອກລິດພຽງຕົວດຽວບໍ່?**

| | |
|---|---|
| 1 = ແມ່ນ 0 = ບໍ່<br>8 = ບໍ່ຮູ້ | [___] |

ຖ້າ "ບໍ່ແມ່ນ" ຫຼື "ບໍ່ຮູ້" ໃຫ້ໄປຕໍ່ທີ່ຢາອື່ນ.

**7. ສານອອກລິດນີ້ແມ່ນໜຶ່ງໃນສານຕໍ່ໄປນີ້ບໍ່?**

- ອາເຕເມເທີ (ARTEMETHER)
- ອາເຕຊູເນັດ (ARTESUNATE)
- ດີຮາຍໂດອາເຕມີຊີນິນ (DIHYDROARTEMISININ)

| | |
|---|---|
| 1 = ແມ່ນ<br>0 = ບໍ່ແມ່ນ | [___] |

*ຖ້າວ່າ "ແມ່ນ", ໃຫ້ຕື່ມພາກ* ຢາກິນປິ່ນປົວໄຂ້ມາເລເລຍ ຊະນິດດ່ຽວອາເຕີມີຊີນິນ *(Oral AMT)* ຫຼັງຈາກທີ່ພາກ *"ຢາປິ່ນປົວພະຍາດໄຂ້ມາເລເລຍ ທີ່ຂາດສາງ"* ໄດ້ຖືກຕື່ມຄົບແລ້ວ

ລະຫັດຮ້ານຈຳໜ່າຍ: [___|___]-[___|___]-[___|___|___]- [___|___|___]

A17a. ຊື່ ແລະ ສູດສະແດງຂອງຢາປິ່ນປົວພະຍາດໄຂ້ມາເລເລຍທີ່ບໍ່ມີຢູ່ໃນຮ້ານ (ຂາດສາງ) ນັ້ນແມ່ນຫຍັງ?

**ໃຫ້ເບິ່ງບັນຊີລາຍຊື່ການຄ້າດັ່ງລຸ່ມນີ້ ເມື່ອຕື່ມຄຳຕອບໃນຂໍ້ທີ 4**

ລາຍການ ຢາປິ່ນປົວໄຂ້ມາເລເລຍຊະນິດກິນ ອາເຕີມີຊີນິນ (Oral AMT)

| ***Artesunate*** | | ***Artemether*** | ***Dihydroartemisinin*** |
|---|---|---|---|
| ***AA-Artesunate(tab)*** | ***Falcinate*** | ***Atem*** | ***Alaxin*** |
| ***Artesuate (tab)*** | ***ricn*** | ***A-Artmthr*** | ***Codisin*** |
| ***ArthsisAinae*** | ***Gsunae*** | ***meher*** | ***Temexin*** |
| ***Actitesunate*** | ***Lever-Artesunate*** | ***Artemedine*** | ***Santecxin*** |
| ***Adamsnate*** | ***MD-Artesunat*** | ***Betamotil*** | |
| ***Artemed*** | ***Pedisunate*** | | |
| ***Askasunate*** | ***Traphasunat*** | | |
| ***Cusnat-Artesunate*** | ***Vatunate*** | | |
| ***Artesunat*** | | | |
| ***Eurosunate*** | | | |

| | | | |
|---|---|---|---|
| ໝາຍເລກ ຢາຂາດສາງ<br>[___\|___] | 1. ຊື່ຕົວຢາ ຫຼື ຊື່ຍີ່ຫໍ້<br>[________________________]<br>98 = ບໍ່ຮູ້ | *<u>ບໍ່ຕ້ອງຖາມຜູ້ໃຫ້ບໍລິການ.</u>*<br>4. ຢານີ້ມີຊື່ການຄ້າ/ຊື່ຍີ່ຫໍ້ ລວມຢູ່ໃນ ລາຍການຢາກິນປິ່ນປົວໄຂ້ມາເລເລຍ ຊະນິດກິນອາເຕີມີຊີນິນ (Oral AMT) ຂ້າງເທິງ ນັ້ນບໍ່?<br>1 = ແມ່ນ 0 = ບໍ່ແມ່ນ 8 = ບໍ່ຮູ້ [___]<br>*ຖ້າວ່າ "ແມ່ນ", ໃຫ້ຕື່ມພາກ* ຢາກິນປິ່ນປົວໄຂ້ມາເລເລຍ ຊະນິດກິນອາເຕີມີຊີນິນ (*Oral AMT)* ຫຼັງຈາກທີ່ພາກ *"ຢາປິ່ນປົວພະຍາດໄຂ້ມາເລເລຍ ທີ່ຂາດສາງ"* ໄດ້ຖືກຕື່ມຄົບແລ້ວ.<br>ຖ້າວ່າ "ບໍ່ແມ່ນ" ໄປຂໍ້ 5<br>5. ຢານີ້ແມ່ນເປັນຢາເມັດບໍ່?<br>1 = ແມ່ນ 0 = ບໍ່ແມ່ນ 8 = ບໍ່ຮູ້ [___]<br>ຖ້າ "ບໍ່ແມ່ນ" ຫຼື "ບໍ່ຮູ້" ໃຫ້ໄປຕໍ່ທີ່ຢາອື່ນ | 6. ຢານີ້ມີສານອອກລິດພຽງຕົວດຽວບໍ່?<br>1 = ແມ່ນ 0 = ບໍ່ 8 = ບໍ່ຮູ້ [___]<br>ຖ້າ "ບໍ່ແມ່ນ" ຫຼື "ບໍ່ຮູ້" ໃຫ້ໄປຕໍ່ທີ່ຢາອື່ນ.<br>7. ສານອອກລິດນີ້ແມ່ນໜຶ່ງໃນສານຕໍ່ໄປນີ້ບໍ່?<br>• ອາເຕເມເທີ (ARTEMETHER)<br>• ອາເຕຊູເນັດ (ARTESUNATE)<br>• ດີຮາຍໂດອາເຕມີຊີນິນ (DIHYDROARTEMISININ)<br>1 = ແມ່ນ 0 = ບໍ່ແມ່ນ [___]<br>*ຖ້າວ່າ "ແມ່ນ", ໃຫ້ຕື່ມພາກ* ຢາກິນປິ່ນປົວໄຂ້ມາເລເລຍ ຊະນິດກິນອາເຕີມີຊີນິນ *(Oral AMT)* ຫຼັງຈາກທີ່ພາກ *"ຢາປິ່ນປົວພະຍາດໄຂ້ມາເລເລຍ ທີ່ຂາດສາງ"* ໄດ້ຖືກຕື່ມຄົບແລ້ວ |
| 2. ຮູບແບບຢາ/ສູດສະແດງ<br>01 = ຢາເມັດ<br>02 = ຢາຍັດ<br>03 = ຢາຝຸ່ນຝຸ່ນເປັນຊອງ<br>04 = ນ້ຳເຊື່ອມ<br>05 = ຢານ້ຳແຂວນຕະກອນ<br>06 = ຢາສັກ (ນ້ຳ ຫຼື ຝຸ່ນ)<br>07 = ຢາຢອດ<br>98 = ບໍ່ຮູ້<br>[___\|___] | | | |
| 3. ທ່ານມີແຜນທີ່ຈະຊື້ຢານີ້ຕື່ມອີກບໍ່?<br>1 = ແມ່ນ<br>0 = ບໍ່ແມ່ນ [___]<br>8 = ບໍ່ຮູ້ | | | |

A17a. ຊື່ ແລະ ສູດສະແດງຂອງຢາປິ່ນປົວພະຍາດໄຂ້ມາເລເລຍທີ່ບໍ່ມີຢູ່ໃນຮ້ານ (ຂາດສາງ) ນັ້ນແມ່ນຫຍັງ?
*ໃຫ້ເບິ່ງບັນຊີລາຍຊື່ການຄ້າດັ່ງລຸ່ມນີ້ ເມື່ອຕື່ມຄຳຕອບໃນຂໍ້ທີ* 4

ລາຍການ ຢາປິ່ນປົວໄຂ້ມາເລເລຍຊະນິດດຽວ ອາເຕີມີຊີນິນ (Oral AMT)

| ***Artesunate*** | | ***Artemether*** | ***Dihydroartemisinin*** |
|---|---|---|---|
| ***AA-Artesunate(tab)*** | ***Falcinate*** | ***Atem*** | ***Alaxin*** |
| ***Artesuate (tab)*** | ***ricn*** | ***A-Artmthr*** | ***Codisin*** |
| ***ArthsisAinae*** | ***Gsunae*** | ***meher*** | ***Temexin*** |
| ***Actitesunate*** | ***Lever-Artesunate*** | ***Artemedine*** | ***Santecxin*** |
| ***Adamsnate*** | ***MD-Artesunat*** | ***Betamotil*** | |
| ***Artemed*** | ***Pedisunate*** | | |
| ***Askasunate*** | ***Traphasunat*** | | |
| ***Cusnat-Artesunate*** | ***Vatunate*** | | |
| ***Artesunat*** | | | |
| ***Eurosunate*** | | | |

ໝາຍເລກ ຢາຂາດສາງ

[___|___]

1. ຊື່ຕົວຢາ ຫຼື ຊື່ຍີ່ຫໍ້

[______________________]

98 = ບໍ່ຮູ້

2. ຮູບແບບຢາ/ສູດສະແດງ

01 = ຢາເມັດ
02 = ຢາຍັດ
03 = ຢາຝຸ່ນຝຸ່ນເປັນຊອງ
04 = ນ້ຳເຊື່ອມ
05 = ຢານ້ຳແຂວນຕະກອນ
06 = ຢາສັກ (ນ້ຳ ຫຼື ຝຸ່ນ)
07 = ຢາຢອດ
98 = ບໍ່ຮູ້

[___|___]

3. ທ່ານມີແຜນທີ່ຈະຊື້ຢານີ້ຕື່ມອີກບໍ່?

1 = ແມ່ນ
0 = ບໍ່ແມ່ນ [___]
8 = ບໍ່ຮູ້

*ບໍ່ຕ້ອງຖາມຜູ້ໃຫ້ບໍລິການ.*

4. ຢານີ້ມີຊື່ການຄ້າ/ຊື່ຍີ່ຫໍ້ ລວມຢູ່ໃນ ລາຍການຢາກິນປິ່ນປົວໄຂ້ມາເລເລຍ ຊະນິດດ່ຽວ ອາເຕີມີຊີນິນ (Oral AMT) ຂ້າງເທິງ ນັ້ນບໍ່?

| | |
|---|---|
| 1 = ແມ່ນ 0 = ບໍ່ແມ່ນ<br>8 = ບໍ່ຮູ້ | [___] |

*ຖ້າວ່າ "ແມ່ນ", ໃຫ້ຕື່ມພາກ* ຢາກິນປິ່ນປົວໄຂ້ມາເລເລຍ ຊະນິດດ່ຽວອາເຕີມີຊີນິນ (*Oral AMT) ຫຼັງ*ຈາກທີ່ພາກ *"ຢາປິ່ນປົວພະຍາດໄຂ້ມາເລເລຍ ທີ່ຂາດສາງ"* ໄດ້ຖືກຕື່ມຄົບແລ້ວ.

ຖ້າວ່າ "ບໍ່ແມ່ນ" ໄປຂໍ້ 5

5. ຢານີ້ແມ່ນເປັນຢາເມັດບໍ່?

| | |
|---|---|
| 1 = ແມ່ນ 0 = ບໍ່ແມ່ນ<br>8 = ບໍ່ຮູ້ | [___] |

ຖ້າ "ບໍ່ແມ່ນ" ຫຼື "ບໍ່ຮູ້" ໃຫ້ໄປຕໍ່ທີ່ຢາອື່ນ

6. ຢານີ້ມີສານອອກລິດພຽງຕົວດຽວບໍ່?

| | |
|---|---|
| 1 = ແມ່ນ 0 = ບໍ່<br>8 = ບໍ່ຮູ້ | [___] |

ຖ້າ "ບໍ່ແມ່ນ" ຫຼື "ບໍ່ຮູ້" ໃຫ້ໄປຕໍ່ທີ່ຢາອື່ນ.

7. ສານອອກລິດນີ້ແມ່ນໜຶ່ງໃນສານຕໍ່ໄປນີ້ບໍ່?

- ອາເຕເມເທີ (ARTEMETHER)
- ອາເຕຊູເນັດ (ARTESUNATE)
- ດີຮາຍໂດອາເຕມີຊີນິນ (DIHYDROARTEMISININ)

| | |
|---|---|
| 1 = ແມ່ນ<br>0 = ບໍ່ແມ່ນ | [___] |

*ຖ້າວ່າ "ແມ່ນ", ໃຫ້ຕື່ມພາກ* ຢາກິນປິ່ນປົວໄຂ້ມາເລເລຍ ຊະນິດດ່ຽວອາເຕີມີຊີນິນ *(Oral AMT)* ຫຼັງຈາກທີ່ພາກ *"ຢາປິ່ນປົວພະຍາດໄຂ້ມາເລເລຍ ທີ່ຂາດສາງ"* ໄດ້ຖືກຕື່ມຄົບແລ້ວ

ລະຫັດຮ້ານຈຳໜ່າຍ: [___|___]-[___|___]-[___|___|___]- [___|___|___]

| | |
|---|---|
| A20. ***ບໍ່ຕ້ອງອ່ານ***. *ພົບຢາກິນປິ່ນປົວໄຂ້ຍຸງຊະນິດຖ່ງອອາເຕີຊີນີນ (Oral AMT) ໃນພາກກວດສອບຢາປິ່ນປົວໄຂ້ມາເລເລຍ ຫຼື ໃນຄຳຖາມຂໍ້ A17a (Stock out) ບໍ່?*<br>1 = ແມ່ນ ***ຕື່ມພາກ ຢາກິນປິ່ນປົວໄຂ້ຍຸງຊະນິດຖ່ງອອາເຕີຊີນີນ (Oral AMT) ຊະນິດກິນ ສຳລັບຢາກິນປິ່ນປົວໄຂ້ຍຸງຊະນິດຖ່ງອອາເຕີຊີນີນ ແຕ່ລະອັນທີ່ມີ.***<br>***ພາກ ຢາກິນປິ່ນປົວໄຂ້ຍຸງຊະນິດຖ່ງອອາເຕີມີຊີນີນ (Oral AMT) ຈະຖືກຕື່ມສຳລັບ:***<br>***1) ຜະລິດຕະພັນ ຢາເມັດ, ຢາຢັດ, ຢາຝຸ່ນເປັນຊອງ (TSG) ທີ່ໄດ້ຖືກກວດສອບທີ່ມີຄຳຕອບແມ່ນ 1 ສຳລັບຄຳຖາມ 16a, 16b and 16c.***<br>***2) ຜະລິດຕະພັນທັງໝົດທີ່ບໍ່ມີໃນຮ້ານ (ຂາດສາງ) ໃນປະຈຸບັນ ທີ່ມີຄຳຕອບແມ່ນເລກ 1 ໃນຄຳຖາມຂໍ້ A17a , ຂໍ້ທີ 4 ແລະ ຂໍ້ທີ 7.***<br>0 = ບໍ່ແມ່ນ **ໄປພາກທີ 4: ກວດສອບການບົ່ງມະຕິ** | [___] |

**ພາກທີ 4: ການກວດສອບການບົ່ງມະຕິ**

ພາກນີ້ແມ່ນເບິ່ງກ່ຽວກັບການມີແນວກວດເລືອດຫາເຊື້ອໄຂ້ມາເລເລຍ . ການຕອບຄຳຖາມເຫຼົ່ານີ້ອາດຮຽກຮ້ອງໃຫ້ມີການລົມກັບພະນັກງານໃນຮ້ານຈຳໜ່າຍຫຼາຍກວ່າ 1 ຄົນ. ຖ້າຜູ້ຖືກສອບຖາມບໍ່ຮູ້ຄຳຕອບສຳລັບຄຳຖາມໃນພາກນີ້, ຂໍລົມກັບພະນັກງານຄົນອື່ນໆທີ່ສາມາດໃຫ້ຂໍ້ມູນໄດ້.

| | |
|---|---|
| D1. ໃນປະຈຸບັນ ຮ້ານຈຳໜ່າຍ/ສະຖານທີ່ບໍລິການແຫ່ງນີ້ ມີຖົງມືທີ່ໃຊ້ຄັ້ງດຽວແລ້ວຖິ້ມ ສຳລັບພະນັກງານເພື່ອໃຊ້ໃນການກວດລູກຄ້າ/ຄົນເຈັບບໍ່?<br>1 = ມີ<br>0 = ບໍ່ມີ<br>8 = ບໍ່ຮູ້ | [___] |
| D2. ຮ້ານຈຳໜ່າຍ/ສະຖານທີ່ບໍລິການແຫ່ງນີ້ ມີທີ່ເກັບມ້ຽນຂອງແຫຼມຄົມ, ຫຼື ທີ່ຖືກຮ້ອງວ່າ ກັບທີ່ຖິ້ມຂອງແຫຼມຄົມ ຫຼື ກັບນິລະໄພ, ທີ່ມີພ້ອມໃນປະຈຸບັນ ສຳລັບໃຫ້ພະນັກງານໄດ້ນຳໃຊ້ບໍ່?<br>1 = ມີ<br>0 = ບໍ່ມີ<br>8 = ບໍ່ຮູ້ | [___] |
| D3. ມື້ນີ້, ຢູ່ທີ່ນີ້ ມີການກວດຫາເຊື້ອໄຂ້ມາເລເລຍ ດ້ວຍກ້ອງຈຸລະທັດບໍ່?<br>1 = ມີ<br>0 = ບໍ່ມີ ***ໄປຂໍ້ D6a*** | [___] |
| D4. ມີຈັກຄົນທີ່ໄດ້ຮັບການກວດຫາ **ເຊື້ອໄຂ້ມາເລເລຍ** ທີ່ຮ້ານຈຳໜ່າຍ/ສະຖານທີ່ບໍລິການແຫ່ງນີ້ ດ້ວຍການໃຊ້ **ກ້ອງຈຸລະທັດ**ພາຍໃນ 7 ມື້ທີ່ຜ່ານມາ?<br>997 = *ປະຕິເສດ* ; 998 = *ບໍ່ຮູ້* | [___\|___\|___] |
| D5. ລາຄາທັງໝົດ ສຳລັບການກວດດ້ວຍກ້ອງຈຸລະທັດ ເພື່ອຊອກຫາເຊື້ອໄຂ້ມາເລເລຍ ສຳລັບຜູ້ໃຫຍ່ແມ່ນເທົ່າໃດ:<br>[___\|___\|___\|___\|___\|___] **ກີບ**<br>***ບໍ່ເສຍຄ່າ = 000000; ບໍ່ມີຂໍ້ມູນ = 999995; ປະຕິເສດ = 999997; ບໍ່ຮູ້ = 999998*** | |

| | |
|---|---|
| D6a. ການກວດຫາ ທາດ G6PD (ທາດ G6PD ແມ່ນທາດນ້ຳຕານໃນເມັດເລືອດແດງ) ແມ່ນໄດ້ຖືກນຳໃຊ້ໃນການກຳນົດວ່າແຕ່ລະຄົນມີການຂາດ G6PD. ຢາປິ່ນປົວພະຍາດໄຂ້ມາເລເລຍ ທີ່ສະເພາະເຈາະຈົງສາມາດເປັນອັນຕະລາຍຕໍ່ຄົນເຈັບທີ່ຂາດ G6PD.<br>**ທ່ານເຄີຍໄດ້ຍິນກ່ຽວກັບການກວດ G6PD ບໍ່?**<br>1 = ແມ່ນ<br>0 = ບໍ່ແມ່ນ ***ໄປຂໍ້ D7*** | [___] |
| D6b. ຮ້ານຈຳໜ່າຍແຫ່ງນີ້ ໄດ້ກວດຊອກຫາທາດG6PD (ທາດ G6PD ແມ່ນທາດນ້ຳຕານໃນເມັດເລືອດແດງ) ໃນຄົນທົ່ວໄປບໍ່?<br>1 = ແມ່ນ<br>0 = ບໍ່ແມ່ນ ***ໄປທີ່ D7***<br>ບໍ່ຮູ້ **ຂໍລົມກັບຜູ້ທີ່ມີຂໍ້ມູນກ່ຽວກັບເລື້ອງນີ້** | [___] |
| D6c. ຮ້ານຈຳໜ່າຍແຫ່ງນີ້ໄດ້ນຳໃຊ້ການກວດດ້ວຍເຈ້ຍຈຸ່ມບົ່ງມະຕິແບບວ່ອງໄວ ສຳລັບການກວດຊອກຫາທາດ G6PD (ທາດ G6PD ແມ່ນທາດນ້ຳຕານໃນເມັດເລືອດແດງ) ບໍ່?<br>1 = ແມ່ນ<br>0 = ບໍ່ແມ່ນ ***ໄປຂໍ້ D7***<br>ບໍ່ຮູ້ **ຂໍລົມກັບຜູ້ທີ່ມີຂໍ້ມູນກ່ຽວກັບເລື້ອງນີ້** | [___] |
| D6d. ຮ້ານຈຳໜ່າຍແຫ່ງນີ້ ໃຊ້ວິທີການກວດດ້ວຍເຈ້ຍຈຸ່ມບົ່ງມະຕິແບບວ່ອງໄວ ແບບໃດເພື່ອກວດຫາ G6PD?<br>**ໃຫ້ຜູ້ບໍລິການລວບລວມເອົາແນວກວດບົ່ງມະຕິແບບວ່ອງໄວສຳລັບກວດກາ G6PDມາໃຫ້ເບິ່ງ**<br><br>**<u>ບໍ່ຕ້ອງອ່ານບັນຊີລາຍຊື່ລຸ່ມນີ້.</u> ຊີດວົງອ້ອມເອົາແນວກວດບົ່ງມະຕິແບບວ່ອງໄວສຳລັບກວດ**ຫາທາດG6PD (ທາດ G6PD ແມ່ນທາດນ້ຳຕານໃນເມັດເລືອດແດງ) **ທັງໝົດທີ່ໄດ້ສັງເກດເຫັນວ່າມີໃນຮ້ານ.**<br><br>**ບັນທຶກຊື່ການຄ້າ ແລະ ຜູ້ຜະລິດສຳລັບແນວກວດບົ່ງມະຕິແບບວ່ອງໄວສຳລັບກວດຫາທາດG6PD ທີ່ສັງເກດວ່າບໍ່ໄດ້ຢູ່ໃນບັນຊີລາຍຊື່ລຸ່ມນີ້** | |
| ເຈ້ຍຈຸ່ມບົ່ງມະຕິແບບວ່ອງໄວ Carestart G6PDT ຜະລິດໂດຍ Access Bio | A |
| ເຈ້ຍຈຸ່ມບົ່ງມະຕິແບບວ່ອງໄວ BinaxNOW G6PD ຜະລິດໂດຍ Alere | B |
| ເຈ້ຍຈຸ່ມບົ່ງມະຕິແບບວ່ອງໄວ G-6-PDH Dye Reduction G6PD ຜະລິດໂດຍ Trinity Biotech | C |
| ອື່ນໆ (ອະທິບາຍລະອຽດຊື່ການຄ້າ ແລະ ຜູ້ຜະລິດ) [______________________________] | X |
| D6e. ຮ້ານຈຳໜ່າຍແຫ່ງນີ້ ໄດ້ກວດດ້ວຍເຈ້ຍຈຸ່ມບົ່ງມະຕິແບບວ່ອງໄວ ສຳລັບກວດຫາທາດ G6PD (ທາດ G6PD ແມ່ນທາດນ້ຳຕານໃນເມັດເລືອດແດງ) ໃຫ້ກັບຈັກຄົນ ໃນເດືອນຜ່ານມາ?<br>***997 = ປະຕິເສດ, 998 = ບໍ່ຮູ້*** | [___\|___\|___] |
| D7. ການກວດດ້ວຍເຈ້ຍຈຸ່ມບົ່ງມະຕິແບບວ່ອງໄວ ເພື່ອຊອກຫາເຊື້ອໄຂ້ມາເລເລຍ , ຫຼືຖືກເອີ້ນວ່າ ການກວດບົ່ງມະຕິແບບວ່ອງໄວ, ມັນມີຂະໜາດນ້ອຍ, ໃສ່ໃນຊອງຂອງໃຜມັນຊຶ່ງຈະຖືກໃຊ້ບົ່ງມະຕິແບບວ່ອງໄວວ່າຄົນຜູ້ນັ້ນຕິດເຊື້ອໄຂ້ມາເລເລຍ ຫຼື ບໍ່. **ເອົາບັດສະແດງຮູບຂອງແນວກວດບົ່ງມະຕິແບບວ່ອງໄວໃຫ້ເບິ່ງ**<br><br>**ມື້ນີ້, ຢູ່ທີ່ນີ້ ມີເຈ້ຍຈຸ່ມ**ກວດ**ບົ່ງມະຕິແບບວ່ອງໄວຫາເຊື້ອໄຂ້ມາເລເລຍບໍ່?**<br>1 = ມີ<br>0 = ບໍ່ມີ ***ໄປຂໍ້ D9***<br>ບໍ່ຮູ້ ***ຂໍລົມກັບຜູ້ທີ່ມີຂໍ້ມູນນີ*** | [___] |

| |
|---|
| D8. ກະລຸນາເອົາເຈ້ຍຈຸ່ມບົ່ງມະຕິແບບວ່ອງໄວ ທີ່ທ່ານມີຢູ່ໃນຮ້ານໃນປະຈຸບັນນີ້ໃຫ້ພວກເຮົາເບິ່ງແດ່. ປະຈຸບັນນີ້, ທ່ານມີອັນໃດອັນໜຶ່ງດັ່ງຕໍ່ໄປນີ້ບໍ່?<br>**ອ່ານບັນຊີລາຍຊື່ທັງໝົດຂ້າງລຸ່ມນີ້; *ບໍ່ຕ້ອງບັນທຶກຫຍັງທັງສິ້ນໃນຂໍ້ນີ້***<br>• ແອກຄິວເຣດ *Accurate, ແຄສະຕາດ Carestart, ຄູ້ນຈີນ Clungene, ເຟີດສ໌ເຣດສ໌ປອນ First Response, ມາລາເຊັກ Malacheck, ວັນສະເຕັບ One Step, ພາຣາຮິດ ParaHit, ພາຣາເຊັກ Paracheck, ເອດສ໌ດີ ໄບໂອລາຍ SD Bioloine, ຮິວມາຊີສ໌ Humasis, ໄດອາແລັບ Dialab , ຄອມໂບ ອາດີທີ Combo-RDT* |

***ສືບຕໍ່ການກວດສອບ ເຈ້ຍຈຸ່ມບົ່ງມະຕິແບບວ່ອງໄວ***

***ຖ້າໄດ້ໃຊ້ແບບຟອມການກວດສອບເພີ່ມຕື່ມ, ເພີ່ມແບບຟອມເຫຼົ່ານີ້ທັງຟອມທີ່ໄດ້ສະໜອງໃຫ້ ແລະ ໜີບກະລັບເຟີ້ລວມກັນ. ທຸກໜ້າເຈ້ຍຄວນໄດ້ຮັບການຈັດລຽງກ່ອນທີ່ທ່ານຈະຍ້າຍໄປທີ່ຮ້ານຕໍ່ໄປ.***

***ໃຫ້ໝາຍເລກ ເຈ້ຍຈຸ່ມບົ່ງມະຕິແບບວ່ອງໄວ ແຕ່ລະອັນໂດຍລະບຸໝາຍເລກຜະລິດຕະພັນ.***

***ໃຫ້ໝາຍເລກແບບຟອມການກວດສອບແຕ່ລະອັນໃສ່ໃນບ່ອນວ່າງທີ່ຈັດກຽມໄວ້ດ້ານລຸ່ມຂອງໜ້າເຈ້ຍ***

***ໃນຮ້ານຈຳໜ່າຍທັງໝົດ, ຕື່ມ ລະຫັດໜ່ວຍງານຍ່ອຍ (ພ້ອມທັງໝາຍເລກຜະລິດຕະພັນ) ສຳລັບຢາແຕ່ລະຕົວທີ່ໄດ້ຖືກກວດສອບ. ລະຫັດໜ່ວຍງານຍ່ອຍ ເຫຼົ່ານີ້ແມ່ນຢູ່ຂ້າງລຸ່ມນີ້***

| **ລະຫັດໜ່ວຍງານຍ່ອຍ** | |
|---|---|
| X | ບັນດາຮ້ານຈຳໜ່າຍຕ່າງໆທີ່ມີ ຈຸດຈ່າຍ/ແຈກຢາຍຢາ ສຳລັບຢາປິ່ນປົວ/ການບົ່ງມະຕິ **ຈຸດດຽວເທົ່ານັ້ນ.** |
| A | ພະແນກກວດເຂດນອກ / ບ່ອນຈ່າຍຢາ/ ຮ້ານຂາຍຢາຫຼັກ ( (ທີ່ຄົນເຈັບທັງໝົດມາໃຊ້ບໍລິການ). |
| B | ພະແນກກວດເຂດນອກສຳລັບຜູ້ໃຫຍ່/ບ່ອນຈ່າຍຢາສຳລັບຜູ້ໃຫຍ່/ຄລີນິກຜູ້ໃຫຍ່ |
| C | ພະແນກກວດເຂດນອກສຳລັບເດັກ/ບ່ອນຈ່າຍຢາສຳລັບເດັກ/ຄລີນິກເດັກ |
| D | ການຝາກທ້ອງ / ຄລີນິກສຳລັບແມ່ /ສຸຂະພາບແມ່ ແລະ ເດັກ |
| E | ຄລີນິກບໍລິການປິ່ນປົວພະຍາດເອດສ໌ (ART) /ຄລີນິກ HIV/ ໂລກເອດສ໌AIDS |
| F | ພະແນກ ພະຍາດຕິດຕໍ່ |
| G | ພະແນກສຸກເສີນ |
| H | ພະແນກຈ່າຍຢາທີ່ເປັນຂອງເອກະຊົນ ທີ່ຕັ້ງຢູ່ໃນສະຖານທີ່ບໍລິການສາທາລະນະສຸກຂອງລັດ |
| I | ໜ່ວຍງານວິເຄາະ (*ສຳລັບການກວດບົ່ງມະຕິແບບວ່ອງໄວດ້ວຍເຈ້ຍຈຸ່ມ*) |
| Z | ອື່ນໆ (*ລະບຸລະອຽດກ່ຽວກັບປະເພດ ໃນບ່ອນວ່າງສຳລັບຫ້ອງຄຳເຫັນຢູ່ໃນເຈ້ຍກວດສອບເຈ້ຍກວດສອບ ຢາເມັດ, ຢາຍັດ, ຢາຝຸ່ນເປັນຊອງ (TSG) ຂໍ້ 15 ຫຼື ເຈ້ຍກວດສອບຢາທີ່ບໍ່ແມ່ນຊະນິດເມັດ* **(NT)** *ຂໍ້ 15)* |

(Black page)

เจ้ยกวดสอบเจ้ยจุ่มบิ่งมะติแบบว่องไว (RDT):

ละหัดร้านจำหน่าย: [___|___]-[___|___]-[___|___|___]-[___|___|___]

| ละหัด ໜ່ວยງານ ຍ່ອຍ<br>[______]<br>ໝາຍເລກ ຜະລິດຕະພັນ<br>[__\|__] | 1. ຊື່ການຄ້າ ຫຼື ຊື່ຍີ່ຫໍ້ | 2. ການກວດສານກໍ່ພູມຕ້ານທານ (ກວດຫາ antigen)<br>(ຂີດວົງອ້ອມເອົາທັງໝົດທີ່ເໝາະສົມ)<br>HRP2 A<br>pLDH B<br>Aldolase C<br>ບໍ່ລະບຸ Z | 3. ປະເພດແມ່ກາຝາກ<br>*(ຂີດວົງອ້ອມເອົາທັງໝົດທີ່ເໝາະສົມ)*<br>Pf A<br>Pv B<br>Po C<br>pm D<br>pan E<br>vom/Pvom F<br>ອື່ນໆ G<br>ລະບຸ [____________________]<br>ບໍ່ລະບຸ Z | 4. ຜູ້ຜະລິດ | 5.ປະເທດທີ່ຜະລິດ | 5b. ໝາຍເລກລະຫັດຜະລິດຕະພັນ (Catalogue Number) | 6. ໝາຍເລກໝວດ (Lot Number) | 7. ນີ້ແມ່ນຊຸດກວດດ້ວຍຕົວເອງບໍ່, ໃນແຕ່ລະຊຸດເຄື່ອງກວດປະກອບມີ ນ້ຳຢາ, ຫຼອດດູດເລືອດ ແລະ ແຜ່ນລາມ ມານຳບໍ່?<br>1 = ແມ່ນ<br>0 = ບໍ່ແມ່ນ<br>8 = ບໍ່ຮູ້<br>[___] |
|---|---|---|---|---|---|---|---|---|

| 13. ຈຳນວນຂອງຊຸດກວດທີ່ໄດ້ຈຳໜ່າຍໃຫ້ກັບລູກຄ້າລາຍຍ່ອຍ ໃນ 7 ມື້ທີ່ຜ່ານມາ<br>*(ບັນທຶກ ຈຳນວນ ທັງໝົດຂອງຊຸດກວດ)*<br>ໄດ້ຂາຍ/ແຈກຢາຍ<br>[___\|___\|___\|___]<br>ຊຸດກວດ ໃນ 7 ມື້ທີ່ຜ່ານມາ<br>ປະຕິເສດ*= 9997;*<br>ບໍ່ຮູ້ *= 9998* | 14. ເຄື່ອງກວດນີ້ ບໍ່ມີໃນຮ້ານ ໃນເວລາໃດໜຶ່ງ ໄລຍະ 3ເດືອນຜ່ານມາບໍ່?<br>1 = ແມ່ນ<br>0 = ບໍ່ແມ່ນ<br>8 = ບໍ່ຮູ້<br>[___] | 15a. ຮ້ານນີ້ນຳໃຊ້ ເຈ້ຍຈຸ່ມບົ່ງມະຕິແບບວ່ອງໄວຍີ່ຫໍ້ນີ້ ໃນການກວດລູກຄ້າ ທີ່ສະຖານທີ່ນີ້?<br>1 = ແມ່ນ<br>0 = ບໍ່ແມ່ນ ໄປຂໍ້ *16a*<br>8 = ບໍ່ຮູ້ ໄປຂໍ້ *16a*<br>[___]<br>15b. *ຖ້າແມ່ນ, ລາຄາການກວດທັງໝົດເທົ່າໃດ (ລວມທັງລາຄາຂອງ ຊຸດກວດ ແລະ ຄ່າບໍລິການ)*<br>[___\|___\|___\|___\|___]___]<br>ກີບ | 16a. ຮ້ານນີ້ເອົາເຈ້ຍຈຸ່ມບົ່ງມະຕິແບບວ່ອງໄວ ຍີ່ຫໍ້ນີ້ໃຫ້ ລູກຄ້າໄປກວດຢູ່ບ່ອນອື່ນບໍ່?<br>1 = ແມ່ນ<br>0 = ບໍ່ແມ່ນ ໄປຂໍ້ 17<br>8 = ບໍ່ຮູ້ ໄປຂໍ້ 17<br>[___]<br>16b. ຖ້າແມ່ນ, ຊຸດກວດດ້ວຍເຈ້ຍຈຸ່ມບົ່ງມະຕິແບບວ່ອງໄວນີ້ ມີລາຄາເທົ່າໃດ?<br>[___\|___\|___\|___\|___]___]<br>ກີບ | 17. ລາຄາໃນການຊື້ຍົກ (ຄັ້ງລ້າສຸດ)<br>ຈຳນວນຊື້ຍົກ<br>[___\|___\|___\|___] ຊຸດກວດ<br>ລາຄາ<br>[___\|___\|___\|___\|___\|___]___]<br>ກີບ<br>*ບໍ່ເສຍຄ່າ = 0000000*<br>*ບໍ່ໃສ່ຂໍ້ມູນ = 9999995*<br>*ປະຕິເສດ = 9999997*<br>*ບໍ່ຮູ້ = 9999998* | 18. ເປັນຫຍັງທ່ານຈຶ່ງມີ ແນວກວດດ້ວຍເຈ້ຍຈຸ່ມບົ່ງມະຕິແບບວ່ອງໄວນີ້ໄວ້ [ສະແດງ ແນວກວດບົ່ງມະຕິແບບວ່ອງໄວ]?<br>*ບໍ່ຕ້ອງອ່ານລາຍການ*<br>*ຂີດວົງອ້ອມເອົາທຸກຄຳຕອບທີ່ຜູ້ຕອບກ່າວເຖິງ*<br>ໄດ້ມາລ້າໆ A<br>ກຳໄລດີ **B**<br>ລັດຖະບານແນະນຳ C<br>ລາຄາຖືກ D<br>ລູກຄ້າຖາມມາ E<br>ມີຊື່ສຽງດີ F<br>ບໍ່ຮູ້ X<br>ອື່ນໆ Z<br>ລະບຸ[__________________] | 19. ຄຳເຫັນອື່ນໆ |
|---|---|---|---|---|---|---|
| | | *ບໍ່ເສຍຄ່າ = 000000; ປະຕິເສດ= 999997; ບໍ່ຮູ້=999998* | | | | |

ແບບກວດສອບຊຸດກວດບົ່ງມະຕິແບບວ່ອງໄວ [___\|]___] ຂອງ [___\|]___]

ເຈ້ຍກວດສອບເຈ້ຍຈຸ່ມບົ່ງມະຕິແບບວ່ອງໄວ **(RDT):** ລະຫັດຮ້ານຈຳໜ່າຍ: [___|___]-[___|___]-[___|___|___]-[___|___|___]

| ລະຫັດໜ່ວຍງານຍ່ອຍ | 1. ຊື່ການຄ້າ ຫຼື ຊື່ຍີ່ຫໍ້ | 2. ການກວດສານກໍ່ພູມຕ້ານທານ (ກວດຫາ antigen) | 3. ປະເພດແມ່ກາຝາກ | 4. ຜູ້ຜະລິດ | 5.ປະເທດທີ່ຜະລິດ | 5b. ໝາຍເລກລະຫັດຜະລິດຕະພັນ (Catalogue Number) | 6. ໝາຍເລກໝວດ (Lot Number) | 7. ນີ້ແມ່ນຊຸດກວດດ້ວຍຕົວເອງບໍ່, ໃນແຕ່ລະຊຸດເຄື່ອງກວດປະກອບມີ ນ້ຳຢາ, ຫຼອດດູດເລືອດ ແລະ ແຜ່ນລາມ ມານຳບໍ່? |
|---|---|---|---|---|---|---|---|---|
| [_____]<br><br>ໝາຍເລກຜະລິດຕະພັນ<br><br>[__\|__] | | (ຂີດວົງອ້ອມເອົາທັງໝົດທີ່ເໝາະສົມ)<br>HRP2 A<br>pLDH B<br>Aldolase C<br>ບໍ່ລະບຸ Z | *(ຂີດວົງອ້ອມເອົາທັງໝົດທີ່ເໝາະສົມ)*<br>Pf A<br>Pv B<br>Po C<br>pm D<br>pan E<br>vom/Pvom F<br>ອື່ນໆ G<br>ລະບຸ [_______________]<br>ບໍ່ລະບຸ Z | | | | | 1 = ແມ່ນ<br>0 = ບໍ່ແມ່ນ<br>8 = ບໍ່ຮູ້<br>[___] |

| 13. ຈຳນວນຂອງຊຸດກວດທີ່ໄດ້ຈຳໜ່າຍໃຫ້ກັບລູກຄ້າລາຍຍ່ອຍ ໃນ 7 ມື້ທີ່ຜ່ານມາ | 14. ເຄື່ອງກວດນີ້ ບໍ່ມີໃນຮ້ານ ໃນເວລາໃດໜຶ່ງ ໄລຍະ 3ເດືອນຜ່ານມາບໍ່? | 15a. ຮ້ານນີ້ນຳໃຊ້ ເຈ້ຍຈຸ່ມບົ່ງມະຕິແບບວ່ອງໄວຍີ່ຫໍ້ນີ້ ໃນການກວດລູກຄ້າ ທີ່ສະຖານທີ່ນີ້? | 16a. ຮ້ານນີ້ເອົາເຈ້ຍຈຸ່ມບົ່ງມະຕິແບບວ່ອງໄວ ຍີ່ຫໍ້ນີ້ໃຫ້ ລູກຄ້າໄປກວດຢູ່ບ່ອນອື່ນບໍ່? | 17. ລາຄາໃນການຊື້ຍົກ (ຄັ້ງລ້າສຸດ) | 18. ເປັນຫຍັງທ່ານຈຶ່ງມີ ແນວກວດດ້ວຍເຈ້ຍຈຸ່ມບົ່ງມະຕິແບບວ່ອງໄວນີ້ໄວ້ [ສະແດງ ແນວກວດບົ່ງມະຕິແບບວ່ອງໄວ]? | 19. ຄຳເຫັນອື່ນໆ |
|---|---|---|---|---|---|---|
| *(ບັນທຶກ ຈຳນວນ ທັງໝົດຂອງຊຸດກວດ)*<br><br>ໄດ້ຂາຍ/ແຈກຢາຍ [___\|___\|___\|___] ຊຸດກວດ ໃນ 7 ມື້ທີ່ຜ່ານມາ<br><br>ປະຕິເສດ*= 9997;*<br>ບໍ່ຮູ້ *= 9998* | 1 = ແມ່ນ<br>0 = ບໍ່ແມ່ນ<br>8 = ບໍ່ຮູ້<br><br>[___] | 1 = ແມ່ນ<br>0 = ບໍ່ແມ່ນ ໄປຂໍ້ *16a*<br>8 = ບໍ່ຮູ້ ໄປຂໍ້ *16a*<br><br>[___]<br><br>15b. *ຖ້າແມ່ນ, ລາຄາການກວດທັງໝົດເທົ່າໃດ (ລວມທັງລາຄາຂອງ ຊຸດກວດ ແລະ ຄ່າບໍລິການ)*<br>[___\|___\|___\|___\|___\|___]___] ກີບ | 1 = ແມ່ນ<br>0 = ບໍ່ແມ່ນ ໄປຂໍ້ 17<br>8 = ບໍ່ຮູ້ ໄປຂໍ້ 17<br><br>[___]<br><br>16b. ຖ້າແມ່ນ, ຊຸດກວດດ້ວຍເຈ້ຍຈຸ່ມບົ່ງມະຕິແບບວ່ອງໄວນີ້ ມີລາຄາເທົ່າໃດ?<br>[___\|___\|___\|___\|___\|___]___] ກີບ | ຈຳນວນຊື້ຍົກ<br>[___\|___\|___\|___]ຊຸດກວດ<br><br>ລາຄາ<br>[___\|___\|___\|___\|___\|___\|___]___] ກີບ<br><br>*ບໍ່ເສຍຄ່າ = 0000000*<br>*ບໍ່ໃສ່ຂໍ້ມູນ = 9999995*<br>*ປະຕິເສດ = 9999997*<br>*ບໍ່ຮູ້ = 9999998* | *ບໍ່ຕ້ອງອ່ານລາຍການ*<br>*ຂີດວົງອ້ອມເອົາທຸກຄຳຕອບທີ່ຜູ້ຕອບກ່າວເຖິງ*<br><br>ໄດ້ມາລ້າໆ A<br>ກຳໄລດີ B<br>ລັດຖະບານແນະນຳ C<br>ລາຄາຖືກ D<br>ລູກຄ້າຖາມມາ E<br>ມີຊື່ສຽງດີ F<br>ບໍ່ຮູ້ X<br>ອື່ນໆ Z<br>ລະບຸ[_______________] | |
| | | *ບໍ່ເສຍຄ່າ = 000000; ປະຕິເສດ= 999997; ບໍ່ຮູ້=999998* | | | | |

ແບບກວດສອບຊຸດກວດບົ່ງມະຕິແບບວ່ອງໄວ [___|]___] ຂອງ [___|]___]

**ເຈັຍຈຸ່ມບົ່ງມະຕິແບບວ່ອງໄວ ທີ່ບໍ່ມີໃນຮ້ານ (ຂາດສາງ)**

| | |
|---|---|
| D9. ໃນມື້ນີ້, ມີເຈັຍຈຸ່ມບົ່ງມະຕິແບບວ່ອງໄວ ສຳລັບກວດເຊື້ອໄຂ້ມາເລເລຍ ທີ່ບໍ່ມີຢູ່ໃນຮ້ານບໍ່, ແຕ່ວ່າເຄີຍມີຢູ່ໃນຮ້ານ ໃນ 3 ເດືອນຜ່ານມາ?<br>1 = ມີ<br>0 = ບໍ່ມີ ***ໄປຂໍ້ D11***<br>8 = ບໍ່ຮູ້ ***ໄປຂໍ້ D11*** | [___] |
| D9a. ຜູ້ໃຫ້ບໍລິການຮູ້ຊື່ຍີ່ຫໍ້ຂອງ ເຈັຍຈຸ່ມບົ່ງມະຕິແບບວ່ອງໄວ ທີ່ບໍ່ມີຢູ່ໃນຮ້ານບໍ່ ບໍ່?<br>1= ແມ່ນ<br>0= ບໍ່ແມ່ນ ***ໄປຂໍ້ D11*** | [___] |
| D10. ຊື່ການຄ້າ/ຊື່ຍີ່ຫໍ້ ຂອງເຈັຍຈຸ່ມບົ່ງມະຕິແບບວ່ອງໄວ ກວດເຊື້ອໄຂ້ມາເລເລຍ ທີ່ບໍ່ມີໃນຮ້ານນີ້ ມີຊື່ການຄ້າ/ຊື່ຍີ່ຫໍ້ວ່າແນວໃດ?<br>0 = ບໍ່ແມ່ນ/*ຜູ້ໃຫ້ບໍລິການບໍ່ຈື່* ***ໄປຂໍ້ D11***<br>1 = *ແມ່ນ,* ***ຂຽນລົງລະອຽດ*** **ບັນທຶກໜຶ່ງຍີ່ຫໍ້ຕໍ່ແຖວ**<br>[____________________]<br>[____________________]<br>[____________________] | [___] |
| D11. ສະຖານທີ່ບໍລິການ/ຮ້ານຈຳໜ່າຍແຫ່ງນີ້ໄດ້ໃຫ້ຈຳໜ່າຍ/ແຈກຢາຍຢາ ຫຼື ການອອກໃບສັ່ງຢາບໍ່?<br>1 = ແມ່ນ ***ໄປພາກທີ 5: ພາກຜູ້ໃຫ້ບໍລິການ***<br>0 = ບໍ່ແມ່ນ ***ຢືນຢັນຄຳຕອບໃນ S1 ຫຼື S2 ບໍ່ເທົ່າກັບ "1" ແລະ ປະເພດຮ້ານຄ້າທີ່ບັນທຶກໃນ C7 ແມ່ນ 17 ຫຼື "18" ("ຫ້ອງວິເຄາະເທົ່ານັ້ນ"). ໄປພາກທີ 6: ຟອມຕິດຕາມການກວດສອບ*** | [___] |

ລະຫັດຮ້ານຈຳໜ່າຍ: [___|___]-[___|___]-[___|___|___]-[___|___|___]

**ພາກທີ 5: ພາກຜູ້ໃຫ້ບໍລິການ**

*ພາກນີ້ແມ່ນສຳລັບພະນັກງານທີ່ອາວຸໂສທີ່ສຸດທີ່ຮັບຜິດຊອບການໃຫ້ບໍລິການການປິ່ນປົວ, ການອອກໃບສັ່ງຢາ ຫຼື ໃຫ້ຢາສຳລັບລູກຄ້າ ທີ່ຄົນເຈັບ*

| | |
|---|---|
| P1. ໜ້າທີ່ຮັບຜິດຊອບຂອງທ່ານໃນຮ້ານຈຳໜ່າຍ/ສະຖານທີ່ບໍລິການແຫ່ງນີ້ລວມມີ: ການອອກໃບສັ່ງຢາ, ການປິ່ນປົວ, ຫຼື ໃຫ້ຢາແກ່ລູກຄ້າບໍ່?<br>1 = ແມ່ນ<br>ບໍ່ແມ່ນ **ຂໍລົມກັບຜູ້ທີ່ອາວຸໂສທີ່ສຸດໃນຮ້ານຈຳໜ່າຍທີ່ມີ 1 ຫຼື ຫຼາຍໜ້າທີ່ຮັບຜິດຊອບ** | [___] |
| P2. ທ່ານເຮັດວຽກທີ່ຮ້ານຈຳໜ່າຍ/ສະຖານບໍລິການນີ້ໄດ້ຈັກປີແລ້ວ? ຖ້າວ່າຕ່ຳກວ່າ 1 ປີ, ໃຫ້ຂຽນ 01 | [___\|___] |
| P3. ປະຈຸບັນທ່ານອາຍຸຈັກປີແລ້ວ? ຂຽນອາຍຸເປັນປີ<br>97 = ປະຕິເສດ<br>98 = ບໍ່ຮູ້ | [___\|___] |
| P4. **ບໍ່ຕ້ອງອ່ານ:** ຜູ້ຖືກສຳພາດແມ່ນເພດຍິງ ຫຼື ເພດຊາຍ?<br>1 = ເພດຊາຍ<br>2 = ເພດຍິງ | [___] |
| P5. ທ່ານຈົບການສຶກສາສູງສຸດລະດັບໃດ?<br>1. = ບໍ່ໄດ້ເຂົ້າໂຮງຮຽນ<br>2. = ຊັ້ນປະຖົມແຕ່ບໍ່ຈົບ<br>3. = ຈົບຊັ້ນປະຖົມ<br>4. = ຊັ້ນມັດທະຍົມແຕ່ບໍ່ຈົບ<br>5. = ຈົບຊັ້ນມັດທະຍົມ<br>6. = ຍັງບໍ່ຈົບມະຫາວິທະຍາໄລ/ວິທະຍາໄລ/ວິຊາຊີບ<br>7. = ຈົບມະຫາວິທະຍາໄລ/ວິທະຍາໄລ/ວິຊາຊີບ | [___] |
| P6. ມີໃຜໃນຮ້ານຈຳໜ່າຍແຫ່ງນີ້ບໍ່ (ລວມທັງທ່ານເອງ) ທີ່ໄດ້ຮັບການອົບຮົມໃນ 12 ເດືອນຜ່ານມາ ທີ່ລວມມີເນື້ອໃນກ່ຽວກັບການບົ່ງມະຕິພະຍາດໄຂ້ມາເລເລຍ , ລວມທັງການກວດບົ່ງມະຕິແບບວາອງໄວສຳລັບພະຍາດໄຂ້ມາເລເລຍ ຫຼື ການໃຊ້ກ້ອງຈຸລະທັດ? **ລວມທັງການອົບຮົມໃນສະຖາບັນການຮຽນກ່ອນຈະເລີ່ມເຮັດວຽກຈິງ ແລະ ກອງປະຊຸມສຳມະນາວິຊາການຕ່າງໆ**<br>1 = ແມ່ນ<br>0 = ບໍ່ແມ່ນ **ໄປຂໍ້ P7**<br>8 = ບໍ່ຮູ້ **ໄປຂໍ້ P7** | [___] |
| P6a. ອົງການຈັດຕັ້ງໃດທີ່ໄດ້ສະໜອງການອົບຮົມ ທີ່ລວມມີເນື້ອໃນກ່ຽວກັບການບົ່ງມະຕິະຍາດໄຂ້ມາເລເລຍ ?<br>***ບໍ່ຕ້ອງອ່ານບັນຊີລາຍຊື່ ແລະ ຂີດວົງອ້ອມເອົາທັງໝົດທີ່ຜູ້ຕອບກ່າວເຖິງ***<br>***ຖາມເຈາະຈີ້ມຖ້າຄຳຕອບແມ່ນ "ອົງກອນອື່ນໆ" ຈົນກວ່າຜູ້ຕອບ ຕອບຄົບຖ້ວນ*** | |
| I. ລັດຖະບານ / ກະຊວງສາທາລະນະສຸກ / ສູນໄຂ້ຍຸງ, ແມ່ກາຝາກ ແລະ ແມງໄມ້ (ໂຄງການມີສ່ວນຮ່ວມຂອງລັດ ແລະ ເອກະຊົນ) | A |
| II. ອົງການອານາໄມໂລກ (WHO) | B |
| III. ອົງການສຸຂະພາບ ແລະ ຫຼຸດຜ່ອນຄວາມຍາກຈົນ (HPA) | C |
| IV. **ອື່ນໆ, ລະບຸ** [________________________] | X |

| | |
|---|---|
| V. **ບໍ່ຮູ້** | Z |
| P7. ມີໃຜໃນຮ້ານຈຳໜ່າຍແຫ່ງນີ້ບໍ່ (ລວມທັງທ່ານເອງ) ທີ່ໄດ້ຮັບການອົບຮົມໃນ 12 ເດືອນຜ່ານມາ ທີ່ລວມມີເນື້ອໃນກ່ຽວກັບຄູ່ມືການປິ່ນປົວພະຍາດໄຂ້ມາເລເລຍແຫ່ງຊາດ? **ລວມທັງການອົບຮົມໃນສະຖາບັນການຮຽນກ່ອນຈະເລີ່ມເຮັດວຽກຈິງ ແລະ ກອງປະຊຸມສຳມະນາວິຊາການຕ່າງໆ**<br>1 = ແມ່ນ<br>0 = ບໍ່ແມ່ນ **ໄປຂໍ້ P8**<br>8 = ບໍ່ຮູ້ **ໄປຂໍ້ P8** | [___] |
| P7a. ອົງການຈັດຕັ້ງໃດ ທີ່ໄດ້ສະໜອງການອົບຮົມກ່ຽວກັບ ຄູ່ມືການປິ່ນປົວພະຍາດໄຂ້ມາເລເລຍແຫ່ງຊາດ?<br>***ບໍ່ຕ້ອງອ່ານບັນຊີລາຍຊື່ ແລະ ຂີດວົງອ້ອມເອົາທັງໝົດທີ່ຜູ້ຕອບກ່າວເຖິງ***<br>***ຖາມເຈາະຈີ້ມຖ້າຄຳຕອບແມ່ນ "ອົງກອນອື່ນໆ" ຈົນກວ່າຜູ້ຕອບ ຕອບຄົບຖ້ວນ*** | |
| I. ພາກລັດ / ກະຊວງສາທາລະນະສຸກ/ສູນໄຂ້ມາເລເລຍ ແມ່ກາຝາກ ແລະ ແມງໄມ້ | A |
| II. ອົງການສຸຂະພາບ ແລະ ຫຼຸດຜ່ອນຄວາມຍາກຈົນ (HPA) | B |
| III. ອື່ນໆ, ລະບຸ [________________________________] | X |
| IV. ບໍ່ຮູ້ | Z |
| P8. ທ່ານມີຄຸນນະວຸດດ້ານສາທາລະນະສຸກດັ່ງຕໍ່ໄປນີ້ບໍ່?<br>***ບັນທຶກ 1 ສຳລັບ "ແມ່ນ"; 0 ສຳລັບ "ບໍ່ແມ່ນ"*** | |
| I. ເພສັດຊະກອນ (ແພດການຢາ) | [___] |
| II. ທ່ານໝໍ | [___] |
| III. ແພດຊ່ວຍ | [___] |
| IV. ພະຍາບານ | [___] |
| V. ຜະດຸງຄັນ | [___] |
| VI. ວິຊາການຢູ່ຫ້ອງວິເຄາະ/ ຜູ້ຊ່ວຍຢູ່ຫ້ອງວິເຄາະ | [___] |
| VII. ຜູ້ຊ່ວຍເພສັດຊະກອນ (ຜູ້ຊ່ວຍແພດການຢາ) | [___] |
| VIII. ຜູ້ຊ່ວຍພະຍາບານ | [___] |
| IX. ພະນັກງານແພດຂັ້ນບ້ານ / ອາສາສະໝັກແພດຂັ້ນບ້ານ | [___] |

| | |
|---|---|
| P9. ບໍ່ລວມຕົວທ່ານເອງ, ມີຄົນທີ່ເຮັດວຽກທີ່ຮ້ານຈຳໜ່າຍ ຫຼື ສະຖານທີ່ບໍລິການແຫ່ງນີ້ໄດ້ມີຄຸນນະວຸດດ້ານສາທາລະນະສຸກດັ່ງຕໍ່ໄປນີ້ບໍ່?<br>**ອ່ານບັນຊີລາຍຊື່ ແລະ *ບັນທຶກ 1 ສຳລັບ "ແມ່ນ"; 0 ສຳລັບ "ບໍ່ແມ່ນ"; 8 ສຳລັບ "ບໍ່ຮູ້"*** | |
| I. ເພສັດຊະກອນ (ແພດການຢາ) | [___] |
| II. ທ່ານໝໍ | [___] |
| III. ແພດຊ່ວຍ | [___] |
| IV. ພະຍາບານ | [___] |
| V. ຜະດຸງຄັນ | [___] |
| VI. ວິຊາການຢູ່ຫ້ອງວິເຄາະ/ ຜູ້ຊ່ວຍຢູ່ຫ້ອງວິເຄາະ | [___] |
| VII. ຜູ້ຊ່ວຍເພສັດຊະກອນ (ຜູ້ຊ່ວຍແພດການຢາ) | [___] |
| VIII. ຜູ້ຊ່ວຍພະຍາບານ | [___] |
| IX. ພະນັກງານແພດຂັ້ນບ້ານ / ອາສາສະໝັກແພດຂັ້ນບ້ານ | [___] |

| | |
|---|---|
| M1. ***ບໍ່ຕ້ອງອ່ານ.*** ຮ້ານຈຳໜ່າຍແຫ່ງນີ້ເປັນສະຖານທີ່ບໍລິການສາທາລະນະສຸກຂອງພາກລັດບໍ່. ໃນນັ້ນລວມມີໂຮງໝໍແຂວງ, ໂຮງໝໍເມືອງ, ສຸກສາລາ ຫຼື ອາສາສະໝັກຂັ້ນບ້ານ ທີ່ໄດ້ຮັບການຖືກຝຶກອົບຮົມຈາກສູນໄຂ້ຍຸງ. ກວດເບິ່ງຄືນກັບຄຳຖາມ *C7.*<br>1 = ແມ່ນ ***ໄປຂໍ້ P10***<br>0 = ບໍ່ແມ່ນ | [___] |
| M2. ຮ້ານຈຳໜ່າຍແຫ່ງນີ້ໄດ້ຮັບຢາປິ່ນປົວໄຂ້ມາເລເລຍ ໂດຍບໍ່ເສຍຄ່າ ຫຼື ຊື້ຢາປິ່ນປົວພະຍາດໄຂ້ມາເລເລຍ ທີ່ຫຼຸດລາຄາຈາກລັດຖະບານ ຫຼື ອົງການຈັດຕັ້ງທີ່ບໍ່ຂຶ້ນກັບລັດຖະບານບໍ່?<br>1 = ແມ່ນ<br>0 = ບໍ່ແມ່ນ ***ໄປຂໍ້ M4***<br>8 = ບໍ່ຮູ້ ***ໄປຂໍ້ M4*** | [___] |
| M3. ຮ້ານຈຳໜ່າຍແຫ່ງນີ້ໄດ້ຮັບຢາປິ່ນປົວພະຍາດໄຂ້ມາເລເລຍ ທີ່ບໍ່ເສຍຄ່າ ຫຼື ຫຼຸດລາຄາຈາກພາກສ່ວນໃດ?<br>***ບໍ່ຕ້ອງອ່ານບັນຊີລາຍຊື່, ຂີດວົງອ້ອມເອົາທັງໝົດທີ່ຜູ້ຕອບກ່າວເຖິງ*** | |
| I.ພາກລັດ/ສູນສະໜອງອຸປະກອນການແພດ (MPSC) | A |
| II. ພາກລັດສະເພາະເຈາະຈົງ/ສະຖານທີ່ບໍລິການສາທາລະນະສຸກຂອງພາກລັດ | B |
| III. ອົງການສຸຂະພາບ ແລະ ຫຼຸດຜ່ອນຄວາມຍາກຈົນ (HPA) | C |
| IV. ອື່ນໆ, ລະບຸ [______________________________] | X |
| M4. ຮ້ານຈຳໜ່າຍແຫ່ງນີ້ໄດ້ຮັບເຈ້ຍຈຸ່ມບົ່ງມະຕິແບບວ່ອງໄວ ໂດຍບໍ່ເສຍຄ່າ ຫຼື ຊື້ໃນແບບຫຼຸດລາຄາຈາກລັດຖະບານ ຫຼື ອົງການຈັດຕັ້ງທີ່ບໍ່ຂຶ້ນກັບລັດຖະບານບໍ່?<br>1 = ແມ່ນ<br>0 = ບໍ່ແມ່ນ ***ໄປຂໍ້ M6***<br>8 = ບໍ່ຮູ້ ***ໄປຂໍ້ M6*** | [___] |
| M5. ຮ້ານຈຳໜ່າຍແຫ່ງນີ້ໄດ້ຮັບເຈ້ຍຈຸ່ມບົ່ງມະຕິແບບວ່ອງໄວ ທີ່ບໍ່ເສຍຄ່າ ຫຼື ຫຼຸດລາຄາຈາກໃສ?<br>***ບໍ່ຕ້ອງອ່ານບັນຊີລາຍຊື່ ແລະ ຂີດວົງອ້ອມເອົາທັງໝົດທີ່ຜູ້ຕອບກ່າວເຖິງ*** | |
| I. ພາກລັດ/ສູນສະໜອງອຸປະກອນການແພດ (MPSC) | A |
| II. ພາກລັດສະເພາະເຈາະຈົງ/ສະຖານທີ່ບໍລິການສາທາລະນະສຸກຂອງພາກລັດ | B |
| III.ອື່ນໆ, ລະບຸ [______________________________] | X |
| M6. ໃນປີຜ່ານມາ, ຮ້ານຈຳໜ່າຍແຫ່ງນີ້ໄດ້ຮັບການລົງຢ້ຽມຢາມຈາກພາກລັດ ຫຼື ອົງການຈັດຕັ້ງທີ່ບໍ່ຂຶ້ນກັບລັດຖະບານບໍ່ໃນວຽກງານການສະໜັບສະໜູນ, ຮັບການຊີ້ນຳ ຫຼື ຮັບການການຕິດຕາມຊຸກຍູ້ບັນດາຮ້ານຈຳໜ່າຍໂດຍສະເພາະແມ່ນການຈັດການຄຸ້ມຄອງພະຍາດໄຂ້ມາເລເລຍ?<br>1 = ແມ່ນ<br>0 = ບໍ່ແມ່ນ ***ໄປຂໍ້ M9***<br>8 = ບໍ່ຮູ້ ***ໄປຂໍ້ M9*** | [___] |

ລະຫັດຮ້ານຈຳໜ່າຍ: [___|___]-[___|___]-[___|___|___]-[___|___|___]

| | |
|---|---|
| M7. ຮ້ານຈຳໜ່າຍແຫ່ງນີ້ໄດ້ຮັບການສະໜັບສະໜູນ, ການລົງຢ້ຽມຢາມຊີ້ນຳ ຫຼື ການຕິດຕາມຊຸກຍູ້ຈາກອົງການຈັດຕັ້ງໃດ?<br>***ບໍ່ຕ້ອງອ່ານບັນຊີລາຍຊື່ ແລະ ຂີດວົງອ້ອມເອົາທັງໝົດທີ່ຜູ້ຕອບກ່າວເຖິງ*** | |
| I. ກະຊວງສາທາລະນະສຸກ/ສູນໄຂ້ຍຸງ ແມງໄມ້ແລະແມ່ກາຝາກ | A |
| II. ກົມອາຫານ ແລະ ຢາ | B |
| III. ຫ້ອງການພະແນກສາທາແຂວງ | C |
| IV. ຫ້ອງການພະແນກສາທາເມືອງ | D |
| V. ອົງການສຸຂະພາບ ແລະ ຫຼຸດຜ່ອນຄວາມຍາກຈົນ (HPA) | E |
| ອື່ນໆ, ລະບຸ[______________________________] | X |
| M8. ຮ້ານຈຳໜ່າຍແຫ່ງນີ້ໄດ້ຮັບການສະໜັບສະໜຸນ, ລົງຢ້ຽມຢາມຊີ້ນຳ ຫຼື ຕິດຕາມຊຸກຍູ້ເລື້ອຍປານໃດ?<br>1 = ໜຶ່ງຄັ້ງ ຫຼື ຫຼາຍກວ່າ ຕໍ່ເດືອນ<br>2 = ໜຶ່ງຄັ້ງຕໍ່ 3 ເດືອນ<br>3 = ໜຶ່ງຄັ້ງຕໍ່ 6 ເດືອນ<br>4 = ໜຶ່ງຄັ້ງຕໍ່ປີ<br>5 = **ອື່ນໆ, ລະບຸ** [______________________________] | [___] |
| M9. ຮ້ານຈຳໜ່າຍແຫ່ງນີ້ໄດ້ບັນທຶກຂໍ້ມູນກ່ຽວກັບຈຳນວນຄົນເຈັບທີ່ໄດ້ຮັບການກວດຫາເຊື້ອໄຂ້ມາເລເລຍ ຫຼື ການປິ່ນປົວພະຍາດໄຂ້ມາເລເລຍ ບໍ່?<br>1 = ແມ່ນ<br>0 = ບໍ່ແມ່ນ ***ໄປຂໍ້ P10***<br>8 = ບໍ່ຮູ້ ***ໄປຂໍ້ P10*** | [___] |
| M10. ຂ້າພະເຈົ້າຂໍເບິ່ງລະບົບ ຫຼື ປື້ມບັນທຶກທີ່ໃຊ້ບັນທຶກຂໍ້ມູນຄົນເຈັບດັ່ງກ່າວໄດ້ບໍ່?<br>***ໝາຍ 1 ຖ້າວ່າສັງເກດເຫັນປື້ມບັນທຶກ ຫຼື ໝາຍ 0 ຖ້າວ່າບໍ່ສັງເກດເຫັນປື້ມບັນທຶກ***<br>1 = ສັງເກດເຫັນລະບົບ ຫຼື ປື້ມບັນທຶກ<br>0 = ບໍ່ສັງເກດເຫັນລະບົບ ຫຼື ປື້ມບັນທຶກ | [___] |
| M11. ລະບົບດັ່ງກ່າວໄດ້ບັນທຶກຂໍ້ມູນກ່ຽວກັບຄົນເຈັບແຕ່ລະຄົນບໍ່ (1 ແຖວໃນການລົງທະບຽນ = ຄົນເຈັບ1ຄົນ)?<br>1 = ແມ່ນ<br>0 = ບໍ່ແມ່ນ<br>8 = ບໍ່ຮູ້ | [___] |
| M12. ລະບົບດັ່ງກ່າວໄດ້ຮັກສາການຈົດບັນຊີ ຫຼື ການນັບຈຳນວນຂອງຄົນເຈັບທີ່ໄດ້ຮັບການກວດກາເຊື້ອໄຂ້ມາເລເລຍ ຫຼື ການປິ່ນປົວພະຍາດໄຂ້ມາເລເລຍ ເປັນເວລາທີ່ແນ່ນອນ ເຊັ່ນຕໍ່ມື້, ຕໍ່ອາທິດ, ຫຼື ຕໍ່ເດືອນ ບໍ່?<br>1 = ແມ່ນ 8 = ບໍ່ຮູ້<br>0 = ບໍ່ແມ່ນ | [___] |
| M13. ຂໍ້ມູນຈຳນວນຂອງຄົນເຈັບທີ່ໄດ້ຮັບການປິ່ນປົວ ຫຼື ການກວດຫາເຊື້ອໄຂ້ມາເລເລຍ ແມ່ນໄດ້ຖືກລາຍງານໃຫ້ແກ່ອົງການຈັດຕັ້ງໃດໜຶ່ງຂອງລັດ ຫຼື ອົງການຈັດຕັ້ງທີ່ບໍ່ຂຶ້ນກັບລັດຖະບານບໍ່?<br>1 = ແມ່ນ<br>0 = ບໍ່ແມ່ນ ***ໄປຂໍ້ P10***<br>8 = ບໍ່ຮູ້ ***ໄປຂໍ້ P10*** | [___] |

| | |
|---|---|
| M14. ຂໍ້ມູນຈຳນວນຂອງຄົນເຈັບທີ່ໄດ້ຮັບ ການກວດຫາເຊື້ອໄຂ້ມາເລເລຍ ຫຼື ໄດ້ຮັບການປິ່ນປົວ ແມ່ນໄດ້ຖືກລາຍງານໃຫ້ແກ່ອົງການຈັດຕັ້ງ ຫຼືພາກສ່ວນໃດ?<br>***ບໍ່ຕ້ອງອ່ານບັນຊີລາຍຊື່ ແລະ ຂີດວົງອ້ອມເອົາທັງໝົດທີ່ຜູ້ຕອບກ່າວເຖິງ*** | |
| I. ສູນໄຂ້ຍຸງ, ແມງໄມ້ ແລະ ແມ່ກາຝາກ | A |
| II. ແຂວງ (ລວມທັງ ໂຮງໝໍແຂວງ) | B |
| III. ເມືອງ (ລວມທັງໂຮງໝໍເມືອງ) | C |
| IV.ອື່ນໆ, ລະບຸ [________________________________] | X |

| | |
|---|---|
| M15. ໄດ້ມີການລາຍງານຈຳນວນຂອງຄົນເຈັບທີ່ໄດ້ຮັບການປິ່ນປົວ ຫຼື ການກວດພະຍາດໄຂ້ມາເລເລຍ ເລື້ອຍປານໃດ?<br>1 = ໜຶ່ງຄັ້ງຕໍ່ເດືອນ<br>2 = ໜຶ່ງຄັ້ງຕໍ່ 3 ເດືອນ<br>3 = ອື່ນໆ, **ລະບຸ**[________________________________] | [___] |
| M16. ທ່ານມີວິທີການແນວໃດໃນການແບ່ງປັນຂໍ້ມູນຄົນເຈັບກັບອົງການຈັດຕັ້ງເຫຼົ່ານີ້?<br>***ອ່ານບັນຊີລາຍຊື່ ແລະ ບັນທຶກ 1 ສຳລັບ ແມ່ນ, 0 ສຳລັບ ບໍ່ແມ່ນ, 8 ສຳລັບ ບໍ່ຮູ້*** | |
| I. ໂດຍເຈ້ຍແບບຟອມ/ບັນທຶກ | [___] |
| II. ໂດຍທາງໂທລະສັບ-ຂໍ້ຄວາມຜ່ານມືຖື | [___] |
| III. ໂດຍທາງໂທລະສັບ ແບບຟອມອີເລັກໂທນິກ | [___] |
| IV. ໂດຍທາງໂທລະເລກ (ແຟັກ) | [___] |
| V. ໂດຍທາງໂທລະສັບ-ລາຍງານປາກເປົ່າ | [___] |
| VI. ດ້ວຍຕົວເອງ - ລາຍງານດ້ວຍປາກເປົ່າ | [___] |
| VII. ໂດຍຄອມພິວເຕີ້-ແບບຟອມອີເລັກໂທຣນິກ | [___] |

ລະຫັດຮ້ານຈໍາໜ່າຍ: [___|___]-[___|___]-[___|___|___]-[___|___|___]

***ນັກສໍາພາດ:*** ສໍາລັບຄໍາຖາມຕໍ່ໄປນີ້ບັນທຶກຊື່ຍີ່ຫໍ້, ຊື່ຕົວຢາຂອງຢາປິ່ນປົວໄຂ້ມາເລເລຍ , ແລະ ຮູບແບບຂອງຢາ, ໃນບ່ອນວ່າງທີ່ຈັດກຽມໄວ້. ຂໍໃຫ້ຜູ້ບໍລິການເອົາຢາໃຫ້ທ່ານເບິ່ງ ຖ້າວ່າມັນມີຢູ່ໃນຮ້ານເພື່ອຢືນຢັນຄວາມຖືກຕ້ອງຂອງຊື່ ແລະ ຮູບແບບຂອງຢາ.

P10. ໃນຄວາມຄິດຂອງທ່ານ, ສໍາລັບການປິ່ນປົວໄຂ້ມາເລເລຍທີ່ບໍ່ມີອາການສົນໃນຜູ້ໃຫຍ່, ແມ່ນຫຍັງທີ່ເປັນການປິ່ນປົວທີ່ໄດ້ຜົນທີ່ສຸດ? **ຂໍໃຫ້ຜູ້ບໍລິການເອົາຢາໃຫ້ທ່ານເບິ່ງຖ້າວ່າມັນມີຢູ່ໃນຮ້ານ.**

| **ຊື່ຍີ່ຫໍ້ ຫຼື ຊື່ຕົວຢາ** | **ຮູບແບບຢາ/ສູດສະແດງ**<br>01 = ຢາເມັດ 04 = ຢານໍ້າເຊື່ອມ 07 = ຢາຢອດ<br>02 = ຢາຍັດ 05 = ຢານໍ້າແຂວນຕະກອນ 95 = ບໍ່ໄດ້ລະບຸ<br>03 = ຢາຝຸ່ນເປັນຊອງ 06 = ຢາສັກ (ນໍ້າຫຼືຝຸ່ນ) 98 = ບໍ່ຮູ້ |
|---|---|
| [______________________________]<br>**ບໍ່ຮູ້ = *98*** | [___|___] |

| **ຊື່ຍີ່ຫໍ້ ຫຼື ຊື່ຕົວຢາ** | **ຮູບແບບຢາ/ສູດສະແດງ**<br>01 = ຢາເມັດ 04 = ຢານໍ້າເຊື່ອມ 07 = ຢາຢອດ<br>02 = ຢາຍັດ 05 = ຢານໍ້າແຂວນຕະກອນ 95 = ບໍ່ໄດ້ລະບຸ<br>03 = ຢາຝຸ່ນເປັນຊອງ 06 = ຢາສັກ (ນໍ້າຫຼືຝຸ່ນ) 98 = ບໍ່ຮູ້ |
|---|---|
| [______________________________]<br>**ບໍ່ຮູ້ = *98*** | [___|___] |

| **ຊື່ຍີ່ຫໍ້ ຫຼື ຊື່ຕົວຢາ** | **ຮູບແບບຢາ/ສູດສະແດງ**<br>01 = ຢາເມັດ 04 = ຢານໍ້າເຊື່ອມ 07 = ຢາຢອດ<br>02 = ຢາຍັດ 05 = ຢານໍ້າແຂວນຕະກອນ 95 = ບໍ່ໄດ້ລະບຸ<br>03 = ຢາຝຸ່ນເປັນຊອງ 06 = ຢາສັກ (ນໍ້າຫຼືຝຸ່ນ) 98 = ບໍ່ຮູ້ |
|---|---|
| [______________________________]<br>**ບໍ່ຮູ້ = *98*** | [___|___] |

ລະຫັດຮ້ານຈຳໜ່າຍ: [___|___]-[___|___]-[___|___|___]-[___|___|___]

P13. ການປິ່ນປົວໄຂ້ມາເລເລຍ ທີ່ບໍ່ມີອາການສົນໃນຜູ້ໃຫຍ່ແບບໃດ ທີ່ສ່ວນໃຫ່ຍທ່ານມັກຈະແນະນຳໃຫ້ແກ່ລູກຄ້າ?

*ຖາມຜູ້ໃຫ້ບໍລິການເອົາຢາໃຫ້ເບິ່ງ ຖ້າວ່າມີຢູ່ໃນຮ້ານ*

| ຊື່ຍີ່ຫໍ້ ຫຼື ຊື່ຕົວຢາ | ຮູບແບບຢາ/ສູດສະແດງ<br>01 = ຢາເມັດ 04 = ຢານ້ຳເຊື່ອມ 07 = ຢາຍອດ<br>02 = ຢາຍັດ 05 = ຢານ້ຳແຂວນຕະກອນ 95 = ບໍ່ໄດ້ລະບຸ<br>03 = ຢາຝຸ່ນເປັນຊອງ 06 = ຢາສັກ (ນ້ຳຫຼືຝຸ່ນ) 98 = ບໍ່ຮູ້ |
|---|---|
| [________________]<br>ບໍ່ຮູ້ = *98* | [___\|___] |

| ຊື່ຍີ່ຫໍ້ ຫຼື ຊື່ຕົວຢາ | ຮູບແບບຢາ/ສູດສະແດງ<br>01 = ຢາເມັດ 04 = ຢານ້ຳເຊື່ອມ 07 = ຢາຍອດ<br>02 = ຢາຍັດ 05 = ຢານ້ຳແຂວນຕະກອນ 95 = ບໍ່ໄດ້ລະບຸ<br>03 = ຢາຝຸ່ນເປັນຊອງ 06 = ຢາສັກ (ນ້ຳຫຼືຝຸ່ນ) 98 = ບໍ່ຮູ້ |
|---|---|
| [________________]<br>ບໍ່ຮູ້ = *98* | [___\|___] |

| ຊື່ຍີ່ຫໍ້ ຫຼື ຊື່ຕົວຢາ | ຮູບແບບຢາ/ສູດສະແດງ<br>01 = ຢາເມັດ 04 = ຢານ້ຳເຊື່ອມ 07 = ຢາຍອດ<br>02 = ຢາຍັດ 05 = ຢານ້ຳແຂວນຕະກອນ 95 = ບໍ່ໄດ້ລະບຸ<br>03 = ຢາຝຸ່ນເປັນຊອງ 06 = ຢາສັກ (ນ້ຳຫຼືຝຸ່ນ) 98 = ບໍ່ຮູ້ |
|---|---|
| [________________]<br>ບໍ່ຮູ້ = *98* | [___\|___] |

| ຊື່ຍີ່ຫໍ້ ຫຼື ຊື່ຕົວຢາ | ຮູບແບບຢາ/ສູດສະແດງ<br>01 = ຢາເມັດ 04 = ຢານ້ຳເຊື່ອມ 07 = ຢາຍອດ<br>02 = ຢາຍັດ 05 = ຢານ້ຳແຂວນຕະກອນ 95 = ບໍ່ໄດ້ລະບຸ<br>03 = ຢາຝຸ່ນເປັນຊອງ 06 = ຢາສັກ (ນ້ຳຫຼືຝຸ່ນ) 98 = ບໍ່ຮູ້ |
|---|---|
| [________________]<br>ບໍ່ຮູ້ = *98* | [___\|___] |

ລະຫັດຮ້ານຈຳໜ່າຍ: [___|___]-[___|___]-[___|___|___]-[___|___|___]

| P16. ກະລຸນາບອກຊື່ການປິ່ນປົວຂັ້ນທຳອິດ ທີ່ແນະນຳໂດຍລັດຖະບານ ໃນການປິ່ນປົວພະຍາດໄຂ້ມາເລເລຍ ພາດຊະໂມດຽມ ຟາຊິພາລອມ (Pf) ທີ່ຍັງບໍ່ມີອາການສົນ ສຳລັບຜູ້ໃຫຍ່ (60kg).<br>***ບໍ່ຕ້ອງອ່ານບັນຊີລາຍຊື່ ແລະ ຂີດວົງອ້ອມເອົາທັງໝົດທີ່ຜູ້ຕອບກ່າວເຖິງ*** | |
|---|---|
| ອາເຕເມເທີ ລູມີແຟນທີນ (ຄອດເທີມ, ຄອມບຽດ, ອາເຕຟານ)<br>Artemether Lumefantrine (Coartem, Combiart, Artefan) | A |
| ພຣີມາກີນ (Primaquine) | B |
| ອື່ນໆ ລະບຸ [________________________] | X |
| ບໍ່ຮູ້ | Z |

**ຄຳແນະນຳສຳລັບການຂ້າມ**

***ຖ້າວ່າ ອາເຕເມເທີ ລູມີແຟນທີນ Artemether Lumefantrine ຖືກຂີດອ້ອມເອົາ >>> ໄປຕໍ່ຂໍ້ P17a***

***ຖ້າວ່າ ອາເຕເມເທີ ລູມີແຟນທີນ Artemether Lumefantrine ບໍ່ຖືກຂີດອ້ອມເອົາ >>> ໄປຕໍ່ຂໍ້ P20a***

| P17a. ກະລຸນາອະທິບາຍລະບົບການປິ່ນປົວທີ່ແນະນຳໂດຍລັດຖະບານສຳລັບ *ອາເຕເມເທີ ລູມີແຟນທີນ* (Artemether Lumefantrine) ເພື່ອການປິ່ນປົວພະຍາດໄຂ້ມາເລເລຍ ພາດຊະໂມດຽມ ຟາຊິພາລອມ (*Pf*) ທີ່ຍັງບໍ່ມີອາການສົນ ໃນຜູ້ໃຫຍ່ (60kg). | |
|---|---|
| ***ຖ້າວ່າຜູ້ໃຫ້ບໍລິການມີຢາຢູ່, ນຳໃຊ້ຫໍ່ຢາເພື່ອຕື່ມລາຍລະອຽດຂອງຢາ***<br>***ຖ້າບໍ່ມີຢາ, ຂໍໃຫ້ຜູ້ໃຫ້ບໍລິການລະບຸຢາດັ່ງກ່າວຈາກບັດສະແດງ***<br>***ຖ້າ ຍັງບໍ່ສາມາດລະບຸໄດ້, ຂໍໃຫ້ຜູ້ໃຫ້ບໍລິການຄິດຄືນກ່ຽວກັບລາຍລະອຽດຂອງຢາ*** | |
| *i. ຄວາມແຮງຂອງ ອາເຕເມເທີ (Artemether) ແມ່ນເທົ່າໃດ?* | [__\|__\|__].[__]mg |
| *ii. ຄວາມແຮງຂອງ ລູມີແຟນທີນ (Lumefantrine) ແມ່ນເທົ່າໃດ?* | [__\|__\|__].[__]mg<br>ບໍ່ຮູ້ *= 999.8* |
| ***ອ່ານ 3 ຄຳຖາມຕໍ່ໄປນີ້ໃຫ້ຜູ້ໃຫ້ບໍລິການຟັງ (ບໍ່ບັນທຶກຂໍ້ມູນນີ້ຈາກຫໍ່ຢາ).*** | |
| iv. ເຂົາເຈົ້າຄວນກິນ ອາເຕເມເທີ ລູມີແຟນທີນ (ຄອດເທີມ, ຄອມບຽດ, ອາເຕຟານ) - Artemether Lumefantrine (Coartem, Combiart, Artefan) ຈັກເມັດໃນຄັ້ງໜຶ່ງ? | [___\|___].[___\|___] |
| v. ຄວນກິນຢາ ອາເຕເມເທີ ລູມີແຟນທີນ (ຄອດເທີມ, ຄອມບຽດ, ອາເຕຟານ) - Artemether Lumefantrine (Coartem, Combiart, Artefan) ຈັກເທື່ອຕໍ່ມື້? | [___\|___] |
| vi. ຄວນກິນຢາ ອາເຕເມເທີ ລູມີແຟນທີນ (ຄອດເທີມ, ຄອມບຽດ, ອາເຕຟານ) - Artemether Lumefantrine (Coartem, Combiart, Artefan) ເປັນເວລາຈັກມື້? | [___\|___]<br>***ບໍ່ຮູ້*** *= 98* |

| P18. ກະລຸນາບອກຊື່ຢາທີ່ເປັນຕົວເລືອກຂັ້ນທຳອິດ ທີ່ແນະນຳໂດຍລັດຖະບານໃນການປິ່ນປົວພະຍາດໄຂ້ມາເລເລຍ ທີ່ບໍ່ມີອາການສົນ ພາດຊະໂມດຽມ ວີວັກ (*Plasmodium vivax*) ທີ່ບໍ່ມີອາການສົນ ໃນຜູ້ໃຫຍ່ (60kg).<br>***ບໍ່ຕ້ອງອ່ານບັນຊີລາຍຊື່ ແລະ ຂີດວົງອ້ອມເອົາທັງໝົດທີ່ຜູ້ຕອບກ່າວເຖິງ*** | |
|---|---|
| ອາເຕເມເທີ ລູມີແຟນທີນ (ຄອດເທີມ, ຄອມບຽດ, ອາເຕຟານ)<br>Artemether Lumefantrine (Coartem, Combiart, Artefan) | A |
| ພຣີມາກີນ (Primaquine) | B |
| **ອື່ນໆລະບຸ** [______________________________] | X |
| ບໍ່ຮູ້ | Z |

**ຄຳແນະນຳສຳລັບການຂ້າມ**

**ຖ້າວ່າ *ອາເຕເມເທີ ລູມີແຟນທີນ Artemether Lumefantrine* ຖືກຂີດອ້ອມເອົາ *>>> ໄປຕໍ່ຂໍ້ P19a***

**ຖ້າວ່າ *ອາເຕເມເທີ ລູມີແຟນທີນ Artemether Lumefantrine* ແລະ *Primaquine* ທັງສອງແມ່ນບໍ່ຖືກຂີດອ້ອມເອົາ *>>> ໄປຕໍ່ຂໍ້ P20a***

**ຖ້າວ່າມີແຕ່ ພີຣມາກີນ *Primaquine* ຖືກຂີດອ້ອມເອົາ *>>> ໄປຕໍ່ຂໍ້ P20b***

| P19a. ກະລຸນາອະທິບາຍລະບົບການປິ່ນປົວທີ່ແນະນຳໂດຍລັດຖະບານສຳລັບ *ອາເຕເມເທີ ລູມີແຟນທີນ* (Artemether Lumefantrine) ເພື່ອການປິ່ນປົວພະຍາດໄຂ້ມາເລເລຍ ພາດຊະໂມດຽມ ວີວັກ (*Pv*) ທີ່ຍັງບໍ່ມີອາການສົນ ໃນຜູ້ໃຫຍ່ (60kg).<br>***ຖ້າວ່າຜູ້ໃຫ້ບໍລິການມີຢາຢູ່, ນຳໃຊ້ທີ່ຢາເພື່ອຕື່ມລາຍລະອຽດຂອງຢາ.***<br>***ຖ້າບໍ່ມີຢາ, ຂໍໃຫ້ຜູ້ໃຫ້ບໍລິການລະບຸຢາດັ່ງກ່າວຈາກບັດສະແດງ***<br>***ຖ້າ ຍັງບໍ່ສາມາດລະບຸໄດ້, ຂໍໃຫ້ຜູ້ໃຫ້ບໍລິການຄິດຄືນກ່ຽວກັບລາຍລະອຽດຂອງຢາ*** | |
|---|---|
| *i. ຄວາມແຮງຂອງ ອາເຕເມເທີ (Artemether) ແມ່ນເທົ່າໃດ?* | [__\|__\|__].[__]mg |
| *ii. ຄວາມແຮງຂອງ ລູມີແຟນທີນ (Lumefantrine) ແມ່ນເທົ່າໃດ?* | [__\|__\|__].[__]mg<br>**ບໍ່ຮູ້** *= 999.8* |
| ***ອ່ານ 3 ຄຳຖາມຕໍ່ໄປນີ້ໃຫ້ຜູ້ໃຫ້ບໍລິການຟັງ (ບໍ່ບັນທຶກຂໍ້ມູນນີ້ຈາກທີ່ຢາ).*** | |
| iv. ເຂົາເຈົ້າຄວນກິນ ອາເຕເມເທີ ລູມີແຟນທີນ (ຄອດເທີມ, ຄອມບຽດ, ອາເຕຟານ) - Artemether Lumefantrine (Coartem, Combiart, Artefan) ຈັກເມັດໃນຄັ້ງໜຶ່ງ? | [___\|___].[___\|___] |
| v. ຄວນກິນຢາ ອາເຕເມເທີ ລູມີແຟນທີນ (ຄອດເທີມ, ຄອມບຽດ, ອາເຕຟານ) - Artemether Lumefantrine (Coartem, Combiart, Artefan) ຈັກເທື່ອຕໍ່ມື້? | [___\|___] |
| vi. ຄວນກິນຢາ ອາເຕເມເທີ ລູມີແຟນທີນ (ຄອດເທີມ, ຄອມບຽດ, ອາເຕຟານ) - Artemether Lumefantrine (Coartem, Combiart, Artefan) ເປັນເວລາຈັກມື້? | [___\|___]<br>**ບໍ່ຮູ້** *= 98* |

**ຄຳແນະນຳສຳລັບການຂ້າມ**

**ຖ້າວ່າ *ພີຣມາກີນ Primaquine* ຖືກຂີດອ້ອມເອົາ ໃນ *P18 >>> ໄປຕໍ່ຂໍ້ P19b***

**ຖ້າວ່າ *ພີຣມາກີນ Primaquine* ບໍ່ຖືກຂີດອ້ອມເອົາ ໃນ *P18 >>> ໄປຕໍ່ຂໍ້ P20a***

| | |
|---|---|
| P19b. ກະລຸນາອະທິບາຍລະບົບການປິ່ນປົວທີ່ແນະນຳໂດຍລັດຖະບານສຳລັບ *ພີຣມາກີນ (Primaquine)* ເພື່ອການປິ່ນປົວພະຍາດໄຂ້ມາເລເລຍ <u>ພາດຊະໂມດຽມ ວີວັກ</u> (*P*v) ທີ່ຍັງບໍ່ມີອາການສົນ ໃນຜູ້ໃຫຍ່ <u>(60kg)</u>.<br>***ຖ້າວ່າຜູ້ໃຫ້ບໍລິການມີຢາຢູ່, ນຳໃຊ້ຫໍ່ຢາເພື່ອຕື່ມລາຍລະອຽດຂອງຢາ.***<br>***ຖ້າບໍ່ມີຢາ, ຂໍໃຫ້ຜູ້ໃຫ້ບໍລິການລະບຸຢາດັ່ງກ່າວຈາກບັດສະແດງ***<br>***ຖ້າ ຍັງບໍ່ສາມາດລະບຸໄດ້, ຂໍໃຫ້ຜູ້ໃຫ້ບໍລິການຄິດຄືນກ່ຽວກັບລາຍລະອຽດຂອງຢາ***<br>*i. ຄວາມແຮງຂອງ ພີຣມາກີນ (Primaquine)* ***ແມ່ນເທົ່າໃດ****?*<br>***ອ່ານ 3 ຄຳຖາມຕໍ່ໄປນີ້ໃຫ້ຜູ້ໃຫ້ບໍລິການຟັງ (ບໍ່ບັນທຶກຂໍ້ມູນນີ້ຈາກຫໍ່ຢາ).***<br>iii. ຄວນກິນ ພີຣມາກີນ (Primaquine) ຈັກເມັດໃນຄັ້ງໜຶ່ງ?<br>iv. ຄວນກິນຢາ ພີຣມາກີນ (Primaquine) ຈັກເທື່ອຕໍ່ມື້?<br>v. ຄວນກິນ ພຣີມາກີນ (Primaquine) ເປັນເວລາຈັກມື້? | [__\|__\|__].[__]mg<br>ບໍ່ຮູ້ ***= 999.8***<br>[___\|___].[___\|___]<br>[___\|___]<br>[___\|___]<br>ບໍ່ຮູ້ ***= 98*** |
| **<u>ຄຳແນະນຳສຳລັບການຂ້າມ</u>**<br>***ໄປຕໍ່ທີ່ຂໍ້ P20b*** | |

| | |
|---|---|
| P20a. ທ່ານເຄີຍເຫັນ ຫຼື ໄດ້ຍິນກ່ຽວກັບຢາທີ່ຮ້ອງວ່າ ພຣີມາກີນ primaquine ມາກ່ອນບໍ່?<br>1 = ແມ່ນ ***ໄປທີ່ຂໍ້ P20b***<br>0 = ບໍ່ແມ່ນ ***ໄປທີ່ຂໍ້ P21***<br>8 = ບໍ່ຮູ້ ***ໄປທີ່ຂໍ້ P21*** | [___] |
| P20b. ຢາພຣີມາກີນ primaquine ແມ່ນຖືກນຳໃຊ້ເພື່ອຫຍັງ?<br>***<u>ບໍ່ຕ້ອງອ່ານບັນຊີລາຍຊື່</u> ແລະ ຂີດວົງອ້ອມເອົາທັງໝົດທີ່ຜູ້ຕອບກ່າວເຖິງ*** | |
| ໃຊ້ເປັນຢາປິ່ນປົວພະຍາດໄຂ້ມາເລເລຍ /ເພື່ອປິ່ນປົວໄຂ້ມາເລເລຍ | A |
| ໃຊ້ເປັນຢາຂ້າເຊື້ອ <u>ກາມີໂຕຊາຍ</u> / ເພື່ອປ້ອງກັນການສົ່ງຕໍ່ຂອງເຊື້ອໄຂ້ມາເລເລຍ | B |
| ໃຊ້ເປັນການປິ່ນປົວໄຂ້ມາເລເລຍ <u>ພາດຊະໂມດຽມ ວີວັກ</u> (Pv)<br>/ເພື່ອປ້ອງກັນການກັບມາເປັນຄືນຂອງ <u>ພາດຊະໂມດຽມ ວີວັກ</u> (Pv) | C |
| ບໍ່ຮູ້ | Z |
| P20c. ທ່ານເຄີຍໄດ້ຂຽນໃບສັ່ງຢາ ພຣີມາກີນ primaquine ໃຫ້ຄົນເຈັບ/ລູກຄ້າບໍ່?<br>1 = *ແມ່ນ*<br>0 = *ບໍ່ແມ່ນ* | [___] |
| P20d. ທ່ານເຄີຍໃຫ້ບໍລິການ ຫຼື ຂາຍຢາ ພຣີມາກີນ primaquine ໃຫ້ແກ່ຄົນເຈັບ/ລູກຄ້າບໍ່?<br>1 = *ແມ່ນ*<br>0 = *ບໍ່ແມ່ນ* | [___] |

| | |
|---|---|
| P21. ການກວດບົ່ງມະຕິພະຍາດໄຂ້ມາເລເລຍ ແບບຮູ້ຜົນວ່ອງໄວ, ຫຼື ເອີ້ນວ່າ ກວດສອບດ້ວຍເຈ້ຍຈຸ່ມບົ່ງມະຕິແບບວ່ອງໄວ, ມັນມີຂະໜາດນ້ອຍ ແລະ ມາເປັນຊອງຂອງໃຜຂອງມັນທີ່ສາມາດບົ່ງມະຕິແບບວ່ອງໄວວ່າຄົນຜູ້ນັ້ນມີເຊື້ອໄຂ້ມາເລເລຍ ຫຼືບໍ່, ເອົາບັດສະແດງຮູບພາບເຄື່ອງກວດໃຫ້ຜູ້ຕອບເບິ່ງ<br><br>ທ່ານເຄີຍເຫັນ ຫຼື ໄດ້ຍິນກ່ຽວກັບ ເຈ້ຍຈຸ່ມບົ່ງມະຕິແບບວ່ອງໄວກວດຫາເຊື້ອໄຂ້ມາເລເລຍບໍ່?<br>1 = ແມ່ນ **ໄປຂໍ້ P22**<br>0 = ບໍ່ແມ່ນ **ໄປພາກທີ 6**<br>8 = ບໍ່ຮູ້ **ໄປພາກທີ 6** | [___] |
| P22. ທ່ານເຄີຍໄດ້ກວດໄຂ້ມາເລເລຍໃຫ້ລູກຄ້າໂດຍໃຊ້ເຈ້ຍຈຸ່ມບົ່ງມະຕິແບບວ່ອງໄວບໍ່?<br>1 = ແມ່ນ<br>0 = ບໍ່ແມ່ນ<br>8 = ບໍ່ຮູ້ | [___] |
| P23. ທ່ານຈະແນະນຳໃຫ້ຄົນເຈັບ/ລູກຄ້າກິນຢາປິ່ນປົວໄຂ້ມາເລເລຍບໍ່ ຖ້າຜົນກວດເລືອດຊອກຫາເຊື້ອມາເລເລຍ ໂດຍໃຊ້ເຈ້ຍຈຸ່ມບົ່ງມະຕິແບບວ່ອງໄວບໍ່ພົບເຊື້ອ (ໃຫ້ຜົນລົບ)?<br>ອ່ານບັນຊີລາຍຊື່. **ບັນທຶກພຽງຄຳຕອບດຽວ**<br><br>1 = ແມ່ນ, ບາງເທື່ອ<br>2 = ແມ່ນ, ຕະຫຼອດ<br>3 = ບໍ່, ບໍ່ເຄີຍ ***ໄປພາກທີ 6***<br>8 = ບໍ່ຮູ້ ***ໄປພາກທີ 6*** | [___] |
| P24. ໃນກໍລະນີໃດທ່ານຈະແນະນຳໃຫ້ຄົນເຈັບ/ລູກຄ້າກິນຢາປິ່ນປົວໄຂ້ມາເລເລຍ ຖ້າຜົນກວດເລືອດຊອກຫາ ເຊື້ອມາເລເລຍໂດຍໃຊ້ ເຈ້ຍຈຸ່ມບົ່ງມະຕິແບບວ່ອງໄວບໍ່ພົບເຊື້ອ (ໃຫ້ຜົນລົບ)?<br>***ບໍ່ຕ້ອງອ່ານບັນຊີລາຍຊື່ ເຈາະຈີ້ມ "ມີຫຍັງອີກບໍ່" ຈົນກວ່າຜູ້ຖືກສຳພາດຈະຕອບວ່າບໍ່ມີ ຂີດວົງອ້ອມທຸກຄຳຕອບທີ່ຜູ້ຕອບກ່າວເຖິງ*** | |
| ເວລາທີ່ເຂົາເຈົ້າມີອາການ/ໝວດອາການຂອງໄຂ້ມາເລເລຍ | A |
| ເວລາທີ່ເຂົາເຈົ້າຂໍຢາປິ່ນປົວພະຍາດໄຂ້ມາເລເລຍ | B |
| ເຫັນວ່າເຂົາເຈົ້າເປັນເດັກນ້ອຍ | C |
| ເຫັນວ່າເຂົາເຈົ້າເປັນຜູ້ໃຫຍ່ | D |
| ເຫັນວ່າເຂົາເຈົ້າເປັນຜູ້ຍິງຖືພາ | E |
| ເມື່ອຂ້າພະເຈົ້າບໍ່ເຊື່ອຜົນກວດ | F |
| ເມື່ອຂ້າພະເຈົ້າຮູ້ຈັກຄົນເຈັບ/ລູກຄ້າ | G |
| ອື່ນໆ ລະບຸ [________________________] | X |

***ຕື່ມເຈ້ຍບັນທຶກຕິດຕາມການກວດສອບ ໃນໜ້າຕໍ່ໄປແລ້ວປະຕິບັດຕາມຄຳແນະນຳສຳລັບການຈົບການສຳພາດ***

| **ພາກທີ 6: ເຈ້ຍບັນທຶກການຕິດຕາມການກວດສອບ** | |
|---|---|
| T1. ໃນຮ້ານຈຳໜ່າຍແຫ່ງນີ້ມີຢາປິ່ນປົວໄຂ້ມາເລເລຍ ທີ່ເປັນຮູບແບບຢາເມັດ/ຢາຢັດ/ຢາຝຸ່ນເປັນຊອງ (TSG) ຢູ່ໃນຮ້ານບໍ່?<br>1 = ມີ<br>0 = ບໍ່ມີ ***ໄປຂໍ້ T4***<br>8 = ບໍ່ຮູ້ ***ໄປຂໍ້ T4*** | [___] |
| T2. ຈຳນວນເຈ້ຍກວດສອບທັງໝົດຂອງຢາເມັດ/ຢາຢັດ/ຢາຝຸ່ນເປັນຊອງ (TSG) ທີ່ໄດ້ຕື່ມຄົບໝົດ | [___\|___] |
| T3. ທ່ານໄດ້ຕື່ມຂໍ້ມູນໃນຟອມກວດສອບສຳລັບຢາເມັດ/ຢາຢັດ/ຢາຝຸ່ນເປັນຊອງ (TSG) ທີ່ມີຢູ່ທັງໝົດບໍ່?<br>1 = ແມ່ນ, ການກວດສອບໄດ້ສຳເລັດ<br>0 = ບໍ່, ການກວດສອບບໍ່ສຳເລັດ | [___] |
| T4. ໃນຮ້ານຈຳໜ່າຍນີ້ມີຢາປິ່ນປົວໄຂ້ມາເລເລຍ ທີ່ບໍ່ແມ່ນຢາເມັດ (ຢານ້ຳເຊື່ອມ, ຢານ້ຳແຂວນຕະກອນ, ຢາສັກ) (NT) ບໍ່?<br>1 = ມີ<br>0 = ບໍ່ມີ ***ໄປຂໍ້ T7***<br>8 = ບໍ່ຮູ້ ***ໄປຂໍ້ T7*** | [___] |
| T5. ຈຳນວນເຈ້ຍກວດສອບຂອງຢາປິ່ນປົວໄຂ້ມາເລເລຍທີ່ບໍ່ແມ່ນຢາເມັດ (NT) ໄດ້ຖືກຕື່ມຄົບໝົດ | [___\|___] |
| T6. ທ່ານໄດ້ຕື່ມຂໍ້ມູນໃນຟອມກວດສອບສຳລັບຢາທີ່ບໍ່ແມ່ນຢາເມັດ (NT) ຄົບໝົດບໍ່?<br>1 = ແມ່ນ, ການກວດສອບໄດ້ສຳເລັດ<br>0 = ບໍ່, ການກວດສອບບໍ່ສຳເລັດ | [___] |
| T7. ໃນຮ້ານຈຳໜ່າຍນີ້ມີເຈ້ຍຈຸ່ມບົ່ງມະຕິແບບວ່ອງໄວ (RDT) ຢູ່ໃນຮ້ານບໍ່?<br>1 = ມີ<br>0 = ບໍ່ມີ ***ໄປຂໍ້ T10***<br>8 = ບໍ່ຮູ້ ***ໄປຂໍ້ T10*** | [___] |
| T8. ຈຳນວນຟອມກວດສອບຂອງເຈ້ຍຈຸ່ມບົ່ງມະຕິແບບວ່ອງໄວ (RDT) ໄດ້ຖືກຕື່ມຂໍ້ມູນຄົບໝົດ | [___\|___] |
| T9. ຈຳນວນຟອມກວດສອບຂອງເຈ້ຍຈຸ່ມບົ່ງມະຕິແບບວ່ອງໄວ (RDT) ໄດ້ຖືກຕື່ມຂໍ້ມູນຄົບໝົດ<br>1 = ແມ່ນ, ການກວດສອບໄດ້ສຳເລັດ<br>0 = ບໍ່, ການກວດສອບບໍ່ສຳເລັດ | [___] |
| T10. ໃນຮ້ານຈຳໜ່າຍນີ້, ເຄີຍມີຜະລິດຕະພັນໃດທີ່ບໍ່ມີຢູ່ໃນຮ້ານ (ຂາດສາງ) ບໍ່, ແຕ່ທ່ານເຄີຍມີ ໃນ 3 ເດືອນຜ່ານທີ່ມາ?<br>1 = ມີ<br>0 = ບໍ່ມີ ***ໄປຂໍ້ T12***<br>8 = ບໍ່ຮູ້ ***ໄປຂໍ້ T12*** | [___] |
| T11. ລວມຈຳນວນຜະລິດຕະພັນ ທີ່ບໍ່ມີຢູ່ໃນຮ້ານ (ຂາດສາງ) ທີ່ບັນທຶກຢູ່ໃນຄຳຖາມຂໍ້ A17a | [___\|___] |
| T12. ຄວາມເຫັນຕ່າງໆ: ເຫດຜົນທີ່ບໍ່ສາມາດສຳເລັດເຈ້ຍກວດສອບ (ຖ້າຄຳຕອບ ໃນຂໍ້ T3, T6, ຫຼື T9 ຕອບ "ບໍ່") | |

***ດຳເນີນການຕື່ມຂໍ້ C9 ແລະ ບັນທຶກຜົນຂອງການສຳພາດ ແລະ ເວລາທີ່ສຳເລັດການສຳພາດ, ຈາກນັ້ນໄປ ພາກ X: ເສັດສິ້ນການສຳພາດ.***
