# Supplementary material for: The malaria testing and treatment landscape in the southern Lao People’s Democratic Republic (PDR)
Source: Malar J. 2017 Apr 25;16:169. doi: 10.1186/s12936-017-1769-0 (PMC5404290; doi:10.1186/s12936-017-1769-0)
Supplement: Supplementary file 3 — Additional file 3. Detailed sample description. [file 12936_2017_1769_MOESM3_ESM.docx]

Additional File 3: Detailed sample description

|  | Public  Health  Facility | CHW | Private  for-Profit  Facility | Pharmacy | Drug Store | General Retailer | Itinerant  Drug Vendor | **ALL**  **Outlets** |
| --- | --- | --- | --- | --- | --- | --- | --- | --- |
| **Number of outlets screened** | 95 | 463 | 172 | 479 | 15 | 6295 | 67 | 7586 |
| Census | 95 | 463 | 64 | 191 | 15 | 6295 | 67 | 7190 |
| Booster | 0 | 0 | 108 | 288 | 0 | 0 | 0 | 396 |
| **Number of outlets eligible and interviewed** | 91 | 167 | 94 | 331 | 6 | 30 | 5 | 724 |
| Census | 91 | 167 | 28 | 126 | 6 | 30 | 5 | 453 |
| Booster | 0 | 0 | 66 | 205 | 0 | 0 | 0 | 271 |
| **Number of outlets eligible but not interviewed** | 0 | 0 | 1 | 0 | 0 | 0 | 0 | 1 |
| Census | 0 | 0 | 0 | 0 | 0 | 0 | 0 | 0 |
| Booster | 0 | 0 | 1 | 0 | 0 | 0 | 0 | 1 |
| **Number of interviewed outlets with at least one anti-malarial in stock on the day of the survey** | 91 | 145 | 56 | 309 | 3 | 23 | 3 | 630 |
| Census | 91 | 145 | 19 | 118 | 3 | 23 | 3 | 402 |
| Booster | 0 | 0 | 37 | 191 | 0 | 0 | 0 | 228 |
| **Number of interviewed outlets with at least one anti-malarial in stock on the day of the survey or at least one anti-malarial reportedly in stock in the previous 3 months** | 91 | 164 | 58 | 327 | 4 | 30 | 5 | 679 |
| Census | 91 | 164 | 19 | 124 | 4 | 30 | 5 | 437 |
| Booster | 0 | 0 | 39 | 203 | 0 | 0 | 0 | 242 |
| **Number of interviewed outlets that provide malaria blood testing, but do not stock anti-malarial medicines** | 0 | 3 | 36 | 4 | 2 | 0 | 0 | 45 |
| Census | 0 | 3 | 9 | 2 | 2 | 0 | 0 | 16 |
| Booster | 0 | 0 | 27 | 2 | 0 | 0 | 0 | 29 |
| **Number of interviewed outlets that reported distributing anti-malarials in the week prior to the survey** | 14 | 14 | 14 | 76 | 1 | 6 | 1 | 126 |
| Census | 14 | 14 | 6 | 23 | 1 | 6 | 1 | 65 |
| Booster | 0 | 0 | 8 | 53 | 0 | 0 | 0 | 61 |
| **Number of interviewed outlets that reported providing/distributing a malaria diagnostic test in the week prior to the survey** | 49 | 45 | 48 | 63 | 1 | 1 | 0 | 207 |
| Census | 49 | 45 | 16 | 25 | 1 | 1 | 0 | 137 |
| Booster | 0 | 0 | 32 | 38 | 0 | 0 | 0 | 70 |
