# Supplementary material for: The malaria testing and treatment landscape in the southern Lao People’s Democratic Republic (PDR)
Source: Malar J. 2017 Apr 25;16:169. doi: 10.1186/s12936-017-1769-0 (PMC5404290; doi:10.1186/s12936-017-1769-0)
Supplement: Supplementary file 4 — Additional file 4. Provider perceptions regarding the most effective treatment for uncomplicated malaria. [file 12936_2017_1769_MOESM4_ESM.docx]

Additional File 4: Provider perceptions regarding the most effective treatment for uncomplicated malaria

|  | Private  For-Profit  Facility | Pharmacy | **ALL**  **Private** |
| --- | --- | --- | --- |
|  | %  (95% CI) | %  (95% CI) | %  (95% CI) |
| **Proportion of providers who:** | N=58 | N=327 | N=424 |
| State that chloroquine is most effective treatment uncomplicated P. falciparum / vivax malaria | 16.6  (8.4, 29.9) | 24.1  (18.8, 30.4) | 28.9  (23.4, 35.1) |
| State that chloroquine is their most recommended treatment for uncomplicated P. falciparum / vivax malaria | 20.4  (11.0, 34.8) | 29.2  (24.0, 35.0) | 32.7  (26.4, 39.8) |
