# Supplementary figures and images for: The malaria testing and treatment landscape in the southern Lao People’s Democratic Republic (PDR)
Source: Malar J. 2017 Apr 25;16:169. doi: 10.1186/s12936-017-1769-0 (PMC5404290; doi:10.1186/s12936-017-1769-0)

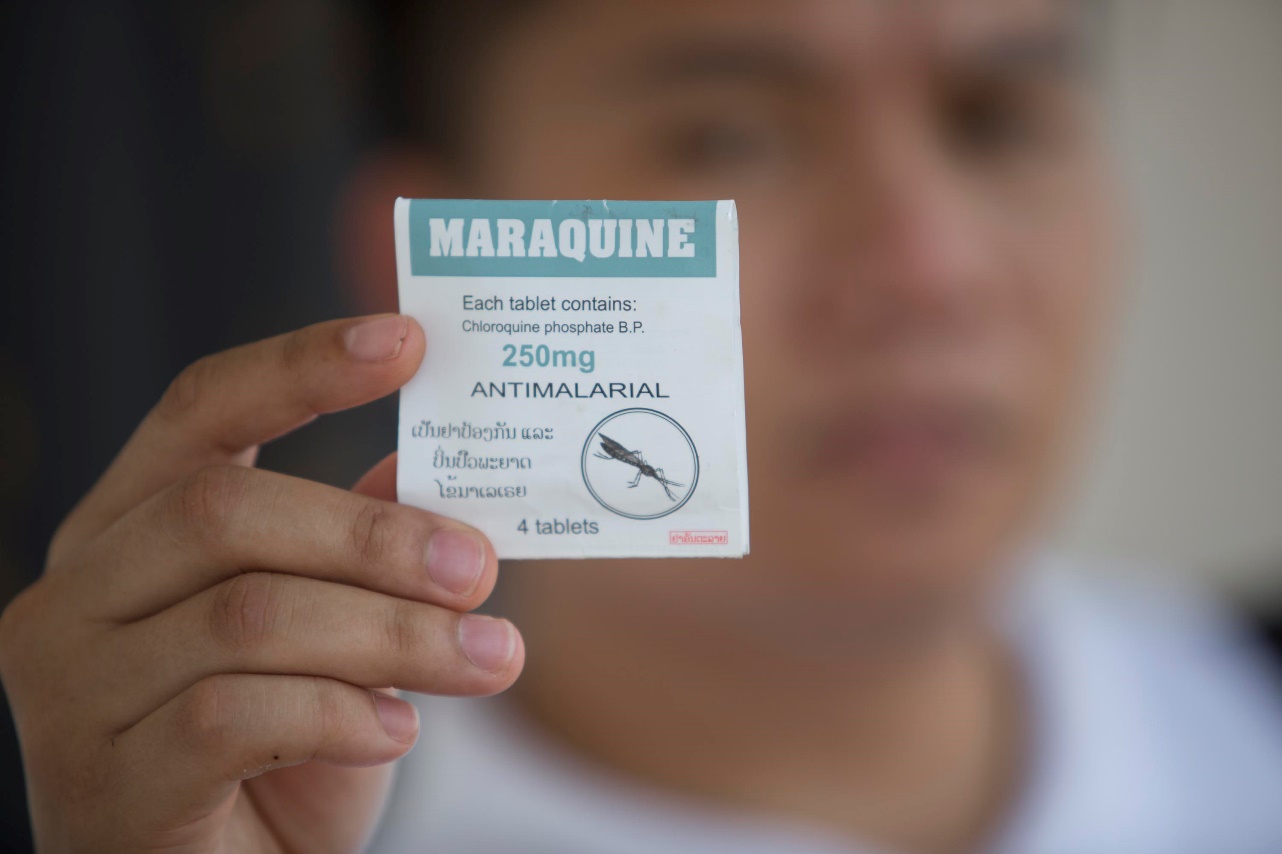
Additional File 5: Maraquine, a common brand of chloroquine

Supplement: Supplementary file 5 — Additional file 5. Maraquine®, a locally manufactured and popular anti-malarial. [file 12936_2017_1769_MOESM5_ESM.docx]
